# Exploring Colombian medicinal flora used in indigenous and campesino health systems for neuropsychiatric disorders and neuropharmacological potential: an ethnopharmacological review

**DOI:** 10.3389/fphar.2026.1729887

**Published:** 2026-03-11

**Authors:** Katrin Brache, Mauricio Diazgranados

**Affiliations:** 1 Department of Molecular Neuroscience, Swammerdam Institute for Life Sciences, Universiteit van Amsterdam, Amsterdam, Netherlands; 2 Research Department, Royal Botanic Gardens, Kew, Richmond, United Kingdom; 3 NYBG Science, New York Botanical Garden, New York, NY, United States

**Keywords:** central nervous system, Colombian medicinal plants, ethnopharmacology, indigenous health systems, indigenous/campesino knowledge, neuropsychiatric disorders, phytochemistry, psychopharmacology

## Abstract

Neuropsychiatric disorders affect nearly one billion people worldwide, yet many existing psychopharmacological treatments are limited by adverse effects, drug interactions, and variable efficacy. Ethnopharmacological knowledge embedded in Indigenous health systems offers important perspectives for understanding mental and neurological distress and for informing future research on central nervous system (CNS)–active plants. Colombia, one of the world’s most biodiverse countries, harbors a rich medicinal flora that is deeply embedded in Indigenous and rural cultural practices. This review synthesizes published ethnobotanical, phytochemical, and neuropharmacological literature on 42 Colombian plant species reported in Indigenous and local health systems to address conditions that may overlap with what biomedicine classifies as mental and neurological disorders. Within this review, traditional uses were analytically grouped into biomedical categories for comparative purposes, including psychoactive, stimulant, sedative, anxiolytic, and cognitive effects, while recognizing that these categories do not fully capture Indigenous epistemologies. Ayahuasca (locally called *yagé*) was the most frequently reported preparation, and *Nicotiana tabacum, Erythroxylum coca*, and *Aloysia citrodora* were the most commonly cited species. Leaves (38%), stems (14%), and roots (13%) were the most frequently used plant parts, most often prepared as decoctions (21%) and infusions (17%). Across the reviewed taxa, five lack phytochemical characterization, fourteen have demonstrated neuropharmacological activity in preclinical studies, and only seven have been examined in clinical contexts, underscoring substantial evidence gaps. Six species—*Iochroma fuchsioides*, *Brunfelsia grandiflora*, *Souroubea corallina*, *Tabernaemontana heterophylla*, *Psidium guajava*, and *Dianthera pectoralis*—emerged as recurrently cited across ethnobotanical and pharmacological sources, warranting further investigation. Overall, this review highlights both the potential and the limitations of existing evidence on Colombian plants, emphasizing the need for ethically grounded, collaborative research that respects Indigenous knowledge systems while advancing neuropharmacological understanding, cultural preservation, and biodiversity conservation.

## Graphical Abstract

**Figure:**
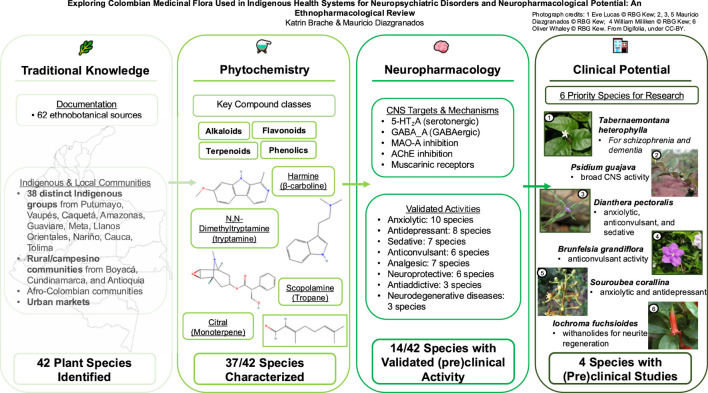

## 1 Introduction

Neuropsychiatric disorders represent a major and growing global public health challenge, affecting more than one billion people worldwide (GBD Mental Disorders Collaborators, 2022). This broad group of conditions includes disorders commonly classified in biomedicine as neurological and psychiatric, such as migraines, Parkinson’s disease, Alzheimer’s disease, schizophrenia, anxiety disorders, and depression. The COVID-19 pandemic has further exacerbated the prevalence and severity of many of these conditions, increasing both incidence and symptom burden (Asmundson and Taylor, 2020; Gobbi et al., 2020). In parallel, there has been a sustained global rise in the prescription of central nervous system (CNS)–acting drugs, including antidepressants, antipsychotics, anticonvulsants, anxiolytics, and mood stabilizers (Hálfdánarson et al., 2017; Barczyk et al., 2020; NHS Business Services Authority, 2023), aimed at symptom control, relapse prevention, and reduction of disability (World Health Organization, 2009).

Despite their clinical importance, many psychopharmacological treatments are associated with substantial limitations. These include adverse drug reactions, drug–drug interactions, variable effectiveness, delayed onset of action, and loss of efficacy over time (Bender et al., 2004; Sandson et al., 2005; Stroup and Gray, 2018). Reported first-episode response rates typically range between 50% and 80%, while tolerability and side-effect profiles vary widely across drug classes and individual compounds (Cipriani et al., 2018; Huhn et al., 2019; Kitagawa et al., 2024). Common adverse effects include sedation, dizziness, gastrointestinal disturbances, sexual dysfunction, visual impairment, nausea, and withdrawal or discontinuation syndromes (Kupeli Akkol et al., 2021). These challenges, together with relatively limited innovation in neuropharmacology over recent decades, have intensified interest in complementary and alternative perspectives for understanding and addressing mental and neurological distress.

Within this context, increasing attention has been directed toward plant-derived compounds with CNS activity. A major advantage of these bioactive compounds is their wide variety, which often possess selective biological actions targeting specific sites and pathways in the brain, resulting in fewer side effects of the medication (Clardy and Walsh, 2004; Itokawa et al., 2008). Numerous secondary metabolites, including alkaloids, flavonoids, and terpenoids, have demonstrated neuroactive properties in experimental settings, and taxa from several plant families—such as Amaryllidaceae, Papaveraceae, Solanaceae, and Ranunculaceae—have been investigated for potential relevance to neurodegenerative disorders, epilepsy, and other CNS-related conditions (Adewusi and Steenkamp, 2011; Kupeli Akkol et al., 2021). At the same time, plant-based therapies are not inherently risk-free: herb–drug interactions, inconsistent preparation methods, and limited clinical data can pose significant safety concerns (Posadzki et al., 2013). Consequently, plant-derived medicines should not be assumed to be safer or more effective than synthetic drugs, underscoring the need for critical, evidence-based assessment.

Beyond pharmacological considerations, the use of medicinal plants for conditions affecting the mind and nervous system has deep historical roots across many societies. Archaeological, ethnographic, and historical evidence indicates that psychoactive and medicinal plants have been used for millennia in diverse cultural contexts, including in Central and South America (Guerra-Doce, 2015; Miller et al., 2019). These practices are embedded within broader systems of knowledge, ritual, and social organization that cannot be reduced to biomedical disease categories alone. Understanding such practices therefore requires attention not only to pharmacological effects, but also to cultural, spiritual, and relational dimensions of healing.

Colombia provides a particularly important context for examining these intersections. As one of the most biodiverse countries in the world, Colombia harbors approximately 29,000 plant species, representing nearly 10% of global plant diversity (Diazgranados, 2022). It is estimated that more than 5,000 of these species are used medicinally or possess recognized medicinal properties (Diazgranados, 2022). Traditional plant-based healing practices remain widespread among Indigenous Peoples, Afro-Colombian communities, campesino populations, and urban populations, especially in regions such as the Amazon (including Putumayo, Caquetá, and Vaupés), the Cauca region, and the Orinoquía (Eastern Plains) (de Smet, 1983; Caro, 2004; Giraldo Quintero et al., 2015; Moncayo et al., 2022). Much of this knowledge has been transmitted orally across generations, reflecting sustained systems of practice rather than isolated or anecdotal use.

A significant portion of the documented knowledge on Colombian psychoactive and medicinal plants derives from the work of Richard Evans Schultes, whose extensive field research in the Colombian Amazon began in the 1940s and continued through the late 20th century. Schultes’ ethnobotanical studies documented not only plant taxa and preparations, but also the cosmologies, ritual contexts, and healing systems within which these plants are embedded (Schultes, 1988; Schultes and Raffauf, 1992). Among these are reports of plants used in relation to age-related cognitive changes and forms of senile dementia as understood within Amazonian communities (Schultes, 1993), an area that has received comparatively limited attention in subsequent scientific research.

**FIGURE 1:**
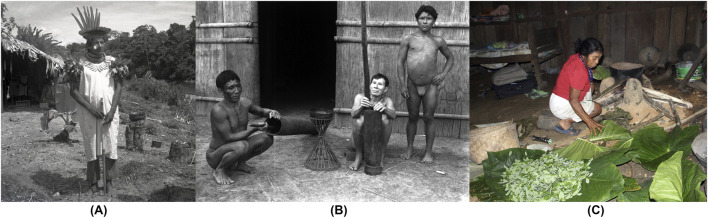
**(A)** Medicine man (“*taita*” or “*payés*”) and leader of the Cofanes tribe, well-versed in the use of a*yahuasca* (*Banisteriopsis caapi*) (Amazon Forest, Putumayo, Colombia; 14/December/1940; credits: José Cuatrecasas (I-636), with permission from the Smithsonian Institution). **(B)** Cubeos Indigenous pounding and sieving coca leaves (*Erythroxylum coca*) (Amazon Forest, Vaupés, Colombia; 12/December/1939; credits: José Cuatrecasas (I-636), with permission from the Smithsonian Institution). **(C)** U’wa taita’s wife drying coca leaves. A process that has been the same over many generations, as can be seen in **(B)**. (Andes foothills, Orinoco region, Boyacá, Colombia; by Mauricio Diazgranados (IMG-1834) with permission from U’wa).

Importantly, Indigenous health systems conceptualize illness and healing in ways that differ fundamentally from Western biomedical frameworks. Conditions that biomedicine classifies as psychological or neurological are often understood within relational ontologies that integrate physical, emotional, spiritual, social, and environmental dimensions (Sarasola, 2010). In many Indigenous Amazonian cosmologies, illness is not viewed as a purely biological malfunction, but as a disturbance in relationships among humans, plants, animals, spirits, and ancestors. Medicinal plants are therefore not regarded as inert chemical substances, but as active participants in healing processes, endowed with agency, intentionality, and pedagogical roles (Schultes and Raffauf, 1992; Llamazares et al., 2004). Knowledge regarding their use and preparation is maintained by community specialists—such as *taitas*, *abuelos*, *mamos*, *payé*, or *curanderos*—each embedded within specific cultural traditions. Terms such as “*shaman*” or “medicine man” are external labels commonly used in Western discourse and do not capture the diversity or specificity of these Indigenous roles (Schultes and Raffauf, 1992) (Figure 1).

In other regions of Colombia, including Andean communities and urban traditional markets, systems of classification such as the “hot–cold” framework remain influential. Within these systems, health is understood as a dynamic balance between opposing qualities, and treatment involves restoring equilibrium by administering remedies with properties considered opposite to those of the illness (Weller, 1983). Therapeutic decision-making is often highly contextual and individualized, meaning that similar biomedical diagnoses may be addressed with different plant-based treatments depending on perceived etiology, social circumstances, or spiritual factors.

Despite the continued importance of these practices, traditional medicine remains largely outside Colombia’s formal healthcare system. Many patients therefore navigate plural medical landscapes, drawing on both biomedical and traditional approaches as circumstances allow. From a research perspective, this plurality highlights the limitations of directly translating Indigenous therapeutic concepts into biomedical categories without careful contextualization.

Although Colombia’s medicinal flora and associated knowledge systems are exceptionally rich, many plants used in Indigenous and local health systems remain poorly characterized in terms of phytochemistry, neuropharmacology, and clinical evidence. Existing studies are unevenly distributed across species, often focus on isolated compounds rather than traditional preparations, and frequently lack methodological consistency. At the same time, ethical considerations—including intellectual ownership, benefit-sharing, cultural sensitivity, and biodiversity conservation—are increasingly recognized as central to research involving Indigenous knowledge systems.

The aim of this review is to synthesize published ethnobotanical, phytochemical, and neuropharmacological literature on Colombian medicinal plants used within Indigenous and campesino health systems for conditions that may overlap with what biomedicine classifies as CNS-related disorders. Specifically, this review i. documents reported traditional uses and preparation modes, ii. summarizes the current state of phytochemical and neuropharmacological evidence, and iii. identifies major evidence gaps and methodological limitations. By situating pharmacological findings within their cultural and epistemological contexts, this work seeks to inform ethically grounded future research while supporting Indigenous knowledge systems, cultural preservation, and biodiversity conservation.

## 2 Methods

### 2.1 Search strategy and data selection

A systematic literature search was conducted to identify ethnobotanical, phytochemical, and neuropharmacological studies reporting the use of medicinal plants in Colombia for conditions affecting the CNS. Search terms were constructed by combining four main conceptual domains: geographic scope (Colombia), ethnobotanical context, plant-based remedies, and CNS-related effects.

Searches were conducted in Google Scholar, PubMed, SciELO (Scientific Electronic Library Online), and Crossref, supplemented by targeted consultation of physical and digital collections from the Latin American Research and Documentation Centre (CEDLA), including ethnobotanical monographs and referenced books documenting medicinal and sacred plants traditionally used by Indigenous and rural communities in Colombia.

Search queries were formulated in both English and Spanish using Boolean operators (Table 1). No publication date limits were applied to ethnobotanical sources in order to capture historical documentation. In contrast, pharmacological and clinical studies were restricted to publications from 2000 to 2024, reflecting contemporary experimental and reporting standards.

**TABLE 1:** Search terms were used in Google Scholar, PubMed, Crossref, and SciELO databases.

Identification and selection	Location	Filtered plant species	English	Spanish
Records identification	Colombia	​	medicinal plant	planta* medicin*
			sacred plant	planta* sagrada
			ethnobotanical survey	entrevista etnobotánica
			ethnobot*	etnobot*
Species-level information search	Colombia	[Species name]	phytochemistry	fitoquímica
			pharmacology	farmacología
			neuropharmacology	neurofarmacología
			beneficial effect	efecto beneficioso
			traditional use	uso tradicional
			Medicinal plant	planta medicinal
			CNS	SNC
			central nervous system	sistema nervioso central

* Denotes the root stem of a search term, along with its morphological variations.

**FIGURE 2:**
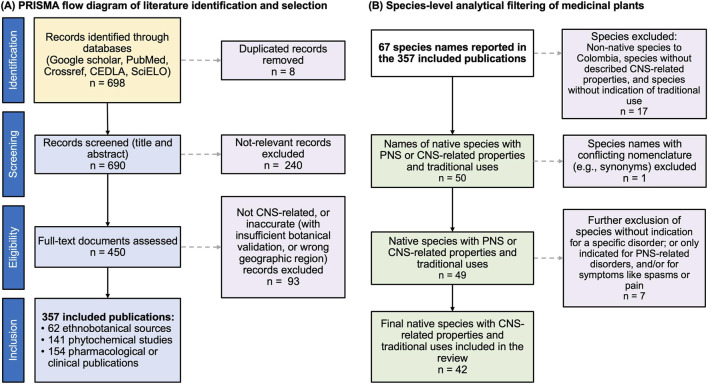
**(A)** PRISMA flow diagram illustrating the identification, screening, eligibility assessment, and inclusion of literature sources used in this review. The diagram summarizes the selection of ethnobotanical, phytochemical, and pharmacological or clinical publications reporting medicinal plants used in Colombia for central nervous system (CNS)–related indications. **(B)** Species-level analytical filtering applied after literature inclusion. The diagram summarizes the extraction and refinement of plant taxa reported across the included publications, resulting in a final set of 42 native plant species with documented traditional use in Colombia and relevance to CNS-related conditions. This analytical step is distinct from the PRISMA record-level screening shown in **(A)**. PNS: Peripheral Nervous System.

To identify plant species, we first searched for Ethnobotanical data to find information on the CNS, activities of identified plant species (Figures 2A,B). Search strings in English or Spanish were formed across four categories using the Boolean operator AND (e.g., “Colombia AND, medicinal plant”, “Colombia AND, *Adenocalymma schomburgkii* AND, farmacología”).

Literature searches in online databases were conducted using the software *Publish or Perish* (Harzing, 2010), which facilitates systematic retrieval of academic records from multiple sources. Additional relevant records were identified through backward and forward citation tracking of key publications and by reviewing related works by the same authors.

### 2.2 Screening and selection of records

The initial search yielded 698 records across all sources. After removal of duplicates (n = 8), 690 records were screened by title and abstract for relevance to the scope of the review. Screening was conducted independently by two reviewers.

Records were excluded at this stage if they did not address plant-based remedies, were unrelated to Colombia, or did not refer to CNS-related effects or uses. 450 records underwent full-text assessment; 357 publications met the eligibility criteria and were included in the qualitative synthesis. The full screening and selection process is summarized in a PRISMA flow diagram (Figure 2A).

### 2.3 Species-level analytical filtering

Following literature selection, a species-level analytical step was conducted to identify plant taxa reported across the included publications. This step was analytically distinct from the PRISMA screening of records.

An initial list of 67 plant species was extracted from the included literature. Species were excluded if they met any of the following criteria.

- lack of documented traditional use by Colombian Indigenous or rural communities,
- absence of CNS-related relevance or exclusive association with peripheral nervous system indications, or
- resolution as taxonomic synonyms.

After this filtering, 42 plant species were retained for inclusion in the review. The species-level analytical process is summarized separately from the PRISMA flow (Figure 2B).

### 2.4 Eligibility criteria

Ethnobotanical sources—including interviews, surveys, monographs, and institutional reports—were included if they documented medicinal or sacred plant use by Indigenous, Afro-Colombian, or campesino communities in Colombia and provided specific information on plant identity, preparation, and traditional indications.

Gray literature sources were included only when they met the following criteria.

- verifiable botanical identification (voucher specimens or later taxonomic validation),
- explicit geographic and cultural context,
- detailed preparation methods and traditional indications, and
- institutional or academic validation.

Only vascular plant species included in the *Useful Plants and Fungi of Colombia* (UPFC) project were considered. Botanical identities were validated using Plants of the World Online, ColPlantA (https://www.colplanta.org/) (Diazgranados et al., 2022), and the Medicinal Plant Names Services (MPNS; https://mpns.science.kew.org/). Non-vascular taxa, cultivated or naturalized plants without documented traditional use in Colombia, and records lacking taxonomic certainty were excluded.

Species native to Colombia or the wider Neotropics were included only when their traditional use by Colombian communities was explicitly documented, recognizing that cultural relevance is not determined by geographic origin alone.

Six species were identified as priorities based the following criteria: 1. documented traditional medicinal use with limited pharmacological validation; 2. endemic or restricted distribution; 3. robust traditional CNS indications across at least three independent sources; 4. preliminary phytochemical/pharmacological plausibility from related taxa or preliminary screening; and 5. critical evidence gaps relative to cultural importance.

### 2.5 Terminology and classification framework

For the purpose of this review, *neuropsychiatrically relevant plants* were defined as plant species with documented traditional uses or experimentally reported effects influencing CNS function, cognition, mood, perception, or behavior.

Ethnobotanical information was analyzed using a hybrid interpretive framework that integrates:

- traditional Indigenous or rural health concepts (e.g., *nervios*, trembling, age-related cognitive decline, insomnia), and
- biomedical classification systems (DSM-5 (American Psychiatric Association, 2013) or ICD-11 (World Health Organization, 2019) diagnostic categories, used solely for comparative and analytical purposes.

Eight primary CNS-related categories were derived from the convergence of ethnobotanical descriptions and pharmacological evidence by inductive analysis of all ethnobotanical sources and pharmacological publications. The eight primary categories serve to organize the ethnobotanical knowledge base. Classifications include: 1. Hallucinogenic - substances that cause hallucinations and alter perception of reality; 2. Stimulant - substances that increase alertness, energy, and elevate heart rate and blood pressure; 3. Anxiolytic - agents that reduce anxiety; 4. Sedative - depressants that induce drowsiness or sleep; and 5. Anticonvulsant - drugs that suppress seizures (Brunton and Knollmann, 2023). The categories ‘tranquilizing,’ ‘against age-related neurological symptoms,’ and ‘against neurological pain,’ meaning preparations that reduce pain on the somatosensory system (neuropathic) (Treede et al., 2015), represent translations of traditional ethnobotanical terminology from identified literature into biomedical constructs, mapped to standard pharmacological categories. These categories are analytical constructs to facilitate comparison with pharmacological literature and do not imply equivalence with Indigenous conceptualizations of illness or healing. In Colombian Indigenous traditions, health encompasses integrated mental, emotional, physical, and spiritual aspects, where illness stems from relational, cosmic, or spiritual disharmony rather than isolated biomedical pathologies.

For each species, all documented traditional uses from ethnobotanical sources were recorded and cross-referenced with pharmacological studies. When a traditional use matched the effect category (e.g., traditional “calming” use matched anxiolytic pharmacological effects), the species was assigned to that standard pharmacological effect category. When traditional uses did not align with standard pharmacological terminology (e.g., traditional “against nervios,” “for age-related forgetfulness”), species were assigned to the hybrid ethnobotanical-pharmacological categories. Although many species were documented with multiple traditional uses and pharmacological effects, each species was assigned to only one primary category based on which use was most frequently or prominently reported in the literature. Secondary uses and effects are documented in Supplementary Table S2 but are not reflected in the primary categorization used in the analysis.

Pharmacological outcomes reported in all included publications were classified into 15 CNS-related effect categories through inductive analysis. The fifteen categories comprehensively capture all pharmacological effects documented in the scientific literature, including those that may lack traditional ethnobotanical documentation but have been scientifically validated. These categories represent the full range of CNS effects documented across the pharmacological literature reviewed. All identified effects were retained. When species were associated with multiple effects, the most frequently reported effect was designated as the primary outcome. The 15 categories identified were: 1. Sedative; 2. Hallucinogenic; 3. Stimulant; 4. Anxiolytic; 5. Anticonvulsant; 6. Antidepressant - agents alleviating depression by modulating monoaminergic neurotransmission; 7. Antiaddictive - compounds reducing substance dependence or craving behaviors; 8. Anti-Alzheimer’s - agents targeting cognitive decline; 9. Anti-Parkinson’s - drugs addressing motor symptoms caused by Parkinson’s disease; 10. Neuroprotective - agents preventing neuronal damage or death; 11. Inducing neurogenesis or neuroplasticity - compounds promoting neurogenesis or neuroplasticity in the CNS; 12. Analgesic - pain-relieving agents acting on central pain pathways; 13. Anesthetic - drugs inducing reversible loss of sensation or consciousness; 14. Cognitive enhancer - agents improving memory, attention, or executive functions; and 15. Anti-headache - compounds preventing or treating headache disorders (migraine, tension-type), applied consistently across studies (Brunton and Knollmann, 2023).

### 2.6 Risk-of-bias and evidence assessment

Pharmacological and clinical studies were evaluated using the Oxford Centre for Evidence-Based Medicine Levels of Evidence (2011) to categorize the strength of evidence based on study design. Studies were assigned to Levels 1a–4, distinguishing between clinical, preclinical, and *in vitro* evidence.

Risk-of-bias assessment was conducted using established tools appropriate to study type. Randomized controlled trials were assessed using the Cochrane Risk-of-Bias tool 2.0 (RoB 2) (Sterne et al., 2019), while preclinical *in vivo* studies were evaluated using the SYRCLE risk-of-bias tool (Hooijmans et al., 2014). Each study was rated as having low, moderate, or high risk of bias across relevant domains.

### 2.7 Data synthesis and analysis

Descriptive analyses were conducted to summarize patterns of traditional use, plant parts utilized, preparation methods, taxonomic distribution, and CNS-related categories. Frequencies and percentages were calculated using Microsoft Excel to provide visual representations of the curated data.

For ethnobotanical evidence, outcomes consisted of counts and proportions of species reported for each traditional use category. For pharmacological studies, outcomes included reported behavioral, neurochemical, or clinical effects, interpreted in relation to evidence level and risk-of-bias assessment.

Formal meta-analysis and publication-bias assessment were not conducted, as the synthesis integrated heterogeneous evidence types unsuitable for quantitative aggregation.

## 3 Results

### 3.1 Literature identification and selection process

The literature search identified 698 records across Google Scholar, PubMed, Crossref, SciELO, and CEDLA. After removing 8 duplicates, 690 records were screened by title and abstract, and 240 records were excluded as not relevant. 450 full-text articles were assessed for eligibility, and 93 records were excluded because they did not report CNS-related uses/outcomes and/or did not meet inclusion criteria (e.g., insufficient botanical validation or lack of a Colombian geographic context). A total of 357 publications were included in the qualitative synthesis, comprising 62 ethnobotanical sources, 141 phytochemical studies, and 154 pharmacological or clinical publications (Figure 2A).

Following record inclusion, a separate species-level analytical filtering step was conducted (Figure 2B). From the 357 included publications, 67 taxa were extracted. Seventeen taxa were excluded because they were non-native to Colombia, lacked documented traditional use in Colombia, and/or lacked CNS-related relevance. This yielded 50 taxa with reported traditional use and potential PNS/CNS relevance. One additional taxon was excluded due to conflicting nomenclature (synonymy), leaving 49 taxa. A further 7 taxa were excluded because the reported uses were non-specific (e.g., “for the nervous system” without a defined indication) and/or were limited to peripheral nervous system indications or symptoms (e.g., spasms or pain) without CNS relevance. This resulted in a final set of 42 native species with CNS-related properties and documented traditional uses included in the review (Figure 2B).

An overview of study counts by evidence domain (including clinical trial presence) are summarized in Table 2, and the research distribution by evidence domain across all included species is presented in Table 3.

**TABLE 2:** Distribution of publications by research domain across 42 plant species with central nervous system (CNS)–related activity in Colombia.

Research domain	Nr. of species	Nr. of publications
Ethnobotany	42	62
Phytochemistry	37	141
Neuropharmacology	14	146
Preclinical evidence	9	46
Clinical Trials	5	7

The table summarizes the number of ethnobotanical, phytochemical, and pharmacological or clinical publications identified for each species. Detailed source information is provided in Supplementary Tables S1, S4.

**TABLE 3:** Relative intensity of published research across evidence domains for the 42 selected plant species.

Selected medicinal plants	Nr. of publications in PubMed	Nr. of clinical trials	Nr. of ethnobotanical studies from Colombia	Nr. of phytochemical studies	Nr. of neuro- pharmacological studies (pre-clinical and clinical)
*Adenocalymma schomburgkii* (DC.) L.G.Lohmann	0	0	1	0	0
*Aloysia citrodora* Paláu	2	5	5	3	4
*Anadenanthera peregrina* (L.) Speg*.*	4	0	4	3	0
*Banisteriopsis caapi* (Spruce ex Griseb.) Morton	1	4	5	4	5
*Brugmansia arborea* (L.) Sweet*	3	0	2	5	2
*Brugmansia aurea* Lagerh.	1	0	2	5	0
*Brugmansia × candida* Pers.	2	0	4	5	0
*Brugmansia sanguinea* (Ruiz & Pav.) D.Don	1	0	4	5	0
*Brunfelsia grandiflora* D.Don	1	0	2	3	0
*Coriaria ruscifolia* L.	1	0	2	2	0
*Datura stramonium* L.*	2	1	2	3	2
*Dianthera pectoralis* (Jacq.) J.F. Gmel.*	4	0	3	2	2
*Diplopterys cabrerana* (Cuatrec.) B. Gates	1	3	4	2	4
*Drimys granadensis* L.f.	1	0	1	2	0
*Erythroxylum coca* Lam.	4	0	5	3	1
*Galactophora crassifolia* (Müll.Arg.) Woodson	0	0	1	1	0
*Hyptis brachiata* Briq.	0	0	1	1	0
*Ilex guayusa* Loes.	1	0	2	4	0
*Iochroma fuchsioides* (Bonpl.) Miers	1	0	1	1	0
*Irlbachia nemorosa* (Willd. ex Schult.) Merr.	2	0	1	0	0
*Juglans neotropica* Diels	1	0	2	3	0
*Justicia idiogenes* Leonard	0	0	1	0	0
*Lepechinia bullata* (Kunth) Epling	1	0	2	2	0
*Lippia alba* (Mill.) N.E.Br. ex Britton & P.Wilson	3	2	4	5	3
*Mandevilla steyermarkii* Woodson	0	0	1	1	0
*Mimosa albida* Humb. & Bonpl. ex Willd.	1	0	1	1	0
*Myrcianthes leucoxyla* (Ortega) McVaugh	1	0	2	2	0
*Nicotiana tabacum* L.	5	0	5	5	3
*Ocimum campechianum* Mill.	1	0	1	2	0
*Passiflora edulis* Sims	3	3	2	4	3
*Paullinia yoco* R.E.Schult. & Killip	1	0	2	2	0
*Psidium guajava* L.*	3	0	3	4	1
*Psychotria guianensis* (Aubl.) Clos	4	0	2	1	0
*Psychotria viridis* Ruiz & Pav.	2	4	5	4	5
*Smallanthus pyramidalis* (Triana) H.Rob.	0	0	1	1	0
*Souroubea corallina* (Mart.) de Roon	0	0	1	0	0
*Tabernaemontana heterophylla* Vahl*	4	0	1	2	1
*Unonopsis stipitata* Diels	0	0	1	2	0
*Unonopsis veneficiorum* (Mart.) R.E.Fr.	0	0	2	2	0
*Valeriana clematitis* Kunth*	4	0	2	2	1
*Valeriana scandens* L.	1	0	2	0	0
*Virola calophylla* Warb (Schultes)	1	0	2	3	0

Published studies per species is summarized on a semi-quantitative scale from 0 to 5, where 0 (white) indicates no identified studies, 1 meaning <5 publications, 2 meaning 5–10 publications, 3 meaning 10–20 publication, 4 meaning 20–30 publications, and 5 (dark red) indicates the highest research intensity meaning >30 publications. The distribution highlights that most species remain poorly studied beyond ethnobotanical documentation, underscoring major gaps in phytochemical and neuropharmacological research.

*Plants with promising pharmacological activity on the CNS, highlighted in this review.

Based on the 62 ethnobotanical sources, this review documents 42 plant species reported in Indigenous, Afro-Colombian, and campesino health systems in Colombia for uses that may overlap with what biomedicine classifies as CNS-related conditions and effects. Across the included literature, phytochemical information was available for 37 species (from 141 phytochemical studies), while 5 species lacked published phytochemical characterization (Table 3). Pharmacological or clinical studies reporting CNS-related outcomes were identified for 14 species (from 154 pharmacological/clinical publications), whereas 28 species had no identified neuropharmacological studies meeting the inclusion criteria (Table 3; Table 4).

**TABLE 4:** Overview of important neuropharmacological studies of selected medicinal plants.

Plant species	Plant part	Isolated component(s)/extracts	Study design/type	Level of evidence*	Sample size, dose, duration	Risk-of-Bias**	Main neurophar-macological activity	Main findings	Mechanism description	Safety notes	Ref.
*Aloysia citrodora* Paláu	Leaves	Ethanolic and aqueous extracts, verbascoside (VS)	Animal study (*in vivo*)	2a	Multiple dose groups from 50 to 200 mg/kg + controls in mice	Moderate	Anxiolytic, sedative, hypnotic, muscle relaxant effects	Extracts and VS increased open arm entries in elevated plus maze (EPM) and showed interaction with GABA-A receptors, with dose-dependent anxiolytic and hypnotic effects	Modulation of GABA-A receptor indicated by flumazenil inhibition, antioxidant, anti-inflammatory	No adverse effects reported in the study, but further safety evaluation needed for clinical translation	Razavi et al. (2017)
	Leaves	Purified phenylpropanoids extract	Randomized, double-blind, placebo-controlled clinical trial	1b	40 patients; 500 mg/day; 8 weeks, followed by a 4-week washout period	Low	Anxiolytic, Sedative (improved sleep quality)	Significant reduction in perceived stress and better sleep quality in treated individuals compared to placebo, more pronounced in women	Exert its effect through the GABA system by binding to the GABA-A receptor in a similar fashion as benzodiazepines, modulation of cAMP and calcium channels, increased the expression of BDNF, serotonin, noradrenaline and dopamine	No adverse effects reported	Martínez-Rodríguez et al. (2022)
	Leaves	Essential oil	Randomized controlled trial (RCT)	1b	95 patients; single inhalation EO	Low (minor concern about olfactory blinding)	Anxiolytic	Carvone: main constituent, Significant reductions in state anxiety scores post-inhalation of essential oil compared to control (p < 0.05), large effect size (Cohen’s d = 1.06); no adverse effects reported	Enhances GABA-A receptor activity; antioxidant and anti-inflammatory effects contribute to anxiolytic action	No adverse effects reported	Alvarado-García et al. (2021)
	Leaves	Hydroal-coholic extract	randomized, double-blind, placebo-controlled clinical trial	1b	100 patients; 500 mg/day, 4 weeks	Low	Against insomnia, Sedative	Sleep quality (Pittsburgh Sleep Quality Index) and Insomnia Severity Index (ISI) significantly improved vs. placebo (p < 0.001) at 2 and 4 weeks	Enhances GABAergic signaling	No adverse effects reported	Afrasiabian et al. (2019)
	Leaves	Essential oil	Preclinical, *in vitro* and *in vivo* animal study	2a	Animal/cell lines); 0.001–0.01 mg/mL (cells)	Moderate	Neuro-protective, Anxiolytic candidate	EO showed dose-dependent protection against oxidative and β-amyloid neurotoxicity in neuronal cell line and modulated CNS receptor binding	Enhances GABAergic signaling; inhibits oxidative stress; iron chelation	No toxicity reported	Abuhamdah et al. (2015)
	Leaves	Ethanolic extract and Vs.	Animal study (*in vivo*)	2a	Male mice; 3–5 groups per does; 100, 200, 400 mg/kg orally	Moderate	Anti-depressant, Anxiolytic, Relaxant	Dose-dependent anxiolytic and relaxant effects in mice; significant behavioral improvements observed	Enhances GABAergic neurotransmission; relaxant effect via calcium channel modulation; Antidepressant-like effect through expression of 5-HAT, NA, DO and BDNF	No adverse effects reported	Sabti et al. (2019)
	Leaves	Ethanolic extract	Animal study (*in vivo*)	2b	Male mice, 50 and 100 mg/kg	Moderate	Anti-convulsive	Dose-dependent reduction of convulsions (duration) in electro- and chemoconvulsant models in mice	Modulation of neuronal excitability; possible GABAergic involvement	No toxicity reported	Rashidian et al. (2016)
	Aerial parts	Essential oil	Animal study (*in vivo*)	2b	Juvenile silver catfish, 10, 30, 50, 70, 100 μL/L vapor exposure	Moderate	Sedative, Anesthetic	Dose-dependent sedation and anesthetic induction in fish with good recovery times, 200 μL: best response time to anesthesia	CNS depressant effects via GABAergic modulation likely	No reported toxicity	Parodi et al. (2014)
Ayahuasca/Yagé; *Banisteriopsis caapi* (Spruce ex Griseb.) Morton + *Psychotria viridis* Ruiz and Pav. OR *Diplopterys cabrerana* (Cuatrec.) B. Gates	Stalks of *B. caapi* with leaves of *P. viridis*	Decoction: boiled and concentrated for a few hours; β-carbolines and N, N-DMT	Open-label clinical trial	1b	17 patients; Single oral dose (∼2 mL/kg brew) follow-up up to 21 days	Low (for open-label design; well-executed within randomization limitations)	Anti-depressive, Anxiolytic	Rapid antidepressant effects (HAM-D, MADRS scales) within hours, sustained at 21 days post-single dose; response rate 64% vs. 27% placebo at day 7 (p = 0.04); increased blood perfusion in brain regions associated with the regulation of mood and emotions	MAO-A inhibition by β-carbolines allows DMT to activate multiple serotonin receptors (5-HT1A, 5-HT2A, 5-HT2C), modulating brain circuits involved in mood regulation, default mode network, neuroplasticity, and emotional processing, which contribute to rapid antidepressant effects	No serious adverse events; transient mild psychedelic effects; small sample size limits safety generalization; Psychedelic effects require close monitoring, thorough screening, and controlled settings to minimize risks	Sanches et al. (2016)
	Not specified	Preparation method not specified	Randomized double-blind placebo-controlled trial	1b	29 patients; Single oral dose (∼2 mL/kg brew), follow-up at 1, 2, 7, and 21 days	Low	Anti-depressive, anxiolytic activity	Significant rapid antidepressant effects after a single dose on MADRS and HAM-D scales at 1-, 2-, 7-, and 21-days post-dose, large effect sizes	MAO-A inhibition by β-carbolines enables DMT to activate 5-HT1A, 5-HT2A, 5-HT2C receptors, modulating brain regions hypoactive in depression, enhancing neuroplasticity and emotional regulation	Well-monitored setting; no serious adverse events; transient mild psychedelic effects; small sample limits generalizability	Palhano-Fontes et al. (2019)
	Not specified	Preparation method not specified	Naturalistic observational study in therapeutic community setting	3	11,912 people between 2017–2020; Variable ceremonial doses	Moderate (observational/selection bias)	Anti-depressive, anxiolytic, substance use reduction	Significant improvements in depression (78%), anxiety (70%), substance use severity, quality of life at 1-year post-treatment	Psychedelic activation of serotonin receptors combined with contextual/spiritual factors promoting neuroplasticity and psychological wellbeing	No serious adverse events reported; individual variation in experience noted; importance of ceremonial context and psychological support emphasized	Sarris et al. (2021)
	Not specified	Preparation method not specified	Observational study in inpatient addiction treatment	3	36 male participants; doses not specified	Moderate (high selection bias and confounding)	Antiaddictive, anxiolytic	Significant reductions in addiction severity scores, Significant improvements of mental and emotional health (reductions in perceived stress, mental illness symptoms, and craving) and increased meaning and purpose in life	Serotonergic modulation and psychological/spiritual support synergistically support recovery	No serious adverse events reported; treatment involved professional support	O’Shaughnessy et al. (2021)
	*B. caapi* root and leaves of *P. viridis*	Preparation method not specified	Naturalistic observational study	3	12 participants in 2 expert led ceremonies; received varying amounts; amounts or relative concentrations not known	Moderate (high selection bias and confounding)	Antiaddictive, anxiolytic	Significant improvements in addiction severity, depression, anxiety, and quality of life at 6-month follow-up, self-reported decline of alcohol, tobacco and cocaine use with significant reduction of problematic cocaine use, no changes in cannabis and opiate use	Psychedelic activation of serotonin 5-HT2A receptors by DMT facilitates emotional processing and psychological insight, which combined with therapeutic and ritual support, promotes addiction recovery and mental health improvement	No serious adverse events reported; integrative therapeutic context essential	Thomas et al. (2013)
	Not specified	Preparation method not specified	Cross-sectional neuroimaging study	3	22 patients, traditional use, dose not specified	Moderate (selection bias)	Structural brain changes linked to serotonergic modulation	Significantly reduced cortical thickness in posterior cingulate cortex correlated inversely with length and intensity of ayahuasca use	Serotonin 5-HT2A receptor activation by N, N-DMT induces neuroplastic remodeling in midline brain regions, including the posterior cingulate cortex, affecting self-referential processing and consciousness	No adverse effects reported, consistent with long-term traditional use	Bouso et al. (2015)
	Not specified	Preparation method not specified	Experimental within-subject	2b	26 participants; single ceremonial dose (not specified)	Low (no placebo, confounding)	Improved cognitive abilities, enhanced creative divergent thinking	Acute ayahuasca administration decreased convergent thinking but enhanced divergent (creative) thinking	Modulates brain networks including DMN, CEN, and salience network through serotonergic pathways	Well tolerated; transient subjective effects	Kuypers et al. (2016)
	Not specified	Preparation method not specified	Observational study in ritual use	3	23 participants; Traditional ceremonial doses (not specified)	Moderate (high selection bias, self-reported data)	Improve overall health, mood enhancing, anxiolytic	Ayahuasca users had a significant reduction of anxiety and improvements in mood and overall mental health observed post-ceremony, change in attitude (more confidence and optimism), decrease in physical pain	5-HT2A receptor activation by DMT with MAO-A inhibition	Well tolerated; no serious adverse events	Barbosa et al. (2009)
	Not specified	Preparation method not specified	Experimental study in experienced users	2b	24 participants; ceremonial dose (not specified)	Low (high bias risk in blinding and confounding)	Enhancement of mindfulness and emotional regulation	Significant reduction in judgmental processing of experiences and in inner reactivity, significant increase in decentering ability (classic goals of mindfulness training)	Serotonergic activation promotes interoceptive awareness and emotional processing	Well tolerated; no serious adverse events reported	Soler et al. (2016)
*Banisteriopsis caapi* (Spruce ex Griseb.) Morton	Fresh leaves, stems, and large branches (bark) and mature stems	Aqueous extract; β-carbolines (harmine, harmaline, THH), proanthocyanidins	Chemical and pharmacological profiling	4	N/A	N/A	MAO inhibition, antioxidant	Extract shows potent MAO-A inhibition and antioxidant capacity; basis for potential neuroprotective effects in Parkinson’s	Potent MAO-A inhibition by β-carbolines (harmine, harmaline, THH) coupled with antioxidant proanthocyanidins reduce neurodegeneration and modulate neurotransmitter levels relevant to Parkinson’s disease	Traditional use suggests tolerability	Samoylenko et al. (2010)
	Stem	Isolated alkaloids from Stem extract: Harmine, harmaline, tetrahydroharmine	*In vitro* cell culture study	4	N/A	N/A	Neurogenesis stimulation and neuroprotection	Harmine, tetrahydroharmine, and harmaline stimulate adult neurogenesis, in progenitor cells compounds stimulate stem cell migration, proliferation, and differentiation into adult neurons	β-carbolines harmine, harmaline, and tetrahydroharmine stimulate adult neural stem cell proliferation, migration, and differentiation into neurons and glia, promoting neurogenesis and neuroplasticity that may underlie therapeutic effects in neurodegenerative disorders	Preclinical; safety in humans unknown	Morales-García et al. (2017)
	Vine, Stem	Liquid extract, β-carbolines	Randomized controlled clinical trial	1b	30 *de novo* diagnosed Parkinson’s patients; 200 mL single dose, effects 4 h post-treatment	Moderate (unclear randomization, Single dose; blinding unclear)	Motor function, tremor severity (likely against Parkinson’s)	Significant improvement in motor function, maximal beneficial effects at 2 h post-treatment until 4 h, tremor was not improved	Symptomatic benefits could be due to harmalines glutamate receptor antagonist actions; MAO-A inhibition reduces dopamine degradation; antioxidants protect neurons from oxidative stress	Mild side effects (diarrhea, nausea, transient hallucinations) betacarbolineshave been shown to cause hyperthermia, tingling sensations, nausea, vomiting, tremors, and learning and memory deficits	Serrano-Dueñas et al. (2001)
*Brugmansia arborea* (L.) Sweet	Aerial parts (leaves and flowers)	Methanol extract; Tropane alkaloids (atropine, apoatropine, 3α-tigloil-oxitropane)	Experimental animal study (Conditioned place preference in mice)	3	Sample size not specified; 7.5–60 mg/kg (methanol extract)	Moderate	Antiaddictive, dopaminergic and cholinergic receptor modulation	Extract blocked reinforcing effects of morphine and cocaine; attenuated morphine-induced hyperactivity dose-dependently	Antagonism of D1/D2 dopamine and muscarinic acetylcholine receptors mediates inhibition of drug rewarding and motor effects	No motor impairment caused; well tolerated at tested doses	Bracci et al. (2013)
	Aerial parts (leaves and flowers)	Methanol extract, pure tropane alkaloids (atropine, apoatropine, 3α-tigloyl-oxitropane)	Experimental animal study	3	8–12 per group; 7.5, 15, 30 mg/kg (methanol extract)	Moderate	Modulation of opioid tolerance and dependence	Extract reduced expression (acute phase) but not acquisition (development) of morphine tolerance; attenuated withdrawal symptoms dose-dependently	Likely antagonism of muscarinic and other receptors modulates opioid system, reducing tolerance and dependence	Well tolerated at tested doses	Mattioli et al. (2012)
	Aerial parts	Aqueous extract; Alkaloids and secondary metabolites	*In vitro* receptor binding study	4	N/A	N/A	Differential affinity for dopamine and serotonin receptors	Extract showed affinity for muscarinic and D2 receptors and weak affinity for the serotoninergic receptors; receptor interaction could be useful in the treatment of PD and Schizophrenia	Antagonism of central muscarinic acetylcholine receptors reduces cholinergic neurotransmission; modulation of dopaminergic pathways likely attenuates morphine dependence and withdrawal symptoms	Not reported	Nencini et al. (2006)
*Datura stramonium* L.	1: seed, 2: root, 3: rhizome, 4: not specified	Capsule: *D. stramonium* (1) (43%) + *Rheum palmatum* (2) + *Zingiber officinale* (3) + *Acacia senegal* (4)	Randomized placebo-controlled trial	1b	81 opioid-dependent patients; 1 capsule/day; 12 weeks	Low (cannot isolate Datura effect)	Anxiolytic, Anti-depressant, Antiaddictive	Significant reduction in opioid craving, use, depression, anxiety; higher retention in treatment group; no opioid receptor agonism observed	Contains anticholinergic alkaloids (mainly from *D. stramonium*) that block central muscarinic receptors, modulating neurotransmitter systems involved in craving and withdrawal; combined with anti-inflammatory plant metabolites, this reduces opioid craving, withdrawal severity, and relapse risk	Well tolerated; Side effects were similar between the treatment and placebo groups	Moosavyzadeh et al. (2020)
	Not specified	Herbal fluid extract among others *D. Stramonium*	Experimental animal study	3	35 Wister rats; 24 drops in 2L of water	Moderate	Anti-convulsant, neuroprotective	Reduced spontaneous seizure development and provided neuroprotection in lithium-pilocarpine rat model	Tropane alkaloids (atropine, scopolamine, hyoscyamine) competitively antagonize central and peripheral muscarinic acetylcholine receptors, producing anticholinergic, sedative, and hallucinogenic effects by inhibiting cholinergic neurotransmission	Toxicity risk at high doses; careful dosage required	Peredery and Persinger (2004)
*Dianthera pectoralis* (Jacq.) J.F. Gmel.	Aerial parts	Aqueous extract, high amount of coumarin and umbelliferone	Experimental animal study	2b	Male Swiss rats (8–12 per group); 50, 100 and 200 mg/kg	Moderate (unclear allocation/blinding/sequence generation)	Anxiolytic-like effects	Produced anxiolytic-like effects in mice behavioral tests; effects blocked by flumazenil indicating GABA-benzodiazepine receptor involvement	Modulates GABAergic system via benzodiazepine binding sites impacting anxiety regulation	low toxicity of hydroalcoholic extract; No sedative or neuromuscular blocking effects reported	Venâncio et al. (2011)
	Leaves/stems	Ethanol extracts	*In vitro* receptor and enzyme assays	4	Not reported	Moderate (no specific reporting for each species, unclear state of replicates)	Anti-convulsive, Anxiolytic	The Acanthaceae family showed strong GABA-T inhibition and GABA(A)-BZD receptor binding correlating with traditional antiepileptic and anxiolytic use	Likely modulation of GABAergic system through enzyme inhibition and receptor agonism	Traditional use suggests safety; detailed toxicology not reported	Awad et al. (2009)
	Aerial parts (leaves)	Aqueous extract, hydro-alcoholic extract, dry extract (spray-dried) containing coumarins	Experimental animal study	2a	24 male mice; 64.8, 129.0, 259.0 mg/kg (oral)	Moderate (Unclear sequence generation/allocation/housing and blinding)	Sedative, Anti-aggressive	Dose-dependent reduction in spontaneous locomotor activity, rearing and aggressiveness; increased sleep number and duration similar to diazepam	Modulation of GABAergic neurotransmission or other neurochemical pathways enhancing sedation and sleep similar to diazepam, contributing to a calming effect on CNS excitability	Classified as non-toxic in toxicology study	Rodríguez-Chanfrau et al. (2013)
	Aerial parts	Standardized aqueous extract (SEJP)	Experimental animal study	2a	40 mice/200 mg/kg	Moderate (unclear sequence generation/Allocation/housing/blinding)	Modulation of central monoaminergic neurotransmitters	SEJP decreased dopamine, noradrenaline and serotonin levels in prefrontal cortex and hippocampus; altered metabolites in striatum	Modulation of monoaminergic systems (dopamine, noradrenaline, serotonin) which may underlie anxiolytic and mood effects	Safety was confirmed with no adverse outcomes reported	Venâncio et al. (2019)
*Lippia alba* (Mill.) N.E.Br. ex Britton and P.Wilson	Leaves	Hydro-ethanolic extract; (geranial and carvenone chemotype)	Prospective, phase 2 clinical cohort study	2b	21 women (diagnosed migraine)	Moderate (unblinding, No placebo; op; open label)	Against headaches	Significant reduction in headache frequency, pain intensity, missed days; >70% had ≥50% reduction	Geranial and carvenone reduce migraine symptoms by modulating neurovascular inflammation and nociceptive ion channels, decreasing trigeminal nerve activation and vasoactive peptide release	No side effects reported	Carmona et al. (2013)
	Leaves	Hydro-alcoholic extract; (geranial and carvenone chemotype)	Prospective, phase 2 clinical cohort study	2b	21 patients; dose not specified (oral)	Moderate (unblinding, No placebo; open label)	Against headaches	>80% patients had ≥50% reduction in headache frequency and intensity; decreased missed days	Geranial and carvenone modulate neurovascular inflammation and trigeminal activation	No side effects were recorded	Conde et al. (2011)
	Leaves	Essential oil; (carvone 64.5%)	Randomized clinical study	2b	95 adults, dose not specified (inhalation)	Low (minor blinding concern)	Anxiolytic	Carvone: main constituent, Significant reduction in state anxiety post-inhalation; larger effect size compared to *Lippia citriodora*	Carvone acts as positive allosteric modulator of GABA_A receptors	No adverse effects reported	Alvarado-García et al. (2021)
	Leaves	Liquid and spray-dried extracts; Non-volatile fraction including flavonoids	Experimental animal study	2a	Mice; 200 mg/kg	Moderate	Sedative, myorelaxant, anticonvulsant CNS effects	80% ethanol extract: most significant myorelaxant and sedative activity and highest flavonoid content, only one spray-dried powder showed sedative activity	Effects attributed to flavonoids and GABAergic modulation; spray-dried powders retained activity	No toxicity reported	Zétola et al. (2002)
	Leaves	Essential oil (contains carvone, linalool, β-caryophyllene)	Experimental animal study	2a	6 silver catfish; (50, 100, and 300 µL/L	Moderate	Anesthetic	Anesthetic effect potentiated by diazepam, antagonized by flumazenil; shows involvement of GABA_A receptor complex	Involvement of benzodiazepine (BDZ) binding sites coupled to GABA_A receptors mediates anesthetic properties	No toxicity reported	Heldwein et al. (2012)
	Leaves	Essential oil from 3 chemotypes including citral, carvone, limonene	Experimental animal study	2a	female Swiss mice; between 100 and 400 mg/k g)	Moderate	Anti-convulsant	Significant increase in latency time and percentage of survival for the first convulsion with EO and citral, limonene, and myrcene	EOs anticonvulsant effects have a similar pharmacological profile as DZP-like drugs; Likely mediated via modulation of CNS excitability and ion channel inhibition	No adverse effects reported	de Barros et al. (2000)
*Nicotiana tabacum* L.	Stem	Aqueous extract used as bio reducing agent to synthesize silver nanoparticles	*In vitro* cell culture	4	Rat PC-12 neuronal cells	Moderate	Neuro-protective	Tobacco stem-derived silver nanoparticles protected PC-12 cells from cytotoxicity and oxidative stress; improved cell viability and mitochondrial function	Neuroprotective effect likely due to antioxidant properties of silver nanoparticles coupled with bioactive phytochemicals	No major cytotoxicity at tested doses; effects dose-dependent	Sharma et al. (2017)
	Leaves	Two essential oils	Experimental animal study	2a	18 groups of mice; 250, 500, 1,000, and 2,000 mg/kg	Moderate	Anxiolytic	Both oils increased time in light chamber and open arms in behavioral tests, reduced salivary corticosterone	Anxiolytic effects likely mediated by modulation of the hypothalamic-pituitary-adrenal (HPA) axis through significant reduction in salivary corticosterone levels, leading to decreased stress hormone-induced anxiety behaviors	High doses lead to reduced activities and appetite or death and potential dermal toxicity	Xie et al. (2021)
	Not specified	Smoking and chewing tobacco	Twin cohort study	2b	113 male twins; Pack-years smoked	Moderate (strong design for observational, but cannot prove causation)	Against Parkinson’s	Twins without Parkinson’s smoked significantly more, suggesting smoking reduces Parkinson’s disease risk	Potential neuroprotective effects of tobacco constituents; not confounded by genetics or environment	Despite the observed inverse association between smoking and Parkinson’s disease risk, smoking can cause serious adverse health effects	Tanner et al. (2002)
	Leaves	Methanolic extract	Experimental animal study	2a	Mice/rats; 100, 200 and 300 mg/kg, orally	Moderate	Anti-nociceptive (central and peripheral), analgesic	Significant dose-dependent reduction in pain behaviors, analgesic effect confirmed	Analgesic effects likely involve activation of nicotinic acetylcholine receptors and modulation of the opioid system, which synergistically enhance pain inhibitory pathways and neurotransmitter release, resulting in dose-dependent pain relief	Sesquiterpene showed cytotoxic activity, nicotine caused tremors leading to convulsions at toxic doses	Ezeja and Omeh (2010)
*Passiflora edulis* Sims	Stems and leaves	Ethanol extract; Cycloartane triterpenoid saponins (2 new)	Animal behavioral assays	2a	Mice; 2 and 10 g/kg extracts orally	Moderate	Anti-depressant like activity	Extracts and isolated cyclopassiflosides showed significant antidepressant-like effects in forced swim and tail suspension tests	Cycloartane triterpenoid saponins exert antidepressant-like effects by modulating central monoamine neurotransmitters, enhancing brain-derived neurotrophic factor (BDNF) signaling, and normalizing hypothalamic-pituitary-adrenal (HPA) axis function	Study did not report specific toxicity findings	Wang et al. (2013)
	Leaves	Aqueous extract and fractions (ethyl acetate, butanol); Flavonoids	Animal behavioral assays	2a	Mice; 25–300 mg/kg orally	Moderate	Anti-depressant like activity	Antidepressant effects blocked by inhibitors of serotonin (5-HT) synthesis and dopamine D2 receptor antagonist; noradrenergic system not involved	Possibly flavonoids are promoting antidepressant-like effects through 5-HAT and dopamine transmission	No significant behavioral or hematological toxicity was reported with aqueous and ethanol extracts	Ayres et al. (2017)
	Leaves	Aqueous extract	Animal behavioral assays	2a	Male rats; 25, 50, 100, 150 mg/kg	Moderate	Anxiolytic	Extracts reduced anxiety-related behaviors in the elevated plus maze test without impairing memory; flavonoid content of *P. edulis* extract is twice that of *P. alata*	Flavonoid-rich aqueous extracts likely modulate GABA-A receptor activity, enhancing inhibitory neurotransmission to reduce anxiety without impairing memory	No adverse effects on memory or motor activity reported	Barbosa et al. (2008)
	Leaves	Aqueous extract and sub-fractions (chloroform, ethyl acetate, butanol); Flavonoids (enriched flavone-C-glycosides)	Animal behavioral assays	2a	Male mice; Various doses orally	Moderate	Anxiolytic activity	Butanol and chloroform fractions showed strongest anxiolytic effects and increased brain GABA levels; luteolin-7-O-[2-rhamnosylglucoside] isolated from flavonoid fraction showed anxiolytic-like activity without compromising motor activity	Synergistic modulation of GABAergic system likely via flavonoid C-glycosides	No significant adverse effects; good safety profile	Coleta et al. (2006)
	Arial parts (Leaves)	Ethanolic extracts and sub-fractions (petroleum ether, ethyl acetate, n-butanol, aqueous); Flavonoids including isoorientin	Animal behavioral assays	2a	Mice, EE 300–400 mg/kg; BE 125–300 mg/kg; isoorientin 20–80 mg/kg)	Moderate	Sedative, Anxiolytic	*P. edulis ‘flavicarpa’* (syn*. P.eduls* Sims) anxiolytic effects at high doses for ethanol extract and butanol extract; sedative effects at higher doses; isoorientin as important flavonoid component; motor activity impaired at sedative doses	Flavonoids, particularly isoorientin, contribute to GABAergic modulation inducing anxiolytic and sedative effects	Well tolerated at anxiolytic doses; sedative doses reduce motor activity, but no severe toxicity reported	Li et al. (2011)
	Leaves	Dried leaved: aqueous decoction	Animal behavioral assays	2a	Mice; Multiple doses orally (exact not specified)	Moderate	Anti-convulsive, Sedative	Reduced locomotor activity dose-dependently; exhibited sedative effects; significant anticonvulsant effects in seizure models by strychnine at 320 mg/kg	Modulation of inhibitory GABA-A and glycine receptors and excitatory NMDA receptors by flavonoids and alkaloids, enhancing CNS inhibition and stabilizing neuronal excitability for sedative and anticonvulsant effects	Well tolerated; no acute toxicity observed	Bum et al. (2004)
*Psidium guajava* L.	Fresh leaves	Ethanolic extracts	Animal behavioral assays	2a	Mcie; Oral doses (100, 200, 400 mg/kg)	Moderate (no randomization, Allocation/housing/blinding unclear)	Anxiolytic, Anti-depressant, memory enhancing	Improved learning, memory, and anxiolytic behaviors in mice; dose-dependent increase in brain neurotrophic factor (BDNF) levels	Enhancement of cholinergic and neurotrophic pathways; antioxidant effects contributing to neuroprotection	Well tolerated; no adverse effects reported	Biswas et al. (2021)
	Leaves	Hexane, ethyl acetate and methanol extracts	Animal behavioral assays	2a	Mice; Oral doses (20, 100, 500, 1,250 mg/kg)	Moderate (no randomization, Allocation/housing/blinding unclear)	Anticonvulsant, Sedative, CNS depressant	Dose-dependent CNS depression; decreased locomotor activity and exploratory behavior; anticonvulsant effects in PTZ-induced seizure model	extracts likely exert CNS depressant and anticonvulsant effects by enhancing GABAergic neurotransmission, increasing inhibitory signaling and reducing neuronal excitability	Well tolerated in tested dose range	Shaheen et al. (2000)
	Leaves	Aqueous extracts + ethanolic precipitated supernatant; Polyphenols	Chemical analysis (LC-HRMS-MS-Qtof); various bioassays	3	N/A	Low (high-quality analytical study)	Enzyme inhibition, Alzheimer’s disease	Good bioavailability suggested for several compounds, Aqueous extract showed high AChE inhibitory activity, AChe are important in the symptomatic treatment of Alzheimer disease	Polyphenols act as antioxidants and inflammation modulators	No safety concerns reported in this profiling study	Lorena et al. (2022)
*Psychotria viridis* Ruiz and Pav	leaves	Hexane, chloroform and methanol extracts	Chemical analysis and biological assays	3	N/A	Low	Potentially against Alzheimer’s	Hexane, and methanol extracts and the constituents cycloartenol, DMT, and a mixture of β-sitosterol and stigmasterol significantly inhibited AChE, chloroform and ethyl acetate induced inhibition above 90%	Hallucinogenic effects mainly due to DMT; other compounds have antioxidant and anti-inflammatory properties	non-cytotoxicity on normal cell strains, methanol extract showed cytotoxicity on tumor cells	Soares et al. (2017)
*Tabernae-montana heterophylla* Vahl	Not specified	Bulk dried plant parts were processed into crude extracts	Phytochemical analysis and pharmacological assays	3	N/A	Moderate (unclear replication and reporting)	Improve cognitive functioning	Contains high diversity of indole alkaloids, Extracts displayed activity at 6 5HT subtypes, 3 adrenergic subtypes, both opiate subtypes, the histamine H2 receptor, and the norepinephrine transporter (NET)	Alkaloids modulate cholinergic system by inhibiting acetylcholinesterase; anti-inflammatory actions via flavonoids and terpenes	Limited toxicity data; traditional use suggests tolerable safety	McKenna et al. (2011)
*Valeriana clematitis* Kunth	Whole plant (Liana)	Ethanolic extract and alkaloid fraction (aromatic and terpenoid groups, flavonoids, steroids)	Animal anticonvulsant assays	2a	Mice; dose not specified	Moderate	Anti-convulsive	Alkaloid fraction showed higher protection against seizures (70%) compared to the ethanolic extract (40%)	Anticonvulsant effects mainly by positive modulation of GABA-A receptors at the benzodiazepine site, enhancing inhibitory neurotransmission and reducing seizure activity. Key active compounds include hydrine-type valepotriates and isovaleramide	No acute toxicity reported in tested models	Arévalo et al. (2006)
	Whole plant (Liana)	Ethanolic extract and alkaloid fraction	Animal behavioral assays	2a	Mice, dose not specified	Moderate	Anti-convulsive, Anti-depressant, Sedative	Anticonvulsant and antidepressant-like effects are higher in alkaloid fraction compared to ethanolic extract, ethanolic extract showed sedative effect	Positive allosteric modulation of GABA-A receptors at benzodiazepine site by valepotriates and isovaleramide; sedative effects from flavonoids/terpenoids	No acute toxicity reported	Celis et al. (2007)
	whole plant (Liana)	Alkaloid fraction + isolated iso-valeramide	Animal anticonvulsant assays	2a	Mice; 100 mg/kg oral	Moderate	Anti-convulsive	Isovaleramide provided 90% protection against maximal electroshock seizures in mice, comparable to phenytoin (20 mg/kg); inhibited benzodiazepine binding site *in vitro*	Positive modulation at GABA-A receptor benzodiazepine site demonstrated via binding assays; likely enhances inhibitory neurotransmission	No acute toxicity reported	Giraldo et al. (2010)

N/A: not applicable.

*Levels of evidence for conducted studies (WHO, 2019): 1b: Individual randomized controlled trials with narrow confidence intervals; 2a: Controlled cohort studies or well-designed experimental animal studies demonstrating mechanism; 2b: Open-label, observational, or non-randomized clinical studies; 3: Non-controlled experimental studies, case series, or mechanistic studies with some biological plausibility; 4: *In vitro* or preclinical mechanistic studies without direct clinical evidence.

**Risk-of-Bias analysis: For clinical studies, the updated Cochrane Risk-of-Bias tool 2.0 (RoB 2) (Sterne et al., 2019) was applied to all randomized controlled trials, focusing on randomization process, blinding, deviations from intended interventions, missing outcome data, measurement of outcomes, and selection of reported results. For preclinical *in vivo* studies, the SYRCLE, risk-of-bias tool (Hooijmans et al., 2014) was utilized, considering baseline characteristics, allocation concealment, housing, blinding, outcome assessment, incomplete data, and selective reporting.

### 3.2 Ethnobotanical and pharmacological overview of Colombian plants used for neuropsychiatric conditions

The ethnobotanical literature documents the use of medicinal and sacred plants across a wide range of Colombian geographic regions and sociocultural contexts. Reported uses span urban settings, such as traditional markets in Bogotá, as well as rural and Indigenous communities throughout the Amazon, Orinoquía (Llanos), Andean regions, and other biogeographic areas. The included sources represent a diversity of Indigenous Peoples and local communities, including—among others—Ticuna, Siona, Yukuna, Witoto, Tanimuka, Cubeo, Cofán, Kamsá, and Coreguaje in Amazonian regions; Sikuani and Cuiba in the Llanos; and additional Indigenous and campesino communities in Andean municipalities (e.g., Boyacá, Cundinamarca, Antioquia) and in Huila.

Across the 62 ethnobotanical sources included in this review, more than half of the documented species (28 of 42) were reported in two or more independent studies (Supplementary Table S1), indicating recurrent documentation and broad ethnobotanical recognition. The temporal coverage of the literature spans both historical accounts—particularly early to mid-20th-century ethnobotanical surveys—and contemporary studies, reflecting the long-standing continuity of traditional knowledge as well as its ongoing documentation in modern research contexts.

### 3.3 Diversity of species

**FIGURE 3:**
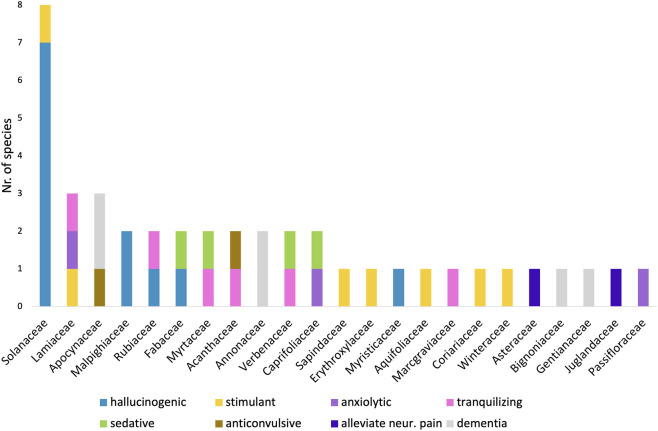
Distribution of the 42 identified Colombian species across plant families and their CNS-related activities. Solanaceae dominates, largely due to its hallucinogenic species, followed by Lamiaceae and Apocynaceae.

The 42 plant species documented in this review represent 23 botanical families. Solanaceae contributed the highest number of species, while Lamiaceae and Apocynaceae each contributed three species (Figure 3). At the genus level, *Brugmansia* was represented by four species, whereas *Unonopsis*, *Psychotria*, and *Valeriana* were each represented by two species.

**FIGURE 4:**
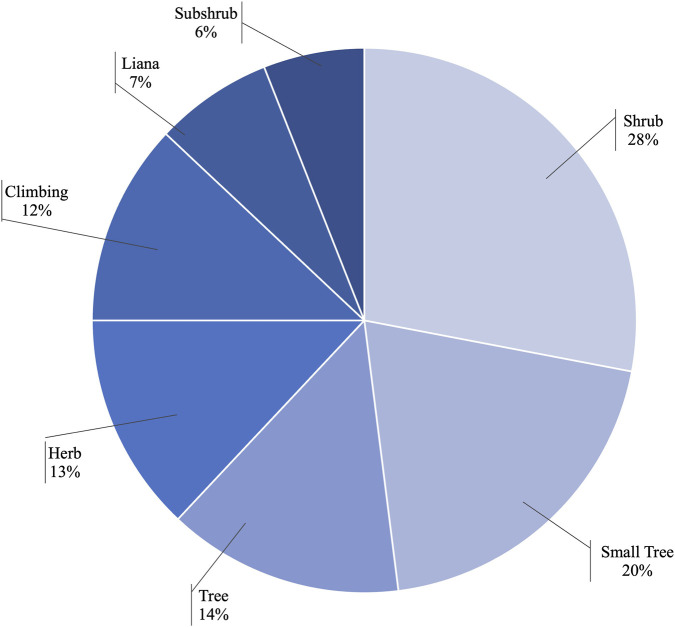
Growth habits of the covered species.

Documented growth forms include shrubs, small trees, trees, and herbs (Figure 4), with several taxa reported under more than one growth habit category (e.g., herb–shrub). Shrubs constituted the most common growth form (approximately 28% of species, n = 19), followed by small trees (20%, n = 14), trees (14%, n = 10), and herbs (13%, n = 9). The majority of species (approximately 90%, n = 37) are native to Colombia, although none are endemic. Twelve species were reported as cultivated and four as naturalized; some taxa occur in more than one of these categories (Figure 4).

Distinct patterns of traditional use emerged at the family level. Species in Apocynaceae and Annonaceae were predominantly associated with treatments for age-related cognitive or neurological conditions, whereas all documented Malpighiaceae species exhibited hallucinogenic properties. Conversely, all Annonaceae species identified in this review were traditionally employed for age-related disorders.

To assess whether particular plant families were overrepresented among CNS-relevant taxa, the family composition of the 42 species was compared with published estimates from Bystriakova et al. (2021) of family representation in the Colombian vascular flora. Solanaceae accounted for eight species (19%) in the review dataset, substantially exceeding its reported contribution to the national flora (approximately 3%). Similarly, Apocynaceae and Lamiaceae each accounted for three species (7%), exceeding their estimated representation in the Colombian flora (approximately 2% and 1.5%, respectively). In contrast, species-rich families in Colombia, such as Asteraceae and Fabaceae, contributed relatively few species with documented CNS-related traditional uses in this dataset (Figure 3).

### 3.4 Identified species by CNS effect category and traditional use

All 42 plant species included in this review were classified according to their primary CNS–related effect category, based on ethnobotanical documentation and the hybrid analytical framework described in Section 2.5. Traditional uses reported in ethnobotanical sources were mapped to eight biomedical CNS–related effect categories for comparative purposes. Species within each category are presented according to ethnobotanical prominence and geographic distribution within Colombia. Vernacular names were compiled from primary ethnobotanical sources and cross-referenced with the annotated checklist of useful plants of Colombia (Diazgranados, et al., 2022). An overview of species grouped by CNS–related effect category, together with vernacular names, is provided in Supplementary Table S3, while detailed ethnobotanical records—including plant parts used, preparation methods, and traditional indications—are summarized in Supplementary Table S1.

Although several species exhibit trans-regional distribution, traditional uses vary across Colombia’s diverse ecosystems and cultural contexts. Most records indicate long-standing, multi-generational use across multiple regions and ethnic groups.

#### 3.4.1 Hallucinogenic species

Twelve species were classified as hallucinogenic based on consistent ethnobotanical documentation. These species are predominantly associated with Indigenous communities of the Amazon and Andean regions. The most widely documented plants used in the hallucinogenic preparation of the psychoactive beverage yagé (ayahuasca), notably *Banisteriopsis caapi* (Spruce ex Griseb.) Morton, *Psychotria viridis* Ruiz and Pav., and *Diplopterys cabrerana* (Cuatrec.) B. Gates. These species are reported among multiple Indigenous groups in the Putumayo, Vaupés, Caquetá, and Guaviare regions, including Siona, Cofán, Inga, Secoya, Witoto, Bora, Miraña, Tukanoan groups (e.g., Desana, Barasana, Cubeo), Tikuna, and Coreguaje.

Additional hallucinogenic taxa include species of *Virola* (Myristicaceae), particularly *Virola calophylla* Warb., which are traditionally prepared as snuff by Indigenous communities such as the Witoto, Bora, Ocaina, Makuna, Cubeo, and Tukanoan groups. In the Andes, Amazon, and Orinoquía regions, several hallucinogenic species, collectively known by vernacular names such as *borrachero,* belong to the genus *Brugmansia* (Solanaceae). Four species are particularly well documented in Colombia: *Brugmansia* × *candida* Pers., *B. aurea* Lagerh., *B. arborea* (L.) Sweet, and *B. sanguinea* (Ruiz and Pav.) D. Don. Other hallucinogenic species reported primarily from Andean regions include *Brunfelsia grandiflora* D. Don, *Datura stramonium* L., *Anadenanthera peregrina* (L.) Speg., and *Iochroma fuchsioides* (Bonpl.) Miers.

#### 3.4.2 Stimulant species

Seven species were identified as stimulants, often used in contexts of physical collapse, fatigue, or ritual activity. These plants frequently hold nutritional, ceremonial, and social significance. The most prominently documented stimulant species are *Erythroxylum coca* L., widely used among Amazonian Indigenous groups, such as the Cofán, Inga, Coreguaje, Kamsá, Kubeo, Barasana, and Makuna, and *Nicotiana tabacum* L., which is reported across diverse regions and communities, including Andean rural populations and multiple Amazonian Indigenous groups. The stimulant effects of *N. tabacum* vary according to preparation method and route of administration and are subject to ethnobotanical debate.

Other stimulant species include *Ilex guayusa* Loes. (used by Quichua, Siona, and Secoya), *Paullinia yoco* R.E. Schult. and Killip (used by Inga, Siona, and Cofán), as well as *Coriaria ruscifolia* (Mart.) de Roon, *Drimys granadensis* L.f., and *Lepechinia bullata* (Kunth) Epling, which are primarily reported from rural communities in high-Andean and páramo ecosystems of Cundinamarca and Boyacá.

#### 3.4.3 Tranquilizing species

Six species were consistently described as having calming or tranquilizing properties, including uses for nervous conditions, insomnia, and anxiety-related states. *Psidium guajava* L. and *Aloysia citrodora* Palau are widely used throughout Colombia. Reported uses of *P. guajava* originate mainly from Andean rural households (Boyacá, Tolima) and Caribbean coastal regions, whereas *A. citrodora* is frequently documented in Antioquia, Boyacá, and urban markets in Bogotá.

Other tranquilizing species include *Ocimum campechianum* Mill., *Souroubea corallina* (Mart.) de Roon (reported among the Pijao), *Psychotria guianensis* (Aubl.) Clos (reported from the Orinoquía region), and *Dianthera pectoralis* (Jacq.) J.F. Gmel., which exhibits one of the widest geographic and cultural distributions, spanning Andean rural communities, urban markets, Orinoquía Indigenous groups (Sikuani, Cuiba), and numerous Amazonian Indigenous Peoples.

#### 3.4.4 Anticonvulsant species

Two species were reported as used for convulsions. Species used for conditions described as “trembling” include *Galactophora crassifolia* (Müll. Arg.) Woodson and *Justicia idiogenes* Leonard.

#### 3.4.5 Age-related neurological symptoms

Traditional treatments for epilepsy, convulsions, and age-related cognitive or neurological conditions were frequently documented as overlapping categories. Six species were reported for age-related disorders, particularly in ethnobotanical accounts by Schultes. Species associated with age-related mental disorders include *Adenocalymma schomburgkii* (DC.) L.G. Lohmann, *Irlbachia nemorosa* (Willd. ex Schult.) Merr., *Mandevilla steyermarkii* Woodson, *Unonopsis veneficiorum* (Mart.) R.E. Fr., *Unonopsis stipitata* Diels, and *Tabernaemontana heterophylla* Vahl. These species are primarily reported from Indigenous groups of the northwestern Amazon, including Ticuna, Siona, Yukuna, Tanimuka, Cubeo, and Cofán.

#### 3.4.6 Sedative species

Four species were classified as sedatives and are mainly reported from rural Andean communities with limited access to biomedical healthcare. *Lippia alba* (Mill.) N.E. Br. ex Britton and P. Wilson and *Mimosa albida* Humb. and Bonpl. ex Willd. are widely recognized medicinal plants with reported uses across South and Central America. In Colombia, *L. alba* is reported from Boyacá, Tolima (Pijao communities), and Caribbean Afro-Colombian populations, while *M. albida* was reported only once from rural Antioquia. The remaining sedative species, *Myrcianthes leucoxyla* (Ortega) McVaugh and *Valeriana scandens* L., are native and reported from Andean rural communities in Boyacá, Cundinamarca, and Antioquia.

#### 3.4.7 Against neurological pain

Two tree species, *Smallanthus pyramidalis* (Triana) H. Rob. and *Juglans neotropica* Diels, were documented for the treatment of neuropathic pain. These species are primarily used by rural and farming communities in Antioquia and Cundinamarca, particularly in páramo and montane ecosystems between 1,600 and 3,000 m a.s.l. In addition to medicinal applications, both species are used for food, material, and environmental purposes.

#### 3.4.8 Anxiolytic species

Three species were classified as anxiolytic. *Passiflora edulis* Sims and *Valeriana clematitis* Kunth are included in the Colombian Vademecum of Medicinal Plants (Ministerio de Protección Social, 2008), reflecting their widespread and institutionalized medicinal use. In Colombia, P. edulis is reported from rural communities in Antioquia and among Pijao Indigenous communities, while *Hyptis brachiata* Briq. is more regionally used in Colombia, Central America, and Venezuela, and is reported from Indigenous groups in the Orinoquía region.

### 3.5 Utilized plant parts and traditional medicinal applications

Information on plant parts used and modes of medicinal preparation was compiled for all 42 species included in the review. Detailed ethnobotanical records—including vernacular names, plant parts utilized, preparation methods, and principal traditional indications—are provided in Supplementary Table S1. For all species, at least one specific plant part associated with medicinal use was documented, with the exception of *O. campechianum*, for which the whole plant was reported to be used.

**FIGURE 5:**
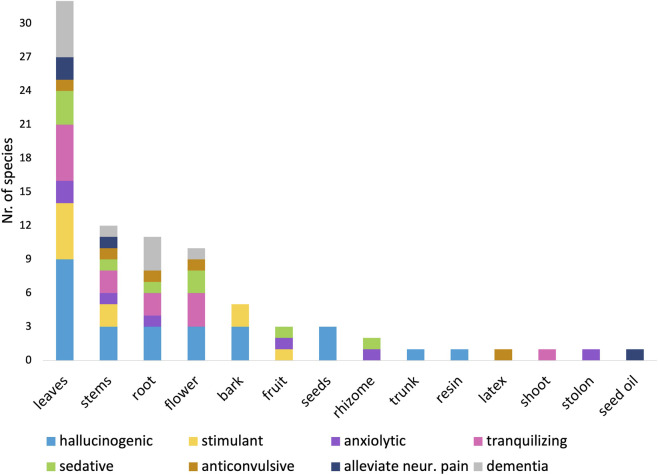
Most frequently used plant parts in traditional CNS remedies. Leaves and stems are most cited, reflecting both accessibility and pharmacological relevance in decoctions and infusions.

Across all species, a total of 14 distinct plant parts were recorded as being used for medicinal purposes (Figure 5). Leaves were the most frequently utilized plant part (32 species), followed by stems (12 species), roots (11 species), and flowers (10 species). Other plant parts—including bark, trunk, latex, shoots, stolons, and seed oil—were each reported for only a single species.

Leaves and stems were associated with medicinal uses spanning all eight CNS-related effect categories identified in this review. Roots were used in seven of the eight categories, with the exception of treatments for neuropathic pain. Bark was reported exclusively in the context of hallucinogenic and stimulant preparations. Five species—*M. albida*, *I. nemorosa*, *J. idiogenes*, *D. pectoralis*, and *A. citrodora*—were documented as utilizing four different plant parts for medicinal purposes, reflecting a high degree of versatility in traditional applications.

A total of 18 distinct preparation methods were recorded, including decoctions, infusions (herbal teas), tinctures, oils, syrups, juices or beverages, powders or snuffs, and pellets or pills. These preparations correspond to five main routes of administration: oral ingestion, external or topical application, inhalation (e.g., snuff or smoke), and other routes such as enemas or chewing.

**FIGURE 6:**
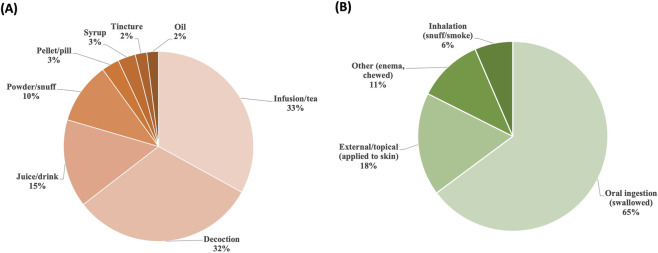
**(A)** Number of indicated traditional medicinal applications. **(B)** Ways of consumption relative to the total number of reported medicinal applications and ways of consumption respectively. Species can have multiple applications and consumption methods.

Infusions (herbal teas) were the most commonly reported preparation method, documented for approximately one-third of species (14 species, ∼33%), followed closely by decoctions (13 species, ∼32%) (Figure 6A). Preparations involving fresh juices or beverages were also frequently reported, whereas oils and tinctures were rare, each documented for only a single species. In ethnobotanical contexts, decoctions typically involve boiling tougher plant parts such as roots or bark, whereas infusions are prepared by steeping more delicate tissues, particularly leaves and flowers. Juice or beverage preparations involve pressing or blending fresh plant material to extract liquid.

*Nicotiana tabacum* exhibited the greatest diversity of preparation methods, with seven distinct medicinal applications reported. *Psidium guajava* followed with six preparation types, and *D. pectoralis* with five. Across all species, oral ingestion was the predominant route of administration (27 species, ∼65%), followed by external or topical application (8 species, ∼18%) and inhalation (3 species, ∼6%) (Figure 6B). Oral ingestion encompassed a broad range of practices, including consumption of raw plant material, powders, extracts, infusions, decoctions, and beverages. External applications included topical use of oils or tinctures, as well as the application of fresh or moistened plant material (e.g., poultices or cataplasms).

### 3.6 Species with phytochemical and pharmacological studies on beneficial CNS-related activities

#### 3.6.1 Availability and depth of phytochemical characterization

Among the 42 plant species included in this review, phytochemical information was available for 37 species, while five species—*V. scandens*, *J. idiogenes*, *I. nemorosa*, *A. schomburgkii*, and *S. corallina*—lacked published analyses of secondary metabolites (Table 3). Given their documented traditional uses for CNS-related conditions, these taxa represent important targets for future phytochemical investigation.

The depth of phytochemical characterization varied substantially across taxa. Eighteen species were extensively studied, with ten or more publications addressing their metabolite profiles, whereas eleven species were minimally investigated, with fewer than five phytochemical studies each. Five species had no phytochemical studies identified. *Nicotiana tabacum* was the most comprehensively characterized species, reflecting its long-standing relevance in alkaloid research and neuroactive compound discovery.

#### 3.6.2 Neuropharmacological validation across species

Neuropharmacological evidence—including *in vitro* assays, *in vivo* animal models, and human clinical studies—was reviewed for all species with available phytochemical data. Only studies in which extracts or metabolites were directly derived from the identified plant species were included.

Overall, experimental validation of CNS-related activity was limited. Fourteen species demonstrated pharmacological effects relevant to CNS function, whereas 28 species lacked any neuropharmacological studies meeting inclusion criteria (Table 4). The species with validated activity were: *A. citrodora*, *B. caapi*, *Brugmansia arborea*, *D. stramonium*, *D. pectoralis*, *D. cabrerana*, *E. coca*, *L. alba*, *N. tabacum*, *P. edulis*, *P. guajava*, *P. viridis*, *T. heterophylla*, and *V. clematitis*.

Reported extracts or isolated metabolites from these taxa were classified into 15 CNS-related biomedical effect categories, as described in the Methods section. A detailed synthesis of study design, compounds tested, outcomes, primary pharmacological effects, risk-of-bias assessment, and evidence levels, is provided in Table 4.

#### 3.6.3 Distribution of reported CNS effects

Among species with pharmacological validation, hallucinogenic activity was most frequently reported (12 species), followed by anxiolytic effects (10 species), antidepressant activity (8 species), and analgesic effects (7 species). Several taxa exhibited activity across multiple CNS categories, and those with the broadest range of reported effects were also the most extensively studied.

Species used in the preparation of the hallucinogenic beverage ayahuasca (yagé) collectively accounted for nine distinct CNS-related pharmacological effects. However, experimental studies focused almost exclusively on three taxa—*B. caapi*, *D. cabrerana*, and *P. viridis*—despite ethnobotanical documentation indicating that more than 100 plant species may be incorporated into ayahuasca preparations across different cultural contexts (Trujillo et al., 2010), sometimes including *Brugmansia* species or *N. tabacum*.

#### 3.6.4 Clinical evidence and translational gaps

Clinical evidence remains sparse. Only five species—*B. caapi*, *P. viridis*, *D. cabrerana*, *A. citrodora*, and *D. stramonium*—were evaluated in randomized and/or placebo-controlled clinical studies. Among these, *A. citrodora* was the most extensively investigated, with multiple trials demonstrating anxiolytic and sleep-promoting effects (Martínez-Rodríguez et al., 2022).

Several clinical studies also assessed ayahuasca preparations, typically combining *B. caapi* with *P. viridis* or *D. cabrerana*. A comprehensive synthesis of ayahuasca clinical trials has been published elsewhere (Dos Santos et al., 2016).

For some widely used species, clinical evidence remains absent or inconclusive. *Passiflora edulis* has been evaluated in clinical settings, but not for CNS-related outcomes; moreover, persistent taxonomic confusion with *Passiflora incarnata* has contributed to inconsistent reporting. *Erythroxylum coca* has been extensively studied chemically—particularly with respect to cocaine—but no studies were identified that assessed CNS effects of whole-plant extracts, and little is known about the biological activity of other coca alkaloids. For most remaining taxa, research has not progressed beyond *in vitro* assays or preclinical animal models, often employing heterogeneous methodologies.

#### 3.6.5 Risk-of-bias and methodological quality

Risk-of-bias assessment revealed variable methodological quality across the neuropharmacological literature (Table 4). Among eight randomized controlled trials, seven were rated as low risk of bias, reflecting adequate randomization, double-blinding, and outcome reporting. These included trials on ayahuasca (Palhano-Fontes et al., 2019; Sanches et al., 2016), *A. citrodora* for anxiety and insomnia (Afrasiabian et al., 2019; Martínez-Rodríguez et al., 2022; Alvarado-García et al., 2021), and a combination study including *D. stramonium* for opioid dependence (Moosavyzadeh et al., 2020). One randomized control trial (RCT) evaluating *B. caapi* for Parkinson’s disease (Serrano-Dueñas et al., 2001) was rated as moderate risk due to limited methodological detail, small sample size, and short observation periods.

Common limitations across clinical trials included small sample sizes, insufficient reporting of extract composition and dosing, unclear placebo definitions, and absence of intention-to-treat analyses. Observational clinical studies (n = 10) were consistently rated as moderate risk of bias due to non-randomized designs, absence of placebo controls, and potential confounding by contextual or ritual factors.

All preclinical animal studies (n = 32) were rated as moderate risk of bias, primarily due to incomplete reporting of randomization, blinding, allocation concealment, and sample size justification rather than evidence of flawed experimental design. Sample sizes were typically small (n = 6–12 per group), and no studies reported preregistered protocols or power calculations. Nevertheless, many studies demonstrated internal validity through dose–response analyses, use of appropriate pharmacological controls (e.g., diazepam, flumazenil), receptor-binding assays, neurochemical analyses, and replication of traditional preparation methods. *In vitro* and chemical studies (n = 6) were generally well reported and methodologically robust.

### 3.7 Mechanistic insights: Phytochemistry and neuropharmacological targets

The phytochemical composition and neuropharmacological mechanisms of the 42 Colombian medicinal plant species are shown in Supplementary Table S3. To link traditional ethnobotanical knowledge with contemporary neuropharmacology, we synthesized available evidence connecting phytochemical constituents of Colombian medicinal plants to specific central nervous system (CNS) targets and mechanisms of action (Supplementary Table S2). This synthesis reveals a wide range of molecular pathways through which these species may exert neuropsychiatric effects. Mechanistic evidence has been experimentally verified for 14 species through preclinical or clinical studies, whereas for the remaining taxa proposed mechanisms remain hypothetical and require targeted investigation. All reported mechanisms are verified if not stated otherwise.

#### 3.7.1 Serotonergic modulation

The ayahuasca (*yagé*) preparation exemplifies a well-characterized serotonergic mechanism. It combines β-carboline alkaloids—harmine, harmaline, and tetrahydroharmine—from *B. caapi*, which act as reversible monoamine oxidase-A (MAO-A) inhibitors, with N,N-dimethyltryptamine (DMT) from *P. viridis* or *D. cabrerana*, a potent serotonin 5-HT_2_A receptor agonist (Dos Santos et al., 2016). Inhibition of MAO-A prevents the rapid metabolic degradation of DMT, enabling its central activity at 5-HT_2_A receptors (Shen et al., 2010). This synergistic interaction produces hallucinogenic, antidepressant, and anxiolytic effects mediated by enhanced serotonergic neurotransmission and downstream neuroplasticity (Vargas et al., 2023). Clinical trials have confirmed antidepressant and anxiolytic effects in treatment-resistant populations (Palhano-Fontes et al., 2019).

#### 3.7.2 GABAergic enhancement

Several species exhibit anxiolytic, sedative, and anticonvulsant properties mediated in part by modulation of γ-aminobutyric acid type A (GABA_A) receptors and monoaminergic signaling pathways (Sabti et al., 2019). GABA_A receptors regulate neuronal excitability via inhibitory neurotransmission. Dysfunction of GABAergic signaling is implicated in anxiety disorders, epilepsy, mood disorders, schizophrenia, and other neuropsychiatric conditions (Aragão et al., 2006).

*Lippia alba* contains monoterpenes such as citral, carvone, and linalool that enhance GABAergic transmission, producing sedative and anxiolytic effects validated in clinical trials for migraine relief (Carmona et al., 2013). Alkaloids from *T. heterophylla*, particularly coronaridine, are parteally verified to increase GABA_A receptor affinity *in vitro* and elicit anxiolytic-like effects in murine models (Arias et al., 2023). Extracts of *D. pectoralis* show high affinity in GABA bioassays and demonstrate anxiolytic and sedative effects *in vivo*, supporting their traditional use (Awad et al., 2009; Venâncio et al., 2011). In addition, phytol derived from *B. grandiflora* exhibits anticonvulsant activity mediated by GABAergic mechanisms (Costa et al., 2012).

#### 3.7.3 Cholinergic modulation

Plants traditionally used for age-related cognitive decline frequently exhibit acetylcholinesterase (AChE) inhibitory activity, a mechanism relevant to symptomatic treatment of Alzheimer’s disease. Leaf extracts of *P. guajava*, rich in quercetin and gallic acid, inhibit AChE *in vitro* (Lorena et al., 2022). Alkaloids isolated from *T. heterophylla*, including voacangine and coronaridine, show AChE inhibitory activity comparable to related *Tabernaemontana* species (Andrade et al., 2005; McKenna et al., 2011), suggesting potential cognitive-enhancing effects.

#### 3.7.4 Dopaminergic and adrenergic activation

Stimulant species primarily act by enhancing catecholaminergic neurotransmission. *Erythroxylum coca* contains the alkaloid cocaine, which inhibits dopamine, serotonin, and norepinephrine transporters, increasing synaptic monoamine concentrations and producing well-documented stimulant effects. *Nicotiana tabacum* contains nicotine, an agonist of nicotinic acetylcholine receptors (α4β2 and α7 subtypes), which indirectly stimulates dopamine and norepinephrine release, resulting in stimulant, depressant and cognitive-enhancing effects.

#### 3.7.5 Anticholinergic (muscarinic) antagonism

Tropane alkaloid-containing Solanaceae, including *Brugmansia* spp. and *D. stramonium*, exert CNS effects through antagonism of muscarinic acetylcholine receptors (M1–M5). Compounds such as scopolamine and hyoscyamine competitively block central and peripheral muscarinic receptors, producing delirium, amnesia, and hallucinations. Although these plants have hallucinogetic propertiesand are used in obstetric contexts, their narrow therapeutic window and high toxicity substantially limit safe pharmacological application.

#### 3.7.6 Neuroprotection and neuroplasticity

Several species demonstrate neuroprotective properties through antioxidant activity and promotion of neurite outgrowth. Ayahuasca-associated compounds have been shown to promote neuroplasticity and neuronal regeneration in experimental systems (Morales-García et al., 2017). Polyphenols from *P. guajava* activate the nuclear factor erythroid 2–related factor 2 (Nrf2) pathway, reducing oxidative stress and supporting cellular defense mechanisms relevant to neurodegeneration (Lorena et al., 2022). *Iochroma fuchsioides* contains withanolides (Raffauf et al., 1991), whose mechanism of action is hypothesized. In other studies, they have been shown to promote neurite regeneration and dendritic formation in neuroblastoma cell models, with potential relevance to Parkinson’s disease (Zhao et al., 2002; Hannan et al., 2020). Phenolic extracts from *B. grandiflora* also demonstrate cytoprotective effects against oxidative stress in neuron-like cells (Rodríguez et al., 2023).

#### 3.7.7 Monoaminergic modulation

Aqueous extracts of *D. pectoralis* significantly reduce dopamine, noradrenaline, serotonin, and their metabolites (DOPAC, HVA) in the prefrontal cortex and striatum of rodents (Venâncio et al., 2019). This monoaminergic dampening effect suggests potential utility in neuropsychiatric disorders characterized by monoaminergic hyperactivity, such as anxiety states, agitation, or affective dysregulation.

It is essential to distinguish between experimentally validated and hypothesized mechanisms. For species supported by clinical trials, mechanisms have been confirmed in humans. For species with preclinical evidence, mechanisms are supported by *in vitro* assays or animal models but lack clinical validation. For taxa with only phytochemical characterization, mechanisms remain speculative and are inferred from known activities of isolated compounds or related species. Interpretation is further constrained by the frequent testing of isolated compounds rather than whole extracts, differences between traditional preparation methods and laboratory extraction protocols, and limited pharmacokinetic data on bioavailability and blood–brain barrier penetration. Dose–response relationships and therapeutic windows remain poorly characterized for most species, underscoring the need for integrative pharmacological and translational research.

### 3.8 Overview of six priority species

Six priority species were identified as candidates for deeper translational research: *I. fuchsioides*, *B. grandiflora*, *S. corallina*, *T. heterophylla*, *P. guajava*, and *D. pectoralis*. The strength of evidence varies substantially across these taxa, and none has yet been investigated through a sufficiently comprehensive pipeline spanning phytochemistry, pharmacology, safety, and well-powered clinical evaluation. Among the six, *D. pectoralis* has the most robust preclinical evidence base, supported by four independent animal studies reporting consistent anxiolytic, sedative, and monoaminergic effects (Awad et al., 2009; Venâncio et al., 2011; 2019; Rodríguez-Chanfrau et al., 2013), although overall study quality was rated as moderate risk of bias. *P. guajava* is supported by three studies, including one high-quality chemical analysis (Lorena et al., 2022, low risk) and two animal behavioral studies (Shaheen et al., 2000; Biswas et al., 2021; both moderate risk). *B. grandiflora* has limited but suggestive *in vivo* evidence for anticonvulsant effects of phytol (Costa et al., 2012, moderate risk). *T. heterophylla* has preliminary receptor screening evidence (McKenna et al., 2011, moderate risk) but lacks focused *in vivo* validation. By contrast, *I. fuchsioides* and *S. corallina* have no identified neuropharmacological studies meeting the inclusion criteria, and their prioritization rests on phytochemical information, ethnobotanical indications, and mechanistic inference from related species. Together, these patterns support targeted mechanistic research focused on taxa with strong ethnobotanical support but limited pharmacological validation.

#### 3.8.1 Iochroma fuchsioides

Phytochemical studies of *I. fuchsioides* report withanolides, including withanolide D (Raffauf et al., 1991). Although direct neuropharmacological studies of *I. fuchsioides* are lacking, withanolides from *Withania somnifera* (notably withanolide A and D) have shown *in silico* evidence relevant to Alzheimer-associated pathobiology, including predicted effects on β-amyloid aggregation, tau hyperphosphorylation, and neuroinflammatory signaling (Hannan et al., 2020). *In vitro* studies also report neurite regeneration and dendritic formation in human neuroblastoma cell lines (Zhao et al., 2002), potentially involving BDNF and PI3K/Akt signaling pathways, with possible relevance to Parkinson’s disease. These findings motivate direct experimental validation in *I. fuchsioides*, alongside systematic safety and toxicity profiling.

#### 3.8.2 Souroubea corallina

No conclusive neuropharmacological studies were identified for *S. corallina*, despite ethnobotanical indications consistent with anxiolytic and potentially sedative-hypnotic use. Evidence from congeneric species suggests betulinic acid as a candidate anxiolytic constituent. In animal models, extracts from *Souroubea* spp. demonstrate anxiolytic activity (elevated plus maze and marble burying tests), partially blocked by flumazenil, supporting a GABA_A-related mechanism (Puniani et al., 2015). While these findings are not yet specific to *S. corallina*, they provide a mechanistic rationale for prioritizing phytochemical confirmation and neuropharmacological testing of this species.

#### 3.8.3 Brunfelsia grandiflora

Phytochemical analyses of *B. grandiflora* report scopoletin, phytol, and mannitol among major constituents. Leaf phenolic extracts exhibit cytoprotective and antioxidant effects against oxidative stress in neuron-like cell models (SH-SY5Y), suggesting potential relevance to neurodegenerative processes (Rodríguez et al., 2023). *In vivo*, phytol has demonstrated anticonvulsant activity in a pilocarpine-induced seizure model, reducing seizure severity, delaying seizure onset, and decreasing mortality, consistent with modulation of GABAergic neurotransmission (Costa et al., 2012). Although mechanistic resolution remains incomplete, these results support further work on whole-extract versus isolated-compound activity, dose–response characterization, and rigorous acute and subchronic toxicity assessment, particularly given toxicity concerns associated with some Solanaceae-derived preparations.

#### 3.8.4 Tabernaemontana heterophylla

*Tabernaemontana heterophylla* emerges as a high-priority taxon based on strong traditional indications for age-related cognitive disorders and preliminary pharmacological signals. A receptor-screening study reported activity at several CNS-relevant receptor targets, suggesting potential utility for cognitive deficits associated with schizophrenia or dementia (McKenna et al., 2011). Multiple alkaloids have been reported from this species—including voacangine, coronaridine, 19-heyneanine, vobasine, affinisine, and olivacine (Wolter Filho et al., 1983)—and coronaridine congeners have been associated with sedative, anxiolytic-like, and anticonvulsant activities via increased GABA_A receptor affinity *in vivo* (Arias et al., 2023). Evidence from related *Tabernaemontana* species further supports CNS relevance: *Tabernaemontana arborea* exhibits antidepressant and antinociceptive activity in murine models without neurotoxicity (Gonzalez-Trujano et al., 2023), and alkaloid extracts from *Tabernaemontana australis* show AChE inhibitory activity (Andrade et al., 2005; Wolter Filho et al., 1983). Given these convergent signals, *T. heterophylla* warrants focused *in vivo* validation, AChE-targeted assays for cognitive indications, and standardized extract profiling; however, clinical validation is currently absent.

#### 3.8.5 Psidium guajava

Preclinical studies support multiple CNS-relevant effects of *P. guajava*. Ethanolic extracts of leaves and fruit produced antidepressant- and anxiolytic-like effects in mice in a dose-dependent manner (forced swim test; elevated plus maze) (Biswas et al., 2021). Additional work reported sedative and antinociceptive effects across solvent extracts (Shaheen et al., 2000), though mechanistic attribution (e.g., GABAergic versus opioidergic mediation) remains unresolved. Mechanistically, aqueous leaf extracts show AChE inhibitory activity linked to phenolic constituents including quercetin and gallic acid (Lorena et al., 2022), consistent with a plausible symptomatic pathway for Alzheimer’s disease (Breijyeh and Karaman, 2020). Together with broader evidence of antioxidant and anti-inflammatory activity (Biswas et al., 2021), these findings highlight a translational gap: despite a relatively strong preclinical basis and wide availability, clinical trials targeting CNS indications have not been identified.

#### 3.8.6 Dianthera pectoralis

*D. pectoralis* represents the most promising of the six taxa based on convergent preclinical evidence, ethnobotanical indications, and mechanistic plausibility, although no clinical trials have been identified. Aqueous extracts of aerial parts produced anxiolytic-like effects in mice using the elevated plus maze (Venâncio et al., 2011), with dose-dependent responses comparable to standard anxiolytics. Ethanolic extracts showed high affinity in GABA bioassays, consistent with anxiolytic and anticonvulsant potential (Awad et al., 2009). *In vivo*, aqueous extracts also demonstrated sedative and anti-aggressive effects (Rodríguez-Chanfrau et al., 2013). Importantly, mechanistic work suggests an additional pathway: aqueous extracts significantly reduced dopamine, noradrenaline, serotonin, and key metabolites (DOPAC, HVA) in the prefrontal cortex and striatum of rats (Venâncio et al., 2019). This monoaminergic dampening profile distinguishes *D. pectoralis* from purely GABAergic sedatives and may be relevant to psychiatric conditions characterized by hyperarousal or dysregulated monoaminergic signaling. These findings support prioritization of standardized extract characterization, dose–response and safety studies, and subsequent early-phase clinical testing.

## 4 Discussion

This review synthesized ethnobotanical, phytochemical, pharmacological, and limited clinical evidence on Colombian medicinal plants traditionally used for conditions affecting the central nervous system (CNS). A total of 42 species were documented from Indigenous, Afro-Colombian, and campesino health systems, highlighting Colombia’s exceptionally rich yet underexplored ethnopharmacological landscape. Despite Colombia’s high biodiversity and long tradition of medicinal plant use, scientific validation of CNS-related applications remains fragmentary, with substantial gaps between traditional knowledge and pharmacological or clinical research.

Although the number of publications on medicinal plants in Latin America has increased in recent decades, Colombia remains comparatively underrepresented in the scientific literature. Recent bibliometric analyses indicate that Colombia ranks sixth among Latin American countries in medicinal plant research output, following Brazil, Mexico, Argentina, Chile, and Cuba (Alarcón-Ruiz et al., 2023). Moreover, a substantial proportion of ethnobotanical documentation remains historical: among the 62 ethnobotanical sources included here, only approximately one-third were published after 2000. This temporal imbalance reflects persistent challenges, including restricted access to remote field sites, limited long-term funding for ethnobotanical research, logistical constraints in voucher collection, and ongoing underestimation of species diversity and use complexity. Together, these factors underscore the urgency of systematic documentation and interdisciplinary research that bridges traditional knowledge and contemporary neuropharmacology.

### 4.1 Ethnobotanical evidence and cultural context

This review documented 42 plant species traditionally used in Colombia for indications that may overlap with what biomedicine classifies as neuropsychiatric or neurological conditions. Solanaceae emerged as the most frequently reported family, followed by Lamiaceae and Apocynaceae, suggesting that these families may represent particularly important reservoirs of CNS-active taxa. At the genus level, *Brugmansia*, *Psychotria*, *Unonopsis*, and *Valeriana* were recurrently documented, indicating that certain genera may warrant broader comparative investigation across species.

Leaves, stems, and roots were the most frequently used plant parts, most commonly prepared as decoctions or infusions administered orally. While plant use may vary over time and between communities, species repeatedly documented across regions and cultural contexts—such as *N. tabacum* and *P. guajava*—likely reflect long processes of intergenerational knowledge transmission.

Importantly, the biomedical effect categories applied in this review (e.g., anxiolytic, sedative, hallucinogenic) are analytical constructs intended to facilitate comparison with pharmacological literature. They do not fully capture Indigenous epistemologies of health, illness, and healing. In many Colombian Indigenous knowledge systems, mental, emotional, somatic, and spiritual dimensions of wellbeing are inseparable, and illness is often understood as arising from social, cosmological, or spiritual imbalance rather than discrete neurobiological dysfunction (Schultes and Raffauf, 1992; Sarasola, 2010). Concepts such as *nervios* encompass broad symptom clusters that cannot be reduced to single DSM-5 diagnoses, and therapeutic practices often address relational and spiritual dimensions alongside bodily symptoms.

Hallucinogenic plants illustrate this epistemological divergence particularly clearly. Twelve species were identified as having hallucinogenic properties, many of which play central roles in ritual, diagnostic, and healing practices among Amazonian and Andean Indigenous Peoples. Preparations such as ayahuasca (yagé), primarily involving *B. caapi* in combination with *P. viridis* or *D. cabrerana*, are not merely psychoactive substances but integral components of cosmological healing systems. In these contexts, altered states of consciousness facilitate diagnosis, spiritual communication, social cohesion, and restoration of balance, functions that cannot be disentangled from ritual structure, healer expertise, and communal participation (Schultes and Raffauf, 1992). Translating traditional rituals into clinical findings must be approached cautiously when separating the therapeutic context from ritual structure, healer expertise, communal support, and cosmological meaning. Efficacy may depend critically on “set and setting” meaning the ritual structure including ceremonial songs and preparation, the presence of an experienced guide, community participation, and cosmological beliefs that contextualize the experience (Hartogsohn, 2017), which is inseparable from its therapeutic effect. Experienced guides or “medicine-men” serve essential safety functions, e.g., personalizing treatment doses, beyond psychological support.

Similarly, stimulant plants such as *E. coca* and *N. tabacum* (mambe, ambil) are widely used in social, ritual, and cognitive contexts, including collective deliberation, ceremonial exchange, and endurance during physical labor (Schultes, 1988). Their roles extend well beyond pharmacological stimulation and are deeply embedded in cultural identity and social organization.

Plants categorized here as used for “age-related cognitive decline” or “dementia” further illustrate the limits of biomedical translation. Indigenous descriptions of memory loss, confusion, or behavioral change may overlap phenomenologically with biomedical constructs of neurodegenerative disease, but their etiologies and therapeutic goals are often framed in spiritual or relational terms (Schultes, 1993). While pharmacological activity cannot be excluded, caution is required when interpreting these uses through a strictly biomedical lens.

### 4.2 Research gaps and priority species

Based on the convergence of ethnobotanical prominence and limited pharmacological investigation, six species were identified as priorities for future research: *I. fuchsioides*, *B. grandiflora*, *S. corallina*, *T. heterophylla*, *P. guajava*, and *D. pectoralis*. Evidence quality across these taxa varies substantially, ranging from absence of neuropharmacological studies to multiple preclinical investigations.

Several overarching research gaps emerge. First, phytochemical studies often focus on known or expected compounds, potentially overlooking minor constituents or synergistic interactions. Untargeted metabolomics approaches could help identify novel neuroactive fractions. Second, most pharmacological studies rely on isolated compounds rather than whole-plant extracts or preparation methods that reflect traditional use. Third, methodological heterogeneity—regarding plant identification, extraction protocols, dosing, and outcome measures—limits comparability and reproducibility across studies (Heinrich, 2010). The chemical complexity of natural products, the lack of standardization, and the high costs associated with patentability should not impede the translation of promising results into viable treatments (Corson and Crews, 2007), since alternatives are urgently needed. Further, commercial endeavours to create patents based on Indigenous knowledge and generating profit from this knowledge without consent are a major setback.

For the prioritized species, specific needs include mechanistic validation (*S. corallina*, *T. heterophylla*), chronic *in vivo* models (*I. fuchsioides*, *B. grandiflora*), neurotransmitter and receptor-level studies (*P. guajava*, *D. pectoralis*), and comprehensive safety profiling. With the exception of a small number of trials on *A. citrodora* and ayahuasca-related preparations, clinical evidence remains sparse, underscoring the need for phased translational research beginning with rigorous preclinical validation and Phase I safety studies.

#### 4.2.1 Cross-cutting mechanistic patterns and limitations

Across species with experimentally verified mechanisms, three dominant mechanistic patterns emerge.

- hallucinogenic species primarily act through serotonergic pathways, particularly 5-HT_2_A receptor agonism;
- sedative and anxiolytic species predominantly enhance GABAergic neurotransmission; and
- stimulant species target nicotinic, dopaminergic, or catecholaminergic systems.

Anticholinergic hallucinogens (*Brugmansia*, *Datura*) represent a distinct mechanistic class associated with significant toxicity risks.

### 4.3 Toxicology and safety considerations

Translating traditional plant use into modern therapeutic contexts requires careful attention to safety. Several species reviewed here—particularly *Brugmansia* spp. and *D. stramonium*—contain tropane alkaloids with narrow therapeutic windows and high toxicity at supratherapeutic doses (Sharma et al., 2021). Traditional Andean practitioners restrict their use to trained specialists following strict protocols developed over centuries to mitigate risk. These practices highlight the importance of contextual knowledge that cannot be assumed or replicated without careful study.

Ayahuasca preparations pose additional safety considerations due to β-carboline-mediated monoamine oxidase-A inhibition, which can lead to dangerous drug–drug and drug–food interactions (Brito-da-Costa, et al., 2020). Clinical trials have addressed these risks through strict exclusion criteria and washout periods, but such safeguards are essential for any future research or therapeutic application.

Even widely used species such as *N. tabacum* carry dose-dependent risks (Benowitz, 2010), reinforcing the need for standardized preparations, toxicity studies, and pharmacokinetic analyses before broader clinical consideration.

### 4.4 Sustainability and conservation

Although none of the species reviewed here are currently classified as threatened globally, increased research interest or commercialization could put pressure on wild populations. Sustainable harvesting, cultivation, and monitoring are therefore essential. Prioritizing widely distributed or cultivable species, developing good agricultural and collection practices, and supporting community-based production systems can help mitigate conservation risks.

Loss of natural habitats not only threatens biodiversity but also erodes Indigenous knowledge systems and local health practices, representing an irreversible loss of cultural and biomedical potential (Shanley and Luz, 2003). Conservation efforts must therefore be integrated with research and development strategies.

### 4.5 Ethical frameworks and indigenous rights

Traditional medicinal knowledge documented in this review is the intellectual heritage of Indigenous Peoples. Ethical research and any downstream application must recognize Indigenous sovereignty over this knowledge and comply with Colombian legal frameworks and international agreements, including the Nagoya Protocol (Heinrich et al., 2020, Nagoya Protocol, 2010). Free, prior, and informed consent, equitable benefit-sharing, and meaningful participation of Indigenous communities are essential prerequisites for future research or commercialization.

Many reviewed studies provide limited information on consent processes, co-authorship, or benefit-sharing mechanisms, reflecting broader structural challenges in ethnobotanical research. Future work should prioritize collaborative research models, transparent authorship practices, and reciprocal knowledge exchange.

### 4.6 Regional and global context

Comparative analyses suggest that Colombia shares patterns with other biodiverse regions, including high ethnobotanical richness coupled with limited pharmacological validation. Brazilian and African ethnopharmacological reviews report similar evidence gaps, while Indian Ayurvedic medicine benefits from more extensive clinical investigation of priority species (Cavalcante e Costa et al., 2018; Adhikari et al., 2020). The relatively high proportion of hallucinogenic plants documented in Colombia reflects distinct cultural approaches to diagnosis and healing rather than simple pharmacological preference.

Overall, the substantial validation gap identified here—28 of 42 species lacking neuropharmacological evidence—mirrors global trends of underinvestment in ethnopharmacological research. Addressing this gap requires sustained interdisciplinary collaboration, equitable partnerships, and long-term commitment to both scientific rigor and cultural respect.

## 5 Conclusion

This review highlights the substantial but largely underexplored potential of Colombian medicinal plants as sources of novel therapeutic approaches for neuropsychiatric and neurological disorders. From ethnobotanical documentation, 42 plant species were identified as traditionally used for central nervous system (CNS)–related indications. However, significant evidence gaps persist: while phytochemical information is available for 37 species, only 14 have been evaluated in neuropharmacological studies, and a limited subset has been examined in clinical trials. This disparity underscores the urgent need for more systematic, standardized, and methodologically rigorous research to bridge traditional knowledge and biomedical validation.

Six species emerged as particularly promising priorities for future investigation: *I. fuchsioides*, *B. grandiflora*, *S. corallina*, *T. heterophylla*, *P. guajava*, and *D. pectoralis*. They represent high-value candidates for targeted research programs aimed at elucidating mechanisms of action, safety profiles, and therapeutic potential for conditions such as anxiety disorders, depression, epilepsy, and age-related cognitive decline.

Advancing this field will require integrative and interdisciplinary approaches that combine ethnobotany, untargeted metabolomics, neuropharmacology, and emerging tools such as AI-assisted compound discovery. Ethnobotanical insights should guide hypothesis-driven phytochemical and mechanistic studies, followed by robust preclinical validation and well-designed clinical trials using botanically authenticated and chemically standardized preparations. Importantly, isolation of active compounds represents only an initial step; understanding synergistic effects, traditional preparation methods, pharmacokinetics, and safety is essential for responsible translation into therapeutic applications.

Ethical and sustainability considerations must remain central to future research and development. Indigenous Peoples are the custodians of the traditional knowledge documented in this review, and their rights to free, prior, and informed consent, equitable benefit-sharing, and cultural recognition must be upheld. At the same time, conservation and cultivation strategies are necessary to safeguard biodiversity and ensure that increased scientific or commercial interest does not compromise wild plant populations or ecosystem integrity. Ensuring equitable access to any resulting therapies, particularly for communities that have historically relied on these plants, is equally critical.

In a global context where neuropsychiatric disorders continue to rise and existing pharmacotherapies often show limited efficacy or accessibility, Colombia’s botanical diversity and ethnopharmacological heritage represent an invaluable, yet insufficiently realized, resource. Preserving and advancing traditional medicinal knowledge is therefore not only an act of cultural stewardship but also a strategic pathway toward discovering safer, more effective, and more accessible treatments. By responsibly integrating ancestral wisdom with rigorous scientific research, it is possible to unlock new therapeutic avenues while honoring cultural traditions and protecting ecological systems—ensuring that the healing potential of Colombia’s medicinal flora benefits both present and future generations.

## References

[B1] Abuhamdah S. Abuhamdah R. Howes M. J. R. Al-Olimat S. Ennaceur A. Chazot P. L. (2015). Pharmacological and neuroprotective profile of an essential oil derived from leaves of *Aloysia citrodora* Palau. J. Pharm. Pharmacol. 67 (9), 1306–1315. 10.1111/jphp.12424 25877296

[B2] Adewusi E. A. Steenkamp V. (2011). *In vitro* screening for acetylcholinesterase inhibition and antioxidant activity of medicinal plants from southern Africa. Asian pac. J. Trop. Med. 4 (10), 829–835. 10.1016/S1995-7645(11)60203-4 22014742

[B125] Adhikari T. Srikanth N. Juneja A. Sharma S. Maulik (2020). Overview of Ayurveda trials registered with Clinical Trial Registry‐India: need for customized data set items. AYU 41, 143–147. 10.4103/ayu.ayu_375_21 35370375 PMC8966761

[B3] Afrasiabian F. Mirabzadeh Ardakani M. Rahmani K. Azadi N. A. Alemohammad Z. B. Bidaki R. (2019). *Aloysia citrodora* Palau (lemon verbena) for insomnia patients: a randomized, double-blind, placebo-controlled clinical trial. Phytother. Res. 33 (2), 350–359. 10.1002/ptr.6228 30450627

[B4] Alarcón-Ruiz C. A. Maguiña J. L. Apolaya-Segura M. Carhuapoma-Yance M. Aranda-Ventura J. Herrera-Añazco P. (2023). Bibliometric analysis of medicinal plants’ original articles from Latin America and the Caribbean region. J. Scientometr. Res. 12 (1), 79–91. 10.5530/jscires.12.1.011

[B5] Alvarado-García P. A. A. Soto-Vásquez M. R. Rosales-Cerquin L. E. Alfaro-Ttito B. M. Rodrigo-Villanueva E. M. (2021). Anxiolytic-like effect of essential oils extracted from *Lippia alba* and *Lippia citriodora* . Pharmacogn. J. 13, 1377–1383. 10.5530/pj.2021.13.175

[B6] American Psychiatric Association (2013). Diagnostic and statistical manual of mental disorders (DSM-5). Washington, DC: APA. 10.1176/appi.books.9780890425596

[B7] Andrade M. T. Lima J. A. Pinto A. C. Rezende C. M. Carvalho M. P. Epifanio R. A. (2005). Indole alkaloids from *Tabernaemontana australis* that inhibit acetylcholinesterase. Bioorg. Med. Chem. 13 (12), 4092–4095. 10.1016/j.bmc.2005.03.045 15911323

[B8] Aragão G. F. Carneiro L. M. Júnior A. P. Vieira L. C. Bandeira P. N. Lemos T. L. (2006). A possible mechanism for anxiolytic and antidepressant effects of α- and β-amyrin from *Protium heptaphyllum* . Pharmacol. Biochem. Behav. 85 (4), 827–834. 10.1016/j.pbb.2006.11.019 17207523

[B9] Arévalo D. Martínez C. A. Rincón J. Guerrero M. F. (2006). Fracción alcaloidal obtenida de *Valeriana pavonii* Poepp con actividad anticonvulsivante. Rev. Colomb. Cienc. Quím. Farm. 35, 168–176.

[B10] Arias H. R. De Deurwaerdere P. Scholze P. Sakamoto S. Hamachi I. Di Giovanni G. (2023). Coronaridine congeners induce sedative and anxiolytic-like activity by allosteric modulation of GABAA receptors. Eur. J. Pharmacol. 953, 175854. 10.1016/j.ejphar.2023.175854 37331683

[B11] Asmundson G. J. G. Taylor S. (2020). Coronaphobia: fear and the COVID-19 outbreak. J. Anxiety Disord. 70, 102196. 10.1016/j.janxdis.2020.102196 32078967 PMC7134790

[B12] Awad R. Ahmed F. Bourbonnais-Spear N. Mullally M. Ta C. A. Tang A. (2009). Ethnopharmacology of Q’eqchi’ maya antiepileptic and anxiolytic plants. J. Ethnopharmacol. 125 (2), 257–264. 10.1016/j.jep.2009.06.034 19591913

[B13] Ayres A. S. Santos W. B. Junqueira-Ayres D. D. Costa G. M. Ramos F. A. Castellanos L. (2017). Monoaminergic neurotransmission mediates antidepressant-like effects of *Passiflora edulis* . Neurosci. Lett. 660, 79–85. 10.1016/j.neulet.2017.09.010 28893593

[B14] Barbosa P. R. Valvassori S. S. Bordignon C. L. Kappel V. D. Martins M. R. Gavioli E. C. (2008). Aqueous extracts of *passiflora* species reduce anxiety-related behaviors. J. Med. Food 11 (2), 282–288. 10.1089/jmf.2007.722 18598170

[B15] Barbosa P. C. R. Cazorla I. M. Giglio J. S. Strassman R. (2009). Six-month evaluation of psychiatric symptoms in ayahuasca-naïve subjects. J. Psychoact. Drugs 41 (3), 205–212. 10.1080/02791072.2009.10400530 19999673

[B16] Barczyk Z. A. Rucklidge J. J. Eggleston M. Mulder R. T. (2020). Psychotropic medication prescription trends in youth. J. Child. Adolesc. Psychopharmacol. 30 (2), 87–96. 10.1089/cap.2019.0032 31633377

[B17] Bender S. Grohmann R. Engel R. Degner D. Dittmann-Balcar A. Rüther E. (2004). Severe adverse drug reactions in psychiatric inpatients. Pharmacopsychiatry 37 (S1), 46–53. 10.1055/s-2004-815510 15052514

[B18] Benowitz N. L. (2010). Nicotine addiction. New Engl. J. Med. 362 (24), 2295–2303. 10.1056/NEJMra0809890 20554984 PMC2928221

[B19] Biswas S. Mondol D. Jodder P. Sana S. Saleh M. Tarafdar A. K. (2021). Neurobehavioral activities of *Psidium guajava* leaves in mice. Future J. Pharm. Sci. 7 (1), 1–12. 10.1186/s43094-021-00188-5

[B20] Bouso J. C. Palhano-Fontes F. Rodríguez-Fornells A. Ribeiro S. Sanches R. Crippa J. A. S. (2015). Long-term psychedelic use and brain structure. Eur. Neuropsychopharmacol. 25 (4), 483–492. 10.1016/j.euroneuro.2015.01.008 25637267

[B21] Bracci A. Daza-Losada M. Aguilar M. De Feo V. Miñarro J. Rodríguez-Arias M. (2013). *Brugmansia arborea* extract modulates morphine and cocaine effects. Evid. Based Complement. Altern. Med. 2013, 482976. 10.1155/2013/482976 23533488 PMC3606722

[B120] Breijyeh Z. Karaman R. (2020). Comprehensive review on Alzheimer’s disease: causes and treatment. Molecules 25, 5789. 10.3390/molecules25245789 33302541 PMC7764106

[B22] Brito-da-Costa A. M. Dias-da-Silva D. Gomes N. G. Dinis-Oliveira R. J. Madureira-Carvalho Á. (2020). Toxicokinetics and toxicodynamics of ayahuasca alkaloids N, N-dimethyltryptamine (DMT), harmine, harmaline and tetrahydroharmine: clinical and forensic impact. Pharmaceuticals 13 (11), 334. 10.3390/ph13110334 33114119 PMC7690791

[B23] Brunton L. L. Knollmann B. C. (2023). Goodman and gilman's the pharmacological basis of therapeutics. 14th ed. (McGraw-Hill Education).

[B24] Bum E. N. Ngah E. Ekoundi B. C. Dong C. Mbomo R. E. A. Rakotonirina S. (2004). Sedative and anticonvulsant properties of *Passiflora edulis* . Afr. J. Tradit. Complement. Altern. Med. 1 (1), 63–71.

[B25] Bystriakova N. Tovar C. Monro A. Moat J. Hendrigo P. Carretero J. (2021). Colombia's bioregions as a source of useful plants. PLoS One 16 (8), e0256457. 10.1371/journal.pone.0256457 34449804 PMC8396733

[B26] Carmona F. Angelucci M. A. Sales D. S. Chiaratti T. M. Honorato F. B. Bianchi R. V. (2013). *Lippia alba* extract in migraine treatment. Phytomedicine 20 (10), 947–950. 10.1016/j.phymed.2013.03.017 23639189

[B27] Caro S. A. (2004). Ethnobotanical studies in the central andes (colombia): knowledge distribution of plant use according to informant’s characteristics. Lyonia 7 (2), 89–104.

[B28] Cavalcante e Costa G. F. Nishijo H. Caixeta L. F. Aversi-Ferreira T. A. (2018). The confrontation between ethnopharmacology and pharmacological tests of medicinal plants associated with mental and neurological disorders. Evid. Based Complement. Altern. Med. 2018, 7686913. 10.1155/2018/7686913 30057646 PMC6051267

[B29] Celis C. T. Rincón J. Guerrero M. F. (2007). CNS activity of *Valeriana pavonii* extracts. Rev. Colomb. Cienc. Quím. Farm. 36 (1), 11–22.

[B30] Cipriani A. Furukawa T. A. Salanti G. Chaimani A. Atkinson L. Z. Ogawa Y. (2018). Comparative efficacy of antidepressants. Lancet 391, 1357–1366. 10.1016/S0140-6736(17)32802-7 29477251 PMC5889788

[B31] Clardy J. Walsh C. (2004). Lessons from natural molecules. Nature 432, 829–837. 10.1038/nature03194 15602548

[B32] Coleta M. Batista M. T. Campos M. G. Carvalho R. Cotrim M. D. Lima T. C. M. (2006). Neuropharmacological evaluation of the putative anxiolytic effects of *Passiflora edulis* sims, its subfractions and flavonoid constituents. Phytother. Res. 20 (12), 1067–1073. 10.1002/ptr.1999 17009209

[B33] Conde R. Corrêa V. S. Carmona F. Contini S. H. Pereira A. M. (2011). Chemical composition and therapeutic effects of *Lippia alba* leaves hydroalcoholic extract in migraine patients. Phytomedicine 18 (14), 1197–1201. 10.1016/j.phymed.2011.06.016 21802924

[B34] Corson T. W. Crews C. M. (2007). Molecular understanding and modern application of traditional medicines: triumphs and trials. Cell 130 (5), 769–774. 10.1016/j.cell.2007.08.031 17803898 PMC2507744

[B35] Costa J. Ferreira P. De Sousa D. Jordan J. Freitas R. (2012). Anticonvulsant effect of phytol in a pilocarpine model in mice. Neurosci. Lett. 523 (2), 115–118. 10.1016/j.neulet.2012.05.011 22750154

[B36] De Barros G. S. Silva C. M. M. de Abreu Matos F. J. (2000). Anticonvulsant activity of essential oils and active principles from chemotypes of *Lippia alba* . Biol. Pharm. Bull. 23 (11), 1314–1317. 10.1248/bpb.23.1314 11085358

[B37] De Smet P. A. (1983). A multidisciplinary overview of intoxicating enema rituals in the Western hemisphere. J. Ethnopharmacol. 9 (2–3), 129–166. 10.1016/0378-8741(83)90031-4 6677814

[B38] Diazgranados M. (2022). “A taxonomic summary of useful plants in Colombia,” in Catalogue of useful plants of Colombia. Editors Negrão R. Monro A. Castellanos-Castro C. Diazgranados M. (Kew and Bogotá: Royal Botanic Gardens, Kew and Instituto Humboldt), 135–147.

[B39] Diazgranados M. Cossu T. Kor L. Gori B. Torres-Morales G. Aguilar-Giraldo A. (2022). “Annotated checklist of useful plants of Colombia,” in Catalogue of useful plants of Colombia. Editors Negrão R. Monro A. Castellanos-Castro C. Diazgranados M. (Bogotá: Royal Botanic Gardens, Kew and the Humboldt Institute), 165–437.

[B41] Dos Santos R. G. Osório F. L. Crippa J. A. Riba J. Zuardi A. W. Hallak J. E. (2016). Antidepressive, anxiolytic, and antiaddictive effects of ayahuasca, psilocybin, and LSD. Ther. Adv. Psychopharmacol. 6 (3), 193–213. 10.1177/2045125316638008 27354908 PMC4910400

[B42] Ezeja M. Omeh Y. (2010). Antinociceptive activities of methanolic leaf extract of *Nicotiana tabacum* . Cont. J. Pharmacol. Toxicol. Res. 3, 5–10.

[B44] GBD Mental Disorders Collaborators (2022). Global burden of 12 mental disorders, 1990–2019. Lancet Psychiatry 9 (2), 137–150. 10.1016/S2215-0366(21)00395-3 35026139 PMC8776563

[B45] Giraldo S. E. Rincón J. Puebla P. Marder M. Wasowski C. Vergel N. (2010). Isovaleramide, an anticonvulsant molecule isolated from *Valeriana pavonii* . Biomédica 30 (2), 245–250. 10.7705/biomedica.v30i2.187 20890571

[B46] Giraldo Quintero S. E. Bernal Lizarazú M. C. Morales Robayo A. Pardo Lobo A. Z. Gamba Molano L. (2015). Traditional medicinal plant use in Bogotá markets. Nova 13 (23). 10.22490/24629448.1707

[B47] Gobbi S. Płomecka M. B. Ashraf Z. Radziński P. Neckels R. Lazzeri S. (2020). Worsening of preexisting psychiatric conditions during COVID-19. Front. Psychiatry 11, 581426. 10.3389/fpsyt.2020.581426 33391049 PMC7772353

[B48] Gonzalez-Trujano M. E. Paez-Martinez N. Krengel F. Martinez-Vargas D. Leon-Santiago M. Cruz-Lopez B. (2023). CNS activity of a *Tabernaemontana arborea* alkaloid extract. Fitoterapia 169, 105602. 10.1016/j.fitote.2023.105602 37423501

[B49] Guerra-Doce E. (2015). Psychoactive substances in prehistoric times. Time Mind 8 (1), 91–112. 10.1080/1751696X.2014.993244

[B50] Hálfdánarson Ó. Zoëga H. Aagaard L. Bernardo M. Brandt L. Fusté A. C. (2017). International trends in antipsychotic use. Eur. Neuropsychopharmacol. 27 (10), 1064–1076. 10.1016/j.euroneuro.2017.07.001 28755801

[B51] Hannan M. A. Dash R. Haque M. Choi S. M. Moon I. S. (2020). Neuropharmacological actions of *Withania somnifera* in Alzheimer’s disease. CNS Neurol. Disord. Drug Targets 19 (7), 541–556. 10.2174/1871527319999200730214807 32748763

[B52] Hartogsohn I. (2017). Constructing drug effects: a history of set and setting. Drug Sci. Policy Law 3, 2050324516683325. 10.1177/2050324516683325

[B53] Harzing A. W. (2010). The publish or perish book. Melbourne: Tarma Software Research.

[B54] Heinrich M. (2010). Ethnopharmacology in the 21st century-grand challenges. Front. Pharmacol. 1, 8. 10.3389/fphar.2010.00008 21713103 PMC3112271

[B55] Heinrich M. Scotti F. Andrade-Cetto A. Berger-Gonzalez M. Echeverría J. Friso F. (2020). Access and benefit sharing under the Nagoya protocol—Quo vadis? Six Latin American case studies assessing opportunities and risk. Front. Pharmacol. 11, 765. 10.3389/fphar.2020.00765 32581783 PMC7294742

[B122] Heldwein C. G. Silva L. L. Reckziegel P. Barros F. M. C. Bürger M. E. Baldisserotto B. (2012). Participation of the GABAergic system in the anesthetic effect of Lippia alba (Mill.) N.E. Brown essential oil. Braz. J. Med. Biol. Res. 45, 436–443. 10.1590/S0100-879X2012005000047 22473320 PMC3854290

[B56] Hooijmans C. R. Rovers M. M. de Vries R. B. Leenaars M. Ritskes-Hoitinga M. Langendam M. W. (2014). SYRCLE’s risk of bias tool for animal studies. BMC Med. Res. Methodol. 14, 43. 10.1186/1471-2288-14-43 24667063 PMC4230647

[B57] Huhn M. Nikolakopoulou A. Schneider-Thoma J. Krause M. Samara M. Peter N. (2019). Comparative efficacy and tolerability of antipsychotics in schizophrenia. Lancet 394 (10202), 939–951. 10.1016/S0140-6736(19)31135-3 31303314 PMC6891890

[B58] Itokawa H. Morris-Natschke S. L. Akiyama T. Lee K. H. (2008). Plant-derived natural product research aimed at new drug discovery. J. Nat. Med. 62 (3), 263–280. 10.1007/s11418-008-0246-z 18425692

[B59] Kitagawa T. Ikeda Y. Suzuki T. Mimura M. Leucht S. Huhn M. (2024). Efficacy and effectiveness of antipsychotics in schizophrenia: network meta-analyses combining randomised trials and real-world data. Lancet Psychiatry 11 (7), 554–565. 10.1016/S2215-0366(24)00136-8 38215784

[B60] Kupeli Akkol E. Tatli Cankaya I. Seker Karatoprak G. Carpar E. Sobarzo-Sanchez E. Capasso R. (2021). Natural compounds as medical strategies in psychiatric disorders associated with neurological diseases. Front. Pharmacol. 12, 669638. 10.3389/fphar.2021.669638 34054540 PMC8155682

[B61] Kuypers K. P. C. Riba J. De La Fuente Revenga M. Barker S. Theunissen E. L. Ramaekers J. G. (2016). Ayahuasca enhances creative divergent thinking while decreasing convergent thinking. Psychopharmacology 233 (18), 3395–3403. 10.1007/s00213-016-4377-8 27435062 PMC4989012

[B62] Li H. Zhou P. Yang Q. Shen Y. Deng J. Li L. (2011). Comparative anxiolytic activities and flavonoid composition of *Passiflora edulis* ‘edulis’ and ‘flavicarpa. J. Ethnopharmacol. 133 (3), 1085–1090. 10.1016/j.jep.2010.11.039 21111038

[B63] Llamazares A. M. Sarasola C. M. Aires B. (2004). “Main sacred plants in South America,” in El lenguaje de los dioses: arte, chamanismo y cosmovisión indígena en Sudamérica (Buenos Aires), 259–285.

[B64] Lorena C. Ressaissi A. Serralheiro M. L. (2022). Bioactives from *Psidium guajava* leaf decoction: identification, bioactivities and bioavailability. Food Chem. Adv. 1, 100003. 10.1016/j.focha.2022.100003

[B121] Mattioli L. Bracci A. Titomanlio F. Perfumi M. De Feo V. (2012). Effects of Brugmansia arborea extract and its secondary metabolites on morphine tolerance and dependence in mice. Evid. Based Complement. Alternat. Med. 2012, 741925. 10.1155/2012/741925 22454681 PMC3290905

[B65] Martínez-Rodríguez A. Martínez-Olcina M. Mora J. Navarro P. Caturla N. Jones J. (2022). Anxiolytic effect and improved sleep quality with *Lippia citriodora* extract. Nutrients 14 (1), 218. 10.3390/nu14010218 35011093 PMC8747367

[B66] McKenna D. J. Ruiz J. M. Hoye T. R. Roth B. L. Shoemaker A. T. (2011). Receptor screening technologies in Amazonian ethnomedicines. J. Ethnopharmacol. 134 (2), 475–492. 10.1016/j.jep.2010.12.037 21232588

[B67] Miller M. J. Albarracin-Jordan J. Moore C. Capriles J. M. (2019). Chemical evidence for use of multiple psychotropic plants in a 1,000-year-old ritual bundle. Proc. Natl. Acad. Sci. U.S.A. 116 (23), 11207–11212. 10.1073/pnas.1902174116 31061128 PMC6561276

[B68] Ministerio de Protección Social (2008). Vademécum Colombiano de Plantas Medicinales. Bogotá: Imprenta Nacional de Colombia.

[B69] Moncayo P. Werley Y. Sanabria Diago O. L. (2022). Plantas y prácticas de conservación de la medicina tradicional en el suroriente de El Tambo, Cauca, Colombia. Bot. Sci. 100 (4), 935–959. 10.17129/botsci.3056

[B70] Moosavyzadeh A. Mokri A. Ghaffari F. Faghihzadeh S. Azizi H. Jafari Hajati R. (2020). Hab-o Shefa for maintenance treatment of opioid dependence: a randomized placebo-controlled trial. J. Altern. Complement. Med. 26 (5), 376–383. 10.1089/acm.2019.0390 32109133

[B71] Morales-García J. A. de la Fuente Revenga M. Alonso-Gil S. Rodríguez-Franco M. I. Feilding A. Perez-Castillo A. (2017). Alkaloids of *Banisteriopsis caapi* stimulate adult neurogenesis *in vitro* . Sci. Rep. 7, 5309. 10.1038/s41598-017-05407-9 28706205 PMC5509699

[B72] Nagoya Protocol (2010). Nagoya protocol on access to genetic resources and the fair and equitable sharing of benefits arising from their utilization to the convention on biological diversity. Secr. Convention Biol. Divers. Available online at: https://www.cbd.int/abs/doc/protocol/nagoya-protocol-en.pdf (Accessed December 15, 2025).

[B73] Nencini C. Cavallo F. Bruni G. Capasso A. De Feo V. De Martino L. (2006). Affinity of *Iresine herbstii* and *Brugmansia arborea* extracts for cerebral receptors. J. Ethnopharmacol. 105 (3), 352–357. 10.1016/j.jep.2005.11.022 16406412

[B123] NHS Business Services Authority (2023). Medicines used in mental health - Central nervous system (CNS) stimulants and drugs used for ADHD. Available online at: https://nhsbsa-opendata.s3.eu-west-2.amazonaws.com/mumh/mumh_quarterly_dec22_v001.html (Accessed March 7, 2026).

[B74] O’Shaughnessy D. M. Berlowitz I. Rodd R. Sarnyai Z. Quirk F. (2021). Changes in addiction treatment using traditional Amazonian medicine. Ther. Adv. Psychopharmacol. 11, 2045125320986634. 10.1177/2045125320986634 33717431 PMC7841703

[B75] Palhano-Fontes F. Barreto D. Onias H. Andrade K. C. Novaes M. M. Pessoa J. A. (2019). Rapid antidepressant effects of ayahuasca in treatment-resistant depression. Psychol. Med. 49 (4), 655–663. 10.1017/S0033291718001356 29903051 PMC6378413

[B76] Parodi T. V. Cunha M. A. Becker A. G. Zeppenfeld C. C. Martins D. I. Koakoski G. (2014). Anesthetic activity of *Aloysia triphylla* essential oil in fish. Fish. Physiol. Biochem. 40 (2), 323–334. 10.1007/s10695-013-9845-z 23974669

[B77] Patgiri B. J. Umretiya C. H. Vaishnani P. P. (2022). Overview of ayurveda trials registered with clinical trial registry-india. J. Ayurveda Integr. Med. 13 (1), 100–110. 10.1016/j.jaim.2022.01.002

[B78] Peredery O. Persinger M. A. (2004). Herbal treatment following post-seizure induction in rats. Phytother. Res. 18 (9), 700–705. 10.1002/ptr.1511 15478209

[B117] Posadzki P. Watson L. Ernst E. (2013). Herb–drug interactions: an overview of systematic reviews. Br. J. Clin. Pharmacol. 75, 603–618. 10.1111/j.1365-2125.2012.04350.x 22670731 PMC3575928

[B79] Puniani E. Cayer C. Kent P. Mullally M. Sánchez-Vindas P. Álvarez L. P. (2015). Ethnopharmacology of *souroubea* spp. and betulinic acid as an anxiolytic principle. Phytochemistry 113, 73–78. 10.1016/j.phytochem.2014.02.017 24641939

[B80] Raffauf R. F. Shemluck M. J. Le Quesne P. W. (1991). Withanolides of *Iochroma fuchsioides* . J. Nat. Prod. 54 (6), 1601–1606. 10.1021/np50078a017

[B81] Rashidian A. Farhang F. Vahedi H. Dehpour A. R. Mehr S. E. Mehrzadi S. (2016). Anticonvulsant effects of *Lippia citriodora* leaves ethanolic extract. Int. J. Prev. Med. 7, 97. 10.4103/2008-7802.187251 27563433 PMC4977972

[B82] Razavi B. M. Zargarani N. Hosseinzadeh H. (2017). Anti-anxiety and hypnotic effects of *Lippia citriodora* extracts and verbascoside. Avicenna J. Phytomed. 7 (4), 353–365. 28884085 PMC5580873

[B83] Rodríguez-Chanfrau J. E. López Hernández O. D. Núñez Figueredo Y. Rodríguez Ferrada C. A. Nogueira Mendoza A. (2013). Obtention of dry extract from aqueous extracts of *Justicia pectoralis* . Rev. Cuba. Plantas Med. 18 (4), 543–554.

[B119] Rodríguez J. L. Mateos R. Palomino O. Fernández-Alfonso M. S. Ramos-Cevallos N. Inostroza-Ruiz L. (2023). Cytoprotective‐antioxidant effect of *Brunfelsia grandiflora* extract on neuron-like cells. Appl. Sci. 13 (22), 12233. 10.3390/app132212233

[B84] Sabti M. Sasaki K. Gadhi C. Isoda H. (2019). Molecular mechanisms of *lippia citriodora*-induced relaxation and antidepressant effects. Int. J. Mol. Sci. 20 (14), 3556. 10.3390/ijms20143556 31330819 PMC6678442

[B85] Samoylenko V. Rahman M. M. Tekwani B. L. Tripathi L. M. Wang Y. H. Khan S. I. (2010). *Banisteriopsis caapi*: MAO inhibition and antioxidative constituents. J. Ethnopharmacol. 127 (2), 357–367. 10.1016/j.jep.2009.10.030 19879939 PMC2828149

[B116] Sandson N. B. Armstrong S. C. Cozza K. L. (2005). An overview of psychotropic drug‐drug interactions. Psychosomatics 46, 464–494. 16145193 10.1176/appi.psy.46.5.464

[B118] Sarasola C. M. (2010). De manera sagrada y en celebración: identidad, cosmovisión y espiritualidad en los pueblos indígenas. Buenos Aires: Editorial Biblos.

[B86] Sanches R. F. de Lima Osório F. Dos Santos R. G. Macedo L. R. Maia-de-Oliveira J. P. Wichert-Ana L. (2016). Antidepressant effects of ayahuasca: a SPECT study. J. Clin. Psychopharmacol. 36 (1), 77–81. 10.1097/JCP.0000000000000436 26650973

[B87] Sarris J. Perkins D. Cribb L. Schubert V. Opaleye E. Bouso J. C. (2021). Ayahuasca use and reported effects on depression and anxiety. J. Affect. Disord. Rep. 4, 100098. 10.1016/j.jadr.2021.100098

[B88] Schultes R. E. (1988). Where the gods reign: plants and peoples of the Colombian amazon. Oracle, AZ: Synergetic Press.

[B89] Schultes R. E. (1993). Plants in treating senile dementia in the northwest amazon. J. Ethnopharmacol. 38 (2–3), 121–128. 10.1016/0378-8741(93)90007-R 8510460

[B90] Schultes R. E. Raffauf R. F. (1992). Vine of the soul: medicine men, their plants and rituals in the Colombian Amazonia. Oracle: Synergetic Press.

[B91] Serrano-Dueñas M. Cardozo-Pelaez F. Sánchez-Ramos J. R. (2001). Effects of *Banisteriopsis caapi* extract on Parkinson’s disease. Sci. Rev. Altern. Med. 5 (3), 127–132.

[B92] Shaheen H. M. Ali B. H. Alqarawi A. A. Bashir A. K. (2000). Effect of *Psidium guajava* leaves on some aspects of the central nervous system in mice. Phytother. Res. 14 (2), 107–111. 10.1002/(SICI)1099-1573(200003)14:2<107:AID-PTR580>3.0.CO;2-Z 10685107

[B93] Shanley P. Luz L. (2003). The impacts of forest degradation on medicinal plant use and implications for health care in eastern Amazonia. BioScience 53 (6), 573–584. 10.1641/0006-3568(2003)053[0573:TIOFDO]2.0.CO;2

[B94] Sharma Y. Kundu N. Srivastava N. S. Bala K. (2017). Neuroprotective ability of tobacco stem silver nanoparticles on rat PC-12 cells. Asian J. Pharm. 11 (4).

[B95] Sharma M. Dhaliwal I. Rana K. Delta A. K. Kaushik P. (2021). Phytochemistry, pharmacology, and toxicology of datura species—A review. Antioxidants 10 (8), 1291. 10.3390/antiox10081291 34439539 PMC8389218

[B96] Shen H. W. Jiang X. L. Winter J. C. Yu A. M. (2010). Psychedelic 5-methoxy-N,N-dimethyltryptamine: metabolism, pharmacokinetics, drug interactions, and pharmacological actions. Curr. Drug Metab. 11 (8), 659–666. 10.2174/138920010791678900 20942780 PMC3028383

[B97] Soares D. Duarte L. P. Cavalcanti A. D. Silva F. C. Braga A. D. Lopes M. T. (2017). *Psychotria viridis*: chemical constituents from leaves and biological properties. An. Acad. Bras. Cienc. 89, 927–938. 10.1590/0001-3765201720160411 28640347

[B98] Soler J. Elices M. Franquesa A. Barker S. Friedlander P. Feilding A. (2016). Exploring the therapeutic potential of ayahuasca: acute intake increases mindfulness-related capacities. Psychopharmacology 233 (5), 823–829. 10.1007/s00213-015-4162-0 26612618

[B99] Sterne J. A. C. Savović J. Page M. J. Elbers R. G. Blencowe N. S. Boutron I. (2019). RoB 2: a revised tool for assessing risk of bias in randomised trials. BMJ 366, l4898. 10.1136/bmj.l4898 31462531

[B100] Stroup T. S. Gray N. (2018). Management of common adverse effects of antipsychotic medications. World Psychiatry 17 (3), 341–356. 10.1002/wps.20567 30192094 PMC6127750

[B101] Tanner C. M. Goldman S. M. Aston D. A. Ottman R. Ellenberg J. Mayeux R. (2002). Smoking and Parkinson’s disease in twins. Neurology 58 (4), 581–588. 10.1212/WNL.58.4.581 11865136

[B102] Thomas G. Lucas P. Capler N. R. Tupper K. W. Martin G. (2013). Ayahuasca-assisted therapy for addiction: results from a preliminary observational study in Canada. Curr. Drug Abuse Rev. 6 (1), 30–42. 10.2174/15733998113099990003 23627784

[B103] Treede R. D. Rief W. Barke A. Aziz Q. Bennett M. I. Benoliel R. (2015). A classification of chronic pain for ICD-11. Pain 156 (6), 1003–1007. 10.1097/j.pain.0000000000000160 25844555 PMC4450869

[B104] Trujillo E. Frausin Bustamante G. Correa M. Trujillo W. (2010). El uso de la ayahuasca en la Amazonia. Ing. Amaz. 3 (2), 151–163.

[B105] Vargas M. V. Dunlap L. E. Dong C. Carter S. J. Tombari R. J. Jami S. A. (2023). Psychedelics promote neuroplasticity through activation of intracellular 5-HT2A receptors. Science 379 (6633), 700–706. 10.1126/science.adf0435 36795823 PMC10108900

[B106] Venâncio E. T. Rocha N. Rios E. Feitosa M. Linhares M. Melo F. (2011). Anxiolytic-like effects of standardized extract of *Justicia pectoralis* in mice: involvement of GABA/benzodiazepine receptors. Phytother. Res. 25 (3), 444–450. 10.1002/ptr.3274 20737656

[B107] Venâncio E. T. de Souza A. G. de Lima K. A. de Carvalho M. A. J. de Souza D. A. A. Oliveira J. V. S. (2019). Evaluation of monoaminergic levels after acute administration of *Justicia pectoralis* extract in mice. Rev. Cuba. Farm. 51 (4).

[B108] Wang C. Xu F. Q. Shang J. H. Xiao H. Fan W. W. Dong F. W. (2013). Cycloartane triterpenoid saponins from *Passiflora edulis* and their antidepressant-like effects. J. Ethnopharmacol. 148 (3), 812–817. 10.1016/j.jep.2013.05.010 23702036

[B109] Weller S. C. (1983). New data on intracultural variability: the hot–cold concept of medicine and illness. Hum. Organ. 42 (3), 249–257. 10.17730/humo.42.3.v485x5npq050g748

[B110] Wolter Filho W. Pinheiro M. L. B. Rocha A. I. D. (1983). Alcaloides de *Tabernaemontana heterophylla* Vahl (Apocynaceae). Acta Amaz. 13 (2), 409–412. 10.1590/1809-43921983132409

[B111] World Health Organization (2009). Pharmacological treatment of mental disorders in primary health care. Geneva: World Health Organization. 23762966

[B112] World Health Organization (2019). *International classification of diseases for mortality and morbidity statistics* (11th revision). Geneva: World Health Organization.

[B113] Xie D. Yao L. Huang Y. Wu S. Ma L. Li Y. (2021). Anxiolytic effect of two tobacco essential oils (*Nicotiana tabacum* L.) in mice. Molecules 26 (14), 4171. 10.3390/molecules26144171 34299447 PMC8306096

[B114] Zétola M. De Lima T. Sonaglio D. González-Ortega G. Limberger R. Petrovick P. (2002). CNS activities of liquid and spray-dried extracts from *Lippia alba* (verbenaceae). J. Ethnopharmacol. 82 (2–3), 207–215. 10.1016/S0378-8741(02)00187-3 12241997

[B115] Zhao J. Nakamura N. Hattori M. Kuboyama T. Tohda C. Komatsu K. (2002). Withanolide derivatives from the roots of *Withania somnifera* and their neurite outgrowth activities. Chem. Pharm. Bull. 50 (6), 760–765. 10.1248/cpb.50.760 12045329

