# Supplementary material for: Exploring Colombian medicinal flora used in indigenous and campesino health systems for neuropsychiatric disorders and neuropharmacological potential: an ethnopharmacological review
Source: Front Pharmacol. 2026 Mar 11;17:1729887. doi: 10.3389/fphar.2026.1729887 (PMC13013478; doi:10.3389/fphar.2026.1729887)
Supplement: Supplementary file 1 [file Table1.docx]

**Supplementary Table 1. An overview of traditional uses of selected medicinal plant species together with their plant parts used, ways of administration, vernacular names, principal medicinal indications, and related publications.** Vernacular names obtained from the Annotated checklist of useful plants of Colombia (Diazgranados, et al. 2022a). (N/A: not applicable; no vernacular name record in ethnobotanical sources)

| **Plant species** | **Vernacular name(s)** | **Family** | **Region/ Community** | **Plant parts used** | **Mode of preparation** | **Way of administration** | **Principal traditional indication** | **References** |
| --- | --- | --- | --- | --- | --- | --- | --- | --- |
| *Adenocalymma schomburgkii* (DC.) L.G.Lohmann | bejuco mataganado | Bignoniaceae | Northwest (NW) Amazon / Ticuna, Siona, Yukuna, Tanimuka, Kubeos, Kofán | leaves | powder | oral ingestion | confusion delusion in elderly | (Schultes, 1993) |
| *Aloysia citrodora* Paláu | alegría, cedrón, cidrón, luisa, luisa de Chile, yerbaluisa, zorrillo, saca ojo, lemon verbe yerba luisa | Verbenaceae | Bogotá (urban markets) / Urban/rural consumers  Antioquia, Andes / Rural households and communities (San Cristóbal)  Boyacá, Andes / Rural communities | complete plant used, leaves, stems | infusion, decoction | oral ingestion | stress  nervousness  sleep disorder  insomnia  depression  general indisposition  anxiety  pain (other/neuralgic) spasms  stomach and intestinal complaints  stomachache  indigestion  diarrhea  flatulence  nausea/vomitingbronchitis  congestion  flu  heart conditions  hypotension  circulation | (Angulo et al., 2012; Giraldo Quintero et al., 2015; Lagos-López, 2007; Marín and Gómez, 2015; Puyo Anacona, 2018; Ministerio de la Protección Social, 2008; Vera-Marín and Sánchez-Sáen, 2016) |
| *Anadenanthera peregrina* (L.) Speg. | carripaco, cohoba, cojoba, dopa, dopamagerná, dopanae (Sikuani), dopane (Cuiba), guahibo, lomo de caimán, niopo, yompa, yopa, yopo, yoto, yumpa, yupa | Fabaceae | Colombian Llanos / Sikuani, Cuiba  NW Amazon & Vaupés region – Tukanoan groups  Middle Orinoco / Eastern Colombia–Venezuela region – Piaroa | seeds | powder, snuff | enema, inhalation | illness diagnosis spiritual purification ritual healing  social/ceremonial use | (De Smet, 1983, 1985; Schultes, 1942, 1979b) |
| *Banisteriopsis caapi* (Spruce ex Griseb.) Morton | ayahuasca, bejuco bravo, bejuco de oro, bejuco dulce, caapi, caji, caji-idirecaii, capi, capi o caapi, changropanga, cordón umbilical, dapa, kapi, liana del napo, natema, oofa, pejí, pildé, pinde, yaco borrachero, yagé, yagé de cacería, yagé de monte, yaja, yajá, yajé, yojé | Malpighiaceae | Colombian Amazon (Putumayo, Vaupés, Caquetá) / Siona, Kofán, Inga, Coreguaje | dried bark of liana, whole matured stem, root | decoction, snuff | inhalation, chewed, drink | spiritual purification substance dependence emotional imbalance illness diagnosis social/ceremonial use | (Amaris-Álvarez et al., 2021; Gómez, 1989; Rivier and Lindgren, 1972; Schultes and Raffauf, 1992; Trujillo and Correa, 2010) |
| *Brugmansia arborea* (L.) Sweet | borrachera, borrachero, borrachero blanco, cacao sabanero, chamico, corneta, floripón, floripondio, guamuco, guanto, tonca, tonga, yopa | Solanaceae | Boyacá, Andes / Rural community of Sogamoso | leaves, flower, stem | decoction, paste | bath, drink, topical poultice | rheumatism  arthritis  wounds  snakebites social/ceremonial use | (Rueda and Torres, 2017) |
| *Brugmansia aurea* Lagerh. | amarón, borrachera, borrachero, borrachero amarillo, borrachero blanco, cacao sabanero, culebra borrachera, culebra borrachero, munchira, culebra sabanera | Solanaceae | Putumayo / Kofan, Siona, Secoya  Caquetá / Coreguaje, Witoto  Amazonas / Bora, Miraña, Witoto  Vaupés / Tukanoan groups (Desana, Barasana, Cubeo)  Guaviare / Tikuna–Witoto  Llanos Orientales (Orinoquía) / Sikuani, Cuiba | seeds, leaves (34) | infusion, decoction, paste | bath, poultice, oral ingestion | rheumatism  rheumatic pain  fever  muscle spasm  gastric motility issues social/ceremonial use | (Lockwood, 1979; Schultes and Raffauf, 1992) |
| *Brugmansia × candida* Pers. | amarón borrachero, biangan borrachero, borrachero, borrachero blanco, buiesh-borrachero, cacao sabanero, culebra borrachera, floripondio, gumsian borrachero, kinde borrachero, munchira, muscay borrachero, muscuai borrachera, ngntian-borrachero, quinde borrachera, salamán borrachero, salvaje borrachera | Solanaceae | Sibundoy Valley, Putumayo Department, southern Colombia / Inga, Kamsá | leaves | decoction, paste | external application, poultice, oral ingestion | rheumatism  rheumatic pain  arthritis  fatigue/malaiseinsomnia  spirit communication ceremonial use | (Gómez, 1989; Schultes, 1970; Schultes and Raffauf, 1992) |
| *Brugmansia sanguinea* (Ruiz & Pav.) D.Don | borrachero, borrachero colorado, borrachero rojo, borrachero rosado, campanilla, campanilla encarnada, floripón, floripondio, floripondio colorado, floripondio encarnado, floripondio rojo, guamuca, guamuca borrachera, guamuco, guanto, huamuca, huamuco, poroporo, tonga, yopa, tonga y floripondio encarnado, guandué | Solanaceae | Boyacá (Andes) / Rural farming community of Sogamoso and various other  Nariño, Cauca, Cundinamarca, Boyacá / Pasto; Quillacinga, Misak | leaves (8,36), seeds (34), poultice of flowers (36), the whole plant (8) | decoction, infusion | bath, drink the juice, oral ingestion | pain  inflammation  infection  spirit communication  spiritual rituals social/ceremonial use | (García Barriga, 1974; Lockwood, 1979; Rueda and Torres, 2017) |
| *Brunfelsia grandiflora* D.Don | chirigüey, francesi francesino, jardín de mayo, jazmín lavanda, juanramos, pino blanco, sietecueros, sirigüé, chiriguey, chiricaspi | Solanaceae | NW Amazon (Putumayo, Caquetá, Vaupés, Amazonas); Colombia–Ecuador border (Napo–Putumayo) / Kofan; Siona; Secoya; Witoto; Tukanoan groups; Yagua; Shipibo-Conibo; Shuar  Boyacá, Andes / Rural community of San Isidro | bark, root, leaves | drink, wine, tea, paste | oral ingestion, poultice | rheumatism  fever  snakebites  back pain  pain (general)  disease diagnosis  spiritual healing  spirit communication social/ ceremonial use | (González, 2006; Plowman, 1977) |
| *Coriaria ruscifolia* L. | barbasco, chanchí, curtidera, falso helecho, mortiño, reventadera, sancia, sancie, sansá, sansí, sansú, teñidera, tinta, uvilla, zumaque, zeu, mortiño borrachero, tisis, chanchi, mortiño zumaque | Coriariaceae | Cundinamarca and Boyacá, Andes (Páramo region) / Rural communities | leaves and fruits, aerial parts | infusion | oral ingestion | inflammation  digestive complaints  hepatic (liver) complaints  collapse weakness  diarrhea | (Bernal et al., 2011; Peña Lemus, 2020; Rueda and Torres, 2017) |
| *Datura stramonium* L. | carda, chamico, chamico morado, datira, estramonio, herb hedionda, manzano espinoso, nongué, pedro noche, ñongué, ñoque | Solanaceae | Boyacá, Andes / Rural community of Sogamoso | leaves, flower, stem (36) | decoction, infusion | bath, external application, oral ingestion | worm infection  asthma  pain  spirit communication ritual purposes ceremonial use | (Rueda and Torres, 2017) |
| *Dianthera pectoralis* (Jacq.) J.F.Gmel. | amansaguapo, amansaguapos, amansajusticia, amansamacho, cariñito, carpintero, curia, hierba de camarón, insuli madre de yuca, mejora yacayú, herb de san antonio, yakayú, cerebril, tilo criollo, tilo cubano, tilo, tila, ancú, zeb chapantye, chapantye, curía, herb de camarón, técriollo | Acanthaceae | Antioquia, Andes / Rural households and communities (San Cristóbal)  NW Amazon (Amazonas, Caquetá, Putumayo, Vaupés) / Witoto; Bora; Miraña; Tukanoan groups (Desana, Barasana, Cubeo); Coreguaje; Tikuna  Orinoquía (Meta–Vichada) / Sikuani, Cuiba  Caribbean coast / campesino communities  Bogotá / herbal markets | leaves, stem, flower, root, aerial parts | tincture, snuff, infusion, decoction | oral ingestion, inhalation, external application | headaches  muscular pain  cold  prostate problems  menstrual pain  cough  bronchitis  fever  abdominal colic  wounds  ulcers  nervous diseases  insomnia  parasitic infections | (De Smet, 1985; Marín and Gómez, 2015; Ministerio de la Protección Social, 2008; Vera-Marín and Sánchez-Sáen, 2016) |
| *Diplopterys cabrerana* (Cuatrec.) B.Gates | chagro panga, chaguarpanga, yagé, chagropanga | Malpighiaceae | Colombian Amazon (Putumayo, Caquetá, Amazonas, Vaupés, Guaviare; Colombia–Ecuador border (Napo–Putumayo) / Kofan; Siona; Secoya; Witoto; Bora; Miraña; Tukanoan groups (Desana, Barasana, Cubeo); Coreguaje; Tikuna | leaves are added to drink (with water) | decoction | drink, oral ingestion | appetite suppressant, communication with ancestors and spirit world, diagnosis of disease, healing rituals ceremonial use | (Amaris-Álvarez et al., 2021; Gómez, 1989; Rivier and Lindgren, 1972; Schultes and Raffauf, 1992; Trujillo and Correa, 2010) |
| *Drimys granadensis* L.f. | ají, ají de páramo, bocadillo, canela de páramo, canelo, canelo de páramo, canelón, cupis, palo de ají, quijón, quinón, quiñón, quirón | Winteraceae | Cundinamarca, Andes (Páramo region) / Rural communities | bark | infusion, decoction | oral ingestion | gastritis  ulcers  respiratory tract disorders  toothache  dermatitis  rheumatism  fungal infections  yeast infections  bacterial infections  inflammatory diseases  indigestion  scurvy  cancer  wounds (cicatrization) stimulant (general vigor) | (Peña Lemus, 2020) |
| *Erythroxylum coca* Lam. | amarga, coca, coca de danta, coca del sur, coca perua coca propia, coca suave, coca tinga, coca zambico, hayo, hayuelo, ipadá, javo, jayuelo, perua pringamaría, tinga, epadu, patú, pa,too, coca,á, ka,heé, ipatú, huangana coca, pussachpan, mambe | Erythroxylaceae | Amazon/ Siona, Kofán, Inga, Coreguaje, Kamsá, Kubeo, Barasana, Makuna  Sierra Nevada/ Kogi, Arhuaco, Wiwa, Kankuamo, Ika  Andes/ U'wa, Nasa, Yanacona, Muisca  Orinoco/ Sikuani, Cuiba | leaves | snuff, decoction | oral ingestion, inhalation | pain  conjunctivitis  fatigue  appetite suppression  social/ceremonial use  communication and mental clarity  concentration  sensory enhancement | (Arteaga Oliveros, 2018; David, 2015; Granda et al. 2015; Plowman, 1984, 1985; Rojas, 1999; Schultes, 1981) |
| *Galactophora crassifolia* (Müll.Arg.) Woodson | N/A | Apocynaceae | NW Amazon / Ticuna, Siona, Yukuna, Tanimuka, Kubeos, Kofán | latex | latex | external application | uncontrollable head shaking (in children)  epilepsy  trembling  spasms | (Schultes, 1993) |
| *Hyptis brachiata* Briq. | ajicillo, mastranto, jujure, lavaplatos | Lamiaceae | Native habitants of the Orinoco region | leaves | infusion | oral ingestion | nervios | (David, 2015) |
| *Ilex guayusa* Loes. | guayusa, huayusa, aguayusa, guañusa, wayusa. | Aquifoliaceae | Colombian Amazon (border regions) (Putumayo, Caquetá, Amazonas) / Kichwa (Quichua), Siona–Secoya | leaves | tea | oral ingestion | mental clarity  enhanced alertness  energy heightened awareness  pain relief  syphilis  venereal diseases  blood cleansing  digestion  appetite  body strengthening  fertility (women)  post-partum uterine healing  general wellness | (Dueñas et al., 2016; Schultes, 1979a; Schultes and Raffauf, 1992) |
| *Iochroma fuchsioides* (Bonpl.) Miers | corazón de pollo, gallino | Solanaceae | Andean region of Colombia (Boyacá, Cundinamarca) / Campesino/Andean local communities  NW Amazone (Sibundoy) / Kamtsá, Inga | root and leaves | tea | oral ingestion | difficult births  digestive disorders  colic  stomach pain  difficulty digestion  bowel dysfunction  ceremonial/spiritual use | (Schultes and Raffauf, 1992) |
| *Irlbachia nemorosa* (Willd. ex Schult.) Merr. | flor de tierra | Gentianaceae | NW Amazon / Ticuna, Siona, Yukuna, Tanimuka, Kubeos, Kofán | whole plant | decoction | oral ingestion | debilitating forgetfulness  memory loss in elderly | (Schultes, 1993) |
| *Justicia idiogenes* Leonard | N/A | Acanthaceae | NW Amazon / Ticuna, Siona, Yukuna, Tanimuka, Kubeos, Kofán | whole plant | decoction | oral ingestion | trembling | (Schultes, 1993) |
| *Juglans neotropica* Diels | cedro grande, cedro negro, cedro nogal, nogal, nogal bogotano, nogal sabanero | Juglandaceae | Antioquia, Andes / Rural communities of San Cristóbal  Cundinamarca, Andes / Rural farming community of Tabio | leaves, oil, stem | oil, infusion | oral ingestion, external application | migraine  stomach pain  bronchitis  eye infections  ear infections  circulatory system disorders  fever  ulcers  inflammation | (Romero Carrillo, 2019; Rueda and Torres, 2017; Vera-Marín and Sánchez-Sáen, 2016) |
| *Lepechinia bullata* (Kunth) Epling | chirco, sacaojos, salvia negra, salvialugo, selam, tumbué, uva de perro, salvio, salvielugo | Lamiaceae | Boyacá, Andes / Rural community of Sogamoso  Cundinamarca, Andes (Páramo region) / Rural communities | leaves, stem | infusion, decoction, snuff | oral ingestion, inhalation | pain energy/vitality enhancement (tonic)  stimulation/vigo | (Peña Lemus, 2020; Rueda and Torres, 2017) |
| *Lippia alba* (Mill.) N.E.Br. ex Britton & P.Wilson | albahaca de páramo, aliviadolor, chiva, curalotodo, curayá, maiztostado, martinica, oreganito, oreganito de La Sierra, orégano calentano, orégano de cerro, prontoalivio, verbe pronto alivio, menta america quitadolor, toronjil americano | Verbenaceae | Boyacá, Andes / Rural communities of Campo Hermoso and Zetaquira and San Isidro  Tolima, Andes / Pijao indigenous community  Caribbean region / Afro-Colombian communities | aerial parts, leaves | infusion, decoction | oral ingestion | general pain  migrain  digestive complaints  diarrhea  colic  cough  asthma  stomach ailments  tiredness/weakness  flu | (Cadena-González et al., 2013; González, 2006; Mendoza Hernandez et al., 2021; Puyo Anacona, 2018; Ministerio de la Protección Social, 2008) |
| *Mandevilla steyermarkii* Woodson | NA | Apocynaceae | NW Amazon / Ticuna, Siona, Yukuna, Tanimuka, Kubeos, Kofán | root | decoction | oral ingestion | senile dementia  age-related weakness  general weakness in elderly | (Schultes, 1993) |
| *Mimosa albida* Humb. & Bonpl. ex Willd. | zarza, zarza de vega | Fabaceae | Antioquia, Andes / Rural households | leaves, stems, flower | not specified | not specified | sleep disorder insomnia | (Marín and Gómez, 2015) |
| *Myrcianthes leucoxyla* (Ortega) McVaugh | arrayán, arrayán grande, guayabito, guayabito liso, guayabo de Castilla, guayabo liso, guayabón, mortiño, palo blanco, levaduro, mirto | Myrtaceae | Boyacá, Andes / Rural community of Sogamoso  Cundinamarca, Andes (Páramo region) / Rural communities | leaves, fruits | decoction | poultice, chewed | molar pain/toothache  spasms  fever | (Lagos-López, 2007; Peña Lemus, 2020; Rueda and Torres, 2017) |
| *Nicotiana tabacum* L. | tabaco, tabaco calzao, tabaco cuba, tabaco de puntas, tabaco frenteloro, tabaco habano, tabaco negrolindo, tabaco puntalargo, tabak, tawaku, ambil | Solanaceae | Boyacá, Andes / Rural community of Sogamoso  Colombian Amazon (Vaupés, Putumayo, Caquetá, Amazonas) / Tukanoan groups; Witoto; Siona–Secoya; Inga  Andes region/ Muisca  General Colombia / widespread folk medicinal use | leaves, stems | infusion, decoction, snuff | smoked, chewed, enema | malaise/fatigue pain  skin infections  spiritual preparation/initiation  mental clarity  social/ceremonial use  contract/agreement solemnization | (Amaris-Álvarez et al., 2021; David, 2015; De Smet, 1985; Rojas, 1999; Rueda and Torres, 2017) |
| *Ocimum campechianum* Mill. | abaca, albahaca, albahaca blanca, albahaca canela, albahaca de gallinaza, albahaca morada, albahaca toronjil, aruwalo, basil, gallinazo, huo-ca, toronjil | Lamiaceae | Tolima, Andes / Pijao indigenous community | Not specified | not specified | not specified | headaches  rheumatism  feve  inflammation | (Puyo Anacona, 2018) |
| *Passiflora edulis* Sims | curubo, maracuyá, maracuyá de monte, maracuyá silvestre, pachita amarilla, pasiflora | Passifloraceae | Antioquia, Andes / Rural communities of San Cristóbal  Tolima, Andes / Pijao indigenous community | leaves, fruits, stem | infusion, juice | oral ingestion, | nervousness  insomnia / sleep disorders  migraine  stress  restlessness | (Mendoza Hernandez et al., 2021; Ministerio de la Protección Social, 2008; Vera-Marín and Sánchez-Sáen, 2016) |
| *Paullinia yoco* R.E.Schult. & Killip | turuca yoco, yagé-yoco, yo'co, yoco (Inga), yocó | Sapindaceae | Colombian Amazon (Putumayo) / Inga, Siona, Kofán | bark is used from wild lianas | beverage | oral ingestion | hunger suppression  muscular fatigue  malaria  mental clarity/guidance  general vigor  ceremonial use | (Schultes, 1942, 1990) |
| *Psidium guajava* L. | guayaba, guayaba común, guayaba dulce, guayaba manza guayaba pera, guayabo, guayabo colorado, guayabo dulce, guayabo pera, jujuli, jujulinae, vayavacù | Myrtaceae | Boyacá, Andes / Rural community of San Isidro  Tolima, Andes / Pijao indigenous community  Caribbean coast / Rural communities | leaves (fresh), flower, shoot | infusion, decoction, juice | external application, bath, oral ingestion | nervousness  digestive complaints  diarrhea  anemia  gastrointestinal problems  circulatory issues | (Gómez-Estrada et al., 2011; González, 2006; Mendoza Hernandez et al., 2021; Puyo Anacona, 2018) |
| *Psychotria guianensis (*Aubl.) Clos | N/A | Rubiaceae | Native habitants of the Orinoco region | leaves of the tree | infusion | oral ingestion | relaxant anxiety depression | (David, 2015) |
| *Psychotria viridis* Ruiz & Pav. | chacruna, chagropanga | Rubiaceae | Colombian Amazon (Putumayo, Vaupés, Caquetá) / Siona, Kofán, Inga, Coreguaje  Andes region/ Muisca | leaves are added to drink (with water) | decoction, drink | oral ingestion | general wellbeing spiritual healing  disease diagnosis ceremonial use | (Amaris-Álvarez et al., 2021; Gómez, 1989; Rivier and Lindgren, 1972; Schultes and Raffauf, 1992; Trujillo and Correa, 2010) |
| *Smallanthus pyramidalis* (Triana) H.Rob. | anime, arboloco, camargo, colla, escorzonera, jiquimillo, pauche, treeoco | Asteraceae | Cundinamarca, Andes (Páramo region) / Rural communities | leaves | paste | external application, poultice | rheumatic pain  neuralgic pain  menstrual regulation | (Peña Lemus, 2020) |
| *Souroubea corallina* (Mart.) de Roon | N/A | Marcgraviaceae | Colombian Amazon (Caquetá, Putumayo, Amazonas) / Witoto and other groups not specified | leaves | decoction | oral ingestion | apprehension/ anxiety in elderly  nervousness insomnia  susto  spiritual distress | (Schultes, 1980; Schultes and Raffauf, 1990) |
| *Tabernaemontana heterophylla* Vahl | barbasco, barbascos, cojón, güeva peluda, lechoso | Apocynaceae | NW Amazon / Ticuna, Siona, Yukuna, Tanimuka, Kubeos, Kofán | leaves | tea | oral ingestion | slowness/ lethargy in elderly  forgetfulness/ memory loss in elderly  age-related cognitive decline | (Schultes, 1993) |
| *Unonopsis stipitata* Diels | carguero, carguero negro, vara de pescar negra | Annonaceae | NW Amazon / Ticuna, Siona, Yukuna, Tanimuka, Kubeos, Kofán | leaves | not specified | oral ingestion | speech difficulties in elderly  age-related cognitive/ communication decline | (Schultes, 1993) |
| *Unonopsis veneficiorum* (Mart.) R.E.Fr. | carguero, imbira | Annonaceae | NW Amazon / Ticuna, Siona, Yukuna, Tanimuka, Kubeos, Kofán | leaves, root | not specified | oral ingestion | speech loss/  difficulty speaking in elderly (cognitive decline)  antifertility | (Schultes, 1969, 1993) |
| *Valeriana clematitis* Kunth | N/A | Caprifoliaceae | General Colombia / widespread folk medicinal use | subterranean plant parts (Rhizomes, roots, and stolon) fresh or dried | capsules, syrup | oral ingestion, | anxiety  insomnia  convulsions  sleep disturbances | (García Barriga, 1992; Ministerio de la Protección Social, 2008) |
| *Valeriana scandens* L. | N/A | Caprifoliaceae | Antioquia, Andes / Rural households  General Colombia / widespread folk medicinal use | Root, rhizome | extract, ticture | oral ingestion | insomnia  anxiety  blood circulation problems | (Marín and Gómez, 2015; Ministerio de la Protección Social, 2008) |
| *Virola calophylla* Warb (Schultes) | are-de-yé, falsa sangretoro, harecha, mamita, mamita roja, mamito, reventillo, sangre toro, sangretoro, sangretoro de montaña, tierra amarilla, yaegaseii, yakee, yató | Caprifoliaceae | Colombian Amazon (Vaupés, Apaporis, Putumayo, Leticia–Puerto Nariño region, Colombia–Peru–Brazil border) / Witoto (Huitoto); Bora; Ocaina; Makuna; Cubeo; Tukanoan groups (e.g., Desana, Barasana, Tatuyo) | bark trunk of a tree, resin | snuff, pellets/pills | oral ingestion, inhalation | spiritual communication  disease diagnosis ritualistic healing  prophetic purposes  ringworm/fungal skin infections ceremonial use | (Schultes and Hofmann, 1992; Schultes and Raffauf, 1992; Schultes et al., 1977) |

**References**

Amaris-Álvarez, A. F., Díaz-Rueda, D. M., Chautá-Paéz, C. A., and Soto, G. R. N. (2021). Medicina en comunidad y revitalización de la memoria biocultural en la comunidad Muisca de Sesquilé, Cundinamarca, Colombia. *Etnobiología* 19(2), 14–29.

Angulo C, A. F., Rosero R, R. A., and González Insuasti, M. S. (2012). Ethnobotanical study of medicinal plants used by the inhabitants of the village of Genoy, Municipality of Pasto, Colombia. *Universidad y Salud* 14(2), 168–185.

Arteaga Oliveros, J. C. (2018). Estudio etnobotánico: determinación de categorías y valor general de uso de las plantas más importantes en el resguardo indígena yanacona, veredas Nueva Zelanda, Quebradón, Estrecho y Arauca I, Municipio de San Augustín, Huila. [Dissertation]. [Colombia]: Universidad Surcolombia.

Bernal, H. Y., García, H., and Quevedo, G. (2011). Pautas para el conocimiento, conservación y uso sostenible de las plantas medicinales nativas en Colombia. Estrategia Nacional para la conservación de plantas. Primera edición. Bogotá: Ministerio de Ambiente, Vivienda, y Desarrollo Territorial; Instituto de Investigación de Recursos Biológicos Alexander von Humboldt. Primera Edición.

Cadena-González, A. L., Sørensen, M., and Theilade, I. (2013). Use and valuation of native and introduced medicinal plant species in Campo Hermoso and Zetaquira, Boyacá, Colombia. *Journal of Ethnobiology and Ethnomedicine* 9(1), 23. doi:10.1186/1746‑4269‑9‑23

David, E. H. O. (2015). Usos tradicionales de las plantas de la Orinoquia colombiana. *UGCiencia* 21, 16-37.

De Smet, P. A. (1983). A multidisciplinary overview of intoxicating enema rituals in the western hemisphere. *Journal of Ethnopharmacology* 9(2– 3), 129–166. doi:10.1016/0378‑8741(83)90031‑4

De Smet, P. A. (1985). A multidisciplinary overview of intoxicating snuff rituals in the western hemisphere. *Journal of Ethnopharmacology* 13(1), 3–49.

Dueñas, J. F., Jarrett, C., Cummins, I., Logan–Hines, E. (2016). Amazonian Guayusa (*Ilex guayusa* Loes.): A historical and ethnobotanical overview. *Economic Botany* 70(1), 85–91. doi:10.1007/s12231‑016‑9334‑2

García Barriga, H. (1974). Flora medicinal de Colombia: botánica médica. 1a. ed. Bogotá: Tercer Mundo Editores

García Barriga, H. (1992). Flora medicinal de Colombia: botánica médica. 2a. ed. Bogotá: Tercer Mundo Editores

Giraldo Quintero, S. E., Bernal Lizarazú, M. C., Morales Robayo, A., Pardo Lobo, A. Z., and Gamba Molano, L. (2015). Descripción del uso tradicional de plantas medicinales en mercados populares de Bogotá, DC. *NOVA: Publicación Científica en Ciencias Biomédicas* 13(23), 73-80. doi:10.22490/24629448.1707

Gómez, F. O. (1989). Botánica médica Guahibo. Plantas medicinales, mágicas y psicotrópicas utilizadas por los Sikuani y Cuiba (Llanos orientales de Colombia). *Caldasia*, 16(76), 14–22.

Gómez-Estrada, H., Díaz-Castillo, F., Franco-Ospina, L., Mercado-Camargo, J., Guzmán-Ledezma, J., Medina, J. D., and Gaitán-Ibarra, R. (2011). Folk medicine in the northern coast of Colombia: an overview. *Journal of Ethnobiology and Ethnomedicine* 7(1), 27. doi:10.1186/1746‑4269‑7‑27

González, J. Y. T. (2006). Uso tradicional de plantas medicinales en la vereda San Isidro, municipio de San José de Pare-Boyacá: un estudio preliminar usando técnicas cuantitativas. *Acta Biológica Colombiana* 11(2), 137–146.

Granda, L., Rosero, M., and Rosero, A. (2015). “Plantas medicinales de la región Andina tropical. Quinoa (Chenopodium quinoa Willd.) y coca (Erythroxylum sp.), tesoros milenarios para tratamiento medicinal,” in *Etnobotánica y fitoterapia en América*, ed. M. Horák (Brno: Mendel University in Brno),72. doi:10.11118/978-80-7509-349-3-0072

Lagos-López, M. I. (2007). Estudio etnobotánico de especies vegetales con propiedades medicinales en seis municipios de Boyacá, Colombia. *Actualidades Biológicas* 29(86), 87–96.

Lockwood, T. E. (1979). The ethnobotany of Brugmansia. Journal of Ethnopharmacology 1(2), 147–164.

Marín, B. V., and Gómez, R. d. J. F. (2015). Nota Científica: Composición florística y diversidad de las plantas usadas como medicinales en algunos huertos del altiplano del Oriente Antioqueño, Colombia. Etnobiología 13(3), 80–95.

Peña Lemus, L.J. (2020). Etnofarmacología de la flora nativa medicinal del complejo de Páramos de Guerrero, Cundinamarca. Bachelor's thesis, Facultad de Ciencias, Pontificia Universidad Javeriana, Bogotá, Colombia.

Mendoza Hernandez, A. H., Niño Hernández, M. Á., Chaloupková, P., and Fernández-Cusimamani, E. (2021). Estudio etnobotánico del uso de las plantas medicinales en la comunidad indígena Pijao en Natagaima, Colombia. *Boletín Latinoamericano y del Caribe de Plantas Medicinales y Aromáticas* 20(5), 482–495. doi:10.37360/blacpma.21.20.5.35

Ministerio de la Protección Social (2008). *Vademécum Colombiano de Plantas Medicinales*. Bogotá: Imprenta Nacional de Colombia.

Ortiz Gómez, F. (1989). Botánica médica Guahibo. Plantas medicinales, mágicas y psicotrópicas utilizadas por los Sikuani y Cuiba (Llanos orientales de Colombia). *Caldasia* 16(76) 14–22.

Plowman, T. (1977). Brunfelsia in ethnomedicine. *Botanical Museum Leaflets*, *Harvard University* 25(10), 289–320.

Plowman, T. (1984). “The ethnobotany of coca (Erythroxylum spp., Erythroxylaceae)” in *Ethnobotany in the Neotropics: Proceedings*, Advances in Economic Botany, Vol. 1, eds. G. T. Prance and J. A. Kallunki (New York: New York Botanical Garden Press), 1, 62–111.

Plowman, T. (1985). Coca chewing and the botanical origins of coca (Erythroxylum SSPP.) in South America. *Cultural Survival Quarterly* 9(4), 20–25.

Puyo Anacona, C. M. (2018). La etnobotánica un legado ancestral, que debe ser recuperada en beneficio de las nuevas generaciones. Bachelor's thesis, Universidad Militar Nueva Granada, Bogotá, Colombia.

Rivier, L., and Lindgren, J. E. (1972). “Ayahuasca,” the South American hallucinogenic drink: An ethnobotanical and chemical investigation. *Economic Botany* 26(2), 101–129. doi:10.1007/BF02860772

Rojas, J. D. G. (1999). Etnobotánica de las adicciones y cultura democrática. Revista Colombiana de psicología(8), 30–37.

Romero Carrillo, A. M. (2019). Conservación de Usos de Plantas Nativas reportados por la comunidad Campesina en Tabio, Cundinamarca. Bachelor's thesis, Departamento de Ciencias Biológicas, Universidad de Los Andes, Bogotá, Colombia.

Rueda, M. G., and Torres, M. T. (2017). Etnobotánica y usos de las plantas de la comunidad rural de Sogamoso, Boyacá, Colombia. *Revista Iberoamericana Ambiente & Sustentabilidad* 8(2), 187–206.

Schultes, R. E. (1942). Plantae Colombianae II. *Botanical Museum Leaflets, Harvard University* 10(10), 301–324.

Schultes, R. E. (1969). De plantis toxicariis e mundo novo tropicale commentationes IV. *Botanical Museum Leaflets, Harvard University* 22(4), 133–164.

Schultes, R. E. (1970). The botanical and chemical distribution of hallucinogens. *Annual Review of Plant Physiology* 21(1), 571–598. doi:10.1146/annurev.pp.21.060170.003035

Schultes, R. E. (1979a). Discovery of an ancient guayusa plantation in Colombia. *Botanical Museum Leaflets, Harvard University* 27(5/6), 143–153.

Schultes, R. E. (1979b). Evolution of the identification of the major South American narcotic plants. *Journal of Psychedelic Drugs* 11(1-2), 119–134. doi:10.1080/02791072.1979.10472105

Schultes, R. E. (1980). De plantis toxicariis e Mundo Novo tropicale commentationes XXIX. A suspected new Amazonian hallucinogen. *Botanical Museum Leaflets, Harvard University* 28(3), 271–275.

Schultes, R. E. (1981). Coca in the northwest Amazon. *Journal of Ethnopharmacology* 3, 173–194. doi:10.1016/0378-8741(81)90053-2

Schultes, R. E., and Raffauf, R. F. (1990). *The healing forest: medicinal and toxic plants of the Northwest Amazonia*. Portland: Dioscorides Press.

Schultes, R. E. (1993). Plants in treating senile dementia in the Northwest Amazon. Journal of Ethnopharmacology 38(2–3), 121–128. doi:10.1016/0378-8741(93)90013-R

Schultes, R. E., and Hofmann, A. (1992). *Plants of the gods: origins of hallucinogenic use*. New York: McGraw-Hill.

Schultes, R. E., and Raffauf, R. F. (1992). *Vine of the soul: medicine men, their plants and rituals in the Colombian Amazonia*. Oracle: Synergetic Press Inc.

Schultes, R. E., Swain, T., and Plowman, T. C. (1977). De Plantis Toxicariis E Mundo Novo Tropicale Commentationes XVII: Virola as an oral hallucinogen among the Boras of Peru. *Botanical Museum Leaflets, Harvard University* 25, 259–272.

Trujillo, W., and Correa, M. (2010). Plantas usadas por una comunidad indígena coreguaje en la amazonía colombiana. *Caldasia* 32, 1–20.

Vera-Marín, B., and Sánchez-Sáen, M. (2016). Plantas medicinales y predictibilidad de uso en algunas veredas del corregimiento de San Cristóbal (Antioquia), Colombia. *Actualidades Biológicas* 38, 167–180.
