# Supplementary material for: Exploring Colombian medicinal flora used in indigenous and campesino health systems for neuropsychiatric disorders and neuropharmacological potential: an ethnopharmacological review
Source: Front Pharmacol. 2026 Mar 11;17:1729887. doi: 10.3389/fphar.2026.1729887 (PMC13013478; doi:10.3389/fphar.2026.1729887)
Supplement: Supplementary file 2 [file Table2.docx]

**Supplementary Table 2.** **Forty-two identified species grouped by primary CNS-related effects.** Categories represent a hybrid classification that integrates traditional use terminology with biomedical, psychiatric, and neurological terminology. Traditional indications were interpreted through a biomedical lens to facilitate pharmacological comparison; indigenous diagnostic frameworks may differ substantially from these categories. Vernacular names obtained from the Annotated checklist of useful plants of Colombia (Diazgranados, et al. 2022a). Detailed information about the ethnobotanical information (including sources), can be consulted in Supplementary Table 1.

| **Main Effect** | **Plant** | **Vernacular name(s)** |
| --- | --- | --- |
| **Hallucinogenic** | *Banisteriopsis caapi* (Spruce ex Griseb.) Morton | ayahuasca, bejuco bravo, bejuco de oro, bejuco dulce, caapi, caji, caji-idirecaii, capi, capi o caapi, changropanga, cordón umbilical, dapa, kapi, liana del napo, natema, oofa, pejí, pildé, pinde, yaco borrachero, yagé, yagé de cacería, yagé de monte, yaja, yajá, yajé, yojé |
|  | *Psychotria viridis* Ruiz & Pav. | chacruna, chagropanga |
|  | *Diplopterys cabrerana* (Cuatrec.) B.Gates | chagro panga, chaguarpanga, yagé, chagropanga |
|  | *Brugmansia × candida*Pers*.* | amarón borrachero, biangan borrachero, borrachero, borrachero blanco, buiesh-borrachero, cacao sabanero, culebra borrachera, floripondio, gumsian borrachero, kinde borrachero, munchira, muscay borrachero, muscuai borrachera, ngntian-borrachero, quinde borrachera, salamán borrachero, salvaje borrachera |
|  | *Brugmansia aurea*Lagerh*.* | amarón, borrachera, borrachero, borrachero amarillo, borrachero blanco, culebra borrachera/o, culebra sabanera, munchira |
|  | *Brugmansia arborea*(L.) Sweet | borrachera, borrachero, borrachero blanco, cacao sabanero, chamico, corneta, floripón, floripondio, guamuco, guanto, tonca, tonga, yopa |
|  | *Brugmansia sanguinea*(Ruiz & Pav.) D.Don | borrachero, borrachero colorado, borrachero rojo, borrachero rosado, campanilla, campanilla encarnada, floripón, floripondio, floripondio colorado, floripondio encarnado, floripondio rojo, guamuca/o, guamuca borrachera, guandué, guanto, huamuca/o, poroporo, tonga, tonga y floripondio encarnado, yopa |
|  | *Brunfelsia grandiflora*D.Don | chiricaspi, chirigüey, chiriguey, francesi, francesino, jardín de mayo, jazmín lavanda, juanramos, pino blanco, sietecueros, sirigüe |
|  | *Iochroma fuchsioides*(Bonpl.) Miers | corazón de pollo, gallino |
|  | *Virola calophylla*Warb (Schultes) | are-de-yé, falsa sangretoro, harecha, mamita, mamita roja, mamito, reventillo, sangre toro, sangretoro, sangretoro de montaña, tierra amarilla, yaegaseii, yakee, yató |
|  | *Datura stramonium*L. | carda, chamico, chamico morado, datira, estramonio, herb hedionda, manzano espinoso, nongué, pedro noche, ñongué, ñoque |
|  | *Anadenanthera peregrina*(L.) Speg. | carripaco, cohoba, cojoba, dopa, dopamagerná, dopanae (Sikuani), dopane (Cuiba), guahibo, lomo de caimán, niopo, yompa, yopa, yopo, yoto, yumpa, yupa |
| **Tranquilizing** | *Souroubea corallina* (Mart.) de Roon | NA |
|  | *Psychotria guianensis* (Aubl.) Clos | NA |
|  | *Ocimum campechianum*Mill*.* | abaca, albahaca, albahaca blanca, albahaca canela, albahaca de gallinaza, albahaca morada, albahaca toronjil, aruwalo, basil, gallinazo, huo-ca, toronjil |
|  | *Aloysia citrodora*Paláu | alegría, cedrón, cidrón, luisa, luisa de Chile, lemon verbe, saca ojo, yerbaluisa yerba luisa,s zorrillo |
|  | *Dianthera pectoralis*(Jacq.) J.F.Gmel. | amansaguapo, amansaguapos, amansajusticia, amansamacho, ancú, cariñito, carpintero, chapantye, cerebril, curia, curía, herb de San Antonio, herb de camarón, hierba de camarón, insuli madre de yuca, mejora yacayú, técriollo, tilo criollo, tilo cubano, tilo/a, yakayú, zeb chapantye |
|  | *Psidium guajava*L*.* | guayaba, guayaba común, guayaba dulce, guayaba manza guayaba pera, guayabo, guayabo colorado, guayabo dulce, guayabo pera, jujuli, jujulinae, vayavacù |
| **Anxiolytic** | *Hyptis brachiata*Briq*.* | ajicillo, jujure, mastranto, lavaplatos |
|  | *Valeriana clematitis*Kunth | NA |
|  | *Passiflora edulis*Sims | curubo, maracuyá, maracuyá de monte, maracuyá silvestre, pachita amarilla, pasiflora |
| **Stimulant** | *Paullinia yoco*R.E.Schult. & Killip | turuca yoco, yagé-yoco, yo'co, yoco (Inga), yocó |
|  | *Ilex guayusa*Loes*.* | aguayusa, guañusa, guayusa, huayusa, wayusa |
|  | *Coriaria ruscifolia*L*.* | barbasco, chanchí, chanchi, curtidera, falso helecho, mortiño, mortiño borrachero, mortiño zumaque, reventadera, sancia, sancie, sansá, sansí, sansú, teñidera, tinta, tisis, uvilla, zumaque, zeu |
|  | *Erythroxylum coca*Lam. | amarga, coca, coca.á, coca de danta, coca del sur, coca perua, coca propia, coca suave, coca tinga, coca zambico, epadu, hayo, hayuelo, huangana coca, ipadá, ipatú, javo, jayuelo, ka,heé, mambe, perua pringamaría, patú, pa,too, pussachpan, tinga |
|  | *Nicotiana tabacum*L. | ambil, tabaco, tabaco calzao, tabaco cuba, tabaco de puntas, tabaco frenteloro, tabaco habano, tabaco negrolindo, tabaco puntalargo, tabak, tawaku |
|  | *Drimys granadensis*L.f. | ají, ají de páramo, bocadillo, canela de páramo, canelo, canelo de páramo, canelón, cupis, palo de ají, quijón, quinón, quiñón, quirón |
|  | *Lepechinia bullata*(Kunth) Epling | chirco, sacaojos, salvia negra, salvialugo, salvio, selam, tumbué, uva de perro |
| **Anticonvulsive** | *Galactophora crassifolia* (Müll.Arg.) Woodson | NA |
|  | *Justicia idiogenes*Leonard | NA |
| **Sedative** | *Mimosa albida*Humb. & Bonpl. ex Willd. | zarza, zarza de vega |
|  | *Myrcianthes leucoxyla* (Ortega) McVaugh | arrayán, arrayán grande, guayabito, guayabito liso, guayabo de Castilla, guayabo liso, guayabón, levaduro, mirto, mortiño, palo blanco |
|  | *Valeriana scandens*L. | NA |
|  | *Lippia alba*(Mill.) N.E.Br. ex Britton & P.Wilson | albahaca de páramo, aliviadolor, chiva, curalotodo, curayá, maiztostado, martinica, menta america, oreganito, oreganito de la sierra, orégano calentano, orégano de cerro, prontoalivio, quitadolor, toronjil americano, verbe pronto alivio |
| **Dementia** | *Adenocalymma schomburgkii* (DC.) L.G.Lohmann | bejuco mataganado |
|  | *Irlbachia nemorosa* (Willd. ex Schult.) Merr. | flor de tierra |
|  | *Mandevilla Steyermarkii*Woodson | NA |
|  | *Unonopsis veneficiorum*(Mart.) R.E.Fr. | carguero, imbira |
|  | *Unonopsis stipitate*Diels | carguero, carguero negro, vara de pescar negra |
|  | *Tabernaemontana heterophylla* Vahl | barbasco, barbascos, cojón, güeva peluda, lechoso |
| **Alleviate neurological pain** | *Smallanthus pyramidalis* (Triana) H.Rob. | anime, arboloco, camargo, colla, escorzonera, jiquimillo, pauche, treeoco |
|  | *Juglans neotropica*Diels | cedro grande, cedro negro, cedro nogal, nogal, nogal bogotano, nogal sabanero |

Note: Primary effect refers to the most frequently reported or culturally salient CNS-related use; multiple effects may exist for a given species.
