# Supplementary material for: Exploring Colombian medicinal flora used in indigenous and campesino health systems for neuropsychiatric disorders and neuropharmacological potential: an ethnopharmacological review
Source: Front Pharmacol. 2026 Mar 11;17:1729887. doi: 10.3389/fphar.2026.1729887 (PMC13013478; doi:10.3389/fphar.2026.1729887)
Supplement: Supplementary file 3 [file Table3.docx]

**Supplementary Table 3.** **Phytochemical composition and neuropharmacological mechanisms of 42 Colombian medicinal plant species.** The table shows comprehensive information on identified bioactive compounds, molecular targets, mechanisms of action, and clinical/ behavioral effects. "Mechanism Status" indicates: **Verified** = direct experimental evidence from this species (receptor binding, enzyme assays, clinical trials); **Partially verified** = mechanism supported by preclinical studies but lacking clinical validation, or mechanism inferred from isolated compounds; **Hypothesized** = mechanism extrapolated from related species or compound class without direct testing; **Unknown** = no pharmacological studies conducted. The six priority species highlighted in this review are marked in **bold**.

| **Plant Species** | **Major Compounds Identified** | **Compound Class** | **CNS Target(s)** | **Neuropharmacological Mechanism** | **Behavioral/Clinical Effect** | **Mechanism Status** | **References** |
| --- | --- | --- | --- | --- | --- | --- | --- |
| *Adenocalymma schomburgkii* (DC.) L.G.Lohmann | Not characterized | Unknown | Unknown | Unknown | Not studied | Unknown | Ethnobotanical evidence only |
| *Aloysia citrodora* Paláu | Verbascoside, flavonoids (apigenin, luteolin), essential oils (citral, limonene) | Phenylpropanoids, flavonoids, monoterpenes | GABA_A receptor (hypothesized), oxidative stress pathways | Possible GABAergic modulation; anxiolytic activity | Improved sleep quality (PSQI scores), reduced anxiety | Partially verified (clinical effect, mechanism inferred) | Afrasiabian et al., 2019; Martínez-Rodríguez et al., 2022; Perez-Pinero et al., 2024, Rashidian et al., 2016 |
| *Anadenanthera peregrina* (L.) Speg. | Bufotenin (5-OH-DMT), DMT, 5-MeO-DMT | Tryptamine alkaloids | 5-HT2A, 5-HT1A receptors | Serotonin receptor agonism | Hallucinogenic, altered perception | Verified (compound level) | Torres and Rebke, 2006; Carbonaro and Gatch, 2016 |
| *Banisteriopsis caapi* (Spruce ex Griseb.) Morton | β-carbolines: harmine, harmaline, tetrahydroharmine | Alkaloids (β-carbolines) | MAO-A, 5-HT2A, σ1 receptors | Reversible MAO-A inhibition; serotonin receptor modulation | Antidepressant, anxiolytic, hallucinogenic, neuroplasticity enhancement | Verified | Dos Santos et al., 2016; Palhano-Fontes et al., 2019; Glennon et al., 2000 |
| *Brugmansia arborea* (L.) Sweet | Scopolamine, hyoscyamine, atropine | Tropane alkaloids | Muscarinic (M1–M5) receptors | Competitive muscarinic antagonism (anticholinergic) | Delirium, hallucinations, amnesia, sedation | Verified (compound level) | Klinkenberg and Blokland, 2010; Renner et al., 2005 |
| *Brugmansia aurea* Lagerh. | Scopolamine, hyoscyamine | Tropane alkaloids | Muscarinic receptors | Anticholinergic | Hallucinogenic, delirium | Verified (compound level) | Klinkenberg and Blokland, 2010; Renner et al., 2005 |
| *Brugmansia × candida* Pers. | Scopolamine, hyoscyamine | Tropane alkaloids | Muscarinic receptors | Anticholinergic | Hallucinogenic, delirium | Verified (compound level) | Klinkenberg and Blokland, 2010; Renner et al., 2005 |
| *Brugmansia sanguinea* (Ruiz & Pav.) D.Don | Scopolamine, hyoscyamine | Tropane alkaloids | Muscarinic receptors | Anticholinergic | Hallucinogenic, sedation | Verified (compound level) | Klinkenberg and Blokland, 2010; Renner et al., 2005 |
| ***Brunfelsia grandiflora* D.Don** | **Scopoletin, phytol, mannitol** | **Coumarin, diterpene alcohol** | **GABA_A receptor (phytol); antioxidant pathways** | **GABAergic enhancement; neuroprotection against oxidative stress** | **Anticonvulsant, cytoprotective on neuron-like cells** | **Verified (compound level)** | **Costa et al. 2012; Rodríguez et al. 2023** |
| *Coriaria ruscifolia* L. | Coriamyrtin, tutin | Sesquiterpene lactones | GABA_A receptor (antagonist) | GABA_A receptor antagonism (convulsant) | Convulsions, stimulation (toxic) | Verified (toxicity) | Fuentealba et al., 2007; Popat et al., 2001 |
| *Datura stramonium* L. | Scopolamine, hyoscyamine, atropine | Tropane alkaloids | Muscarinic receptors | Anticholinergic antagonism | Delirium, hallucinations, amnesia, anesthesia | Verified (compound level) | Klinkenberg and Blokland, 2010; Renner et al., 2005 |
| ***Dianthera pectoralis* (Jacq.) J.F.Gmel.** | **Umbelliferone, coumarin derivatives** | **Coumarins** | **GABA_A receptor; monoamine systems (DA, NE, 5-HT, DOPAC, HVA in brain)** | **GABAergic modulation; decreased monoamine levels** | **Anxiolytic, sedative, anti-aggressive, anticonvulsant** | **Verified** | **Awad et al., 2009; Venâncio et al., 2011; Chanfrau et al., 2013** |
| *Diplopterys cabrerana* (Cuatrec.) B.Gates | DMT (N,N-dimethyltryptamine) | Tryptamine alkaloid | 5-HT2A, 5-HT2C, 5-HT1A receptors | Serotonin receptor agonism | Hallucinogenic, antidepressant (in ayahuasca context) | Verified | Carbonaro and Gatch, 2016 |
| *Drimys granadensis* L.f. | Polygodial, drimenol | Sesquiterpene dialdehydes | TRPA1 channels (pungency) | Sensory irritation; possible CNS stimulation via sensory pathways | Stimulant (possibly via pungent compounds) | Hypothesized | Drieu La Rochelle et al., 2015 |
| *Erythroxylum coca* Lam. | Cocaine, other tropane alkaloids | Tropane alkaloids | DAT, SERT, NET (dopamine, serotonin, norepinephrine transporters) | Monoamine reuptake inhibition | Stimulant, euphoric, appetite suppression, increased alertness | Verified | Restrepo et al., 2019; Sora et al., 2001 |
| *Galactophora crassifolia* (Müll.Arg.) Woodson | Not characterized | Unknown | Unknown | Unknown | Not studied | Unknown | Ethnobotanical evidence only |
| *Hyptis brachiata* Briq. | Essential oils, flavonoids (tentative) | Terpenes, flavonoids | Unknown | Unknown | Not studied | Unknown | Tafurt-Garcia et al. 2014 |
| *Ilex guayusa* Loes. | Caffeine, theobromine, chlorogenic acid, L-theanine | Xanthine alkaloids, amino acids, phenolic acids, triterpenoids | Adenosine receptors (A1, A2A antagonism by caffeine); NMDA, AMPA modulation (L-theanine) | Adenosine antagonism (stimulation); anxiolytic via theanine | Stimulant, increased alertness, possible anxiolytic effect | Verified (compound level) | Sequeda-Castañeda et al., 2016 |
| ***Iochroma fuchsioides* (Bonpl.) Miers** | **Withanolides (withanolide D)** | **Steroidal lactones** | **Unknown (neurite outgrowth pathways hypothesized)** | **Hypothesized promotion of neurite regeneration, dendritic formation** | **Hypothesized neuroprotective, neurogenic; potential for neurodegenerative diseases** | **Hypothesized** | **Raffauf et al. 1991; Hannan et al. 2020; Zhao et al. 2002, Mirjalili et al., 2009** |
| *Irlbachia nemorosa* (Willd. ex Schult.) Merr. | Not characterized | Unknown | Unknown | Unknown | Not studied | Unknown | Ethnobotanical evidence only |
| *Justicia idiogenes* Leonard | Juglone, tannins, flavonoids | Naphthoquinones, polyphenols | Unknown (possibly anti-inflammatory, COX inhibition) | Possibly anti-inflammatory; analgesic | Migraine relief, pain reduction | Hypothesized | Hurtado-Manrique et al., 2010; Zakavi et al., 2021 |
| *Juglans neotropica* Diels | Not characterized | Unknown | Unknown | Unknown | Not studied | Unknown | Ethnobotanical evidence only |
| *Lepechinia bullata* (Kunth) Epling | Essential oils (unspecified) | Terpenes | Unknown | Unknown | Not studied | Unknown | Jonathan et al., 1989 |
| *Lippia alba* (Mill.) N.E.Br. ex Britton & P.Wilson | Citral, carvone, linalool, myrcene | Monoterpenes | GABA_A receptor; voltage-gated Ca2+ channels | GABAergic modulation; calcium channel blockade (spasmolytic) | Sedative, anxiolytic, anticonvulsant, analgesic (migraine) | Verified | Conde et al., 2011; Hennebelle et al., 2008 |
| *Mandevilla steyermarkii* Woodson | Not characterized | Unknown (Apocynaceae typically alkaloid-rich) | Unknown | Unknown | Not studied | Unknown | Ethnobotanical evidence only |
| *Mimosa albida* Humb. & Bonpl. ex Willd. | Flavonoids, tannins (tentative) | Polyphenols | Unknown | Unknown | Antinociceptive | Unknown | Abad, 2022 |
| *Myrcianthes leucoxyla* (Ortega) McVaugh | Essential oils, flavonoids (tentative) | Terpenes, polyphenols | Unknown | Unknown | Not studied | Unknown | Granados et al. 2014 |
| *Nicotiana tabacum* L. | Nicotine, nornicotine, anabasine | Alkaloids (pyrrolidines) | Nicotinic acetylcholine receptors (α4β2, α7 nAChR) | nAChR agonism; increased DA, NE, ACh release | Stimulant, cognitive enhancement, analgesia, addiction | Verified |  |
| *Ocimum campechianum* Mill. | Essential oils (eugenol, linalool), flavonoids | Phenylpropanoids, terpenes | Unknown | Unknown | Not studied | Hypothesized | Ricarte et al., 2020 |
| *Passiflora edulis* Sims | Flavonoids (apigenin, chrysin, vitexin), harman alkaloids, GABA | Flavonoids, β-carbolines, amino acids | GABA_A receptor (benzodiazepine site); MAO inhibition (harman) | Positive allosteric modulation of GABA_A; mild MAO inhibition | Anxiolytic, sedative, improved sleep | Hypothesized from genus-level evidence | He et al. 2020; Janda et al. 2020 (*P. incarnata*) |
| *Paullinia yoco* R.E.Schult. & Killip | Caffeine, theobromine | Xanthine alkaloids | Adenosine receptors (A1, A2A antagonism) | Adenosine antagonism | Stimulant, increased alertness, appetite suppression | Verified (compound level) | Weckerle et al., 2003 |
| ***Psidium guajava* L.** | **Quercetin, gallic acid, catechins, guajaverin** | **Flavonoids, phenolic acids** | **AChE inhibition; Nrf2 antioxidant pathway; GABA_A (hypothesized)** | **Acetylcholinesterase inhibition; ROS scavenging; possible GABAergic** | **Anxiolytic, antidepressant, sedative, analgesic; potential cognitive enhancement** | **Verified (preclinical)** | **Lorena et al. 2022; Biswas et al. 2021; Shaheen et al. 2000** |
| *Psychotria guianensis (*Aubl.) Clos | Alkaloids (unspecified) | Alkaloids | Unknown | Unknown | Not studied | Unknown | Klein-Júnior et al. 2016 |
| *Psychotria viridis* Ruiz & Pav. | DMT (N,N-dimethyltryptamine) | Tryptamine alkaloid | 5-HT2A, 5-HT2C, 5-HT1A receptors | Serotonin receptor agonism | Hallucinogenic, antidepressant, anxiolytic (in ayahuasca) | Verified | Carbonaro and Gatch, 2016 |
| *Smallanthus pyramidalis* (Triana) H.Rob. | Sesquiterpene lactones, diterpenes | Terpenes | Unknown (possibly anti-inflammatory) | Hypothesized anti-inflammatory | Analgesic (neurological pain) | Hypothesized | Guzmán Avendaño and Barrera Adame, 2011 |
| ***Souroubea corallina* (Mart.) de Roon** | **betulinic acid (related species) contain betulinic acid** | **Triterpenes (hypothesized)** | **GABA_A receptor (hypothesized from genus)** | **Hypothesized GABAergic modulation from related species** | **Hypothesized anxiolytic, antidepressant** | **Hypothesized** | **Puniani et al. 2015** |
| ***Tabernaemontana heterophylla* Vahl** | **Voacangine, coronaridine, 19-heyneanine, vobasine, affinisine, olivacine** | **Indole alkaloids** | **GABA_A receptor; AChE; multiple CNS receptors** | **GABAergic modulation (coronaridine); AChE inhibition (voacangine); multi-target effects** | **Sedative, anxiolytic, anticonvulsant; potential cognitive benefits** | **Partially verified** | **McKenna et al. 2011; Arias et al. 2023; Andrade et al. 2005, Boligon et al., 2012** |
| *Unonopsis stipitata* Diels | Not characterized | Unknown (Annonaceae acetogenins, alkaloids) | Unknown | Unknown | Not studied | Unknown | Ethnobotanical evidence only |
| *Unonopsis veneficiorum* (Mart.) R.E.Fr. | Not characterized | Unknown (Annonaceae family) | Unknown | Unknown | Not studied | Unknown | Ethnobotanical evidence only |
| *Valeriana clematitis* Kunth | Valepotriates, valerenic acid, isovaleric acid | Iridoid esters, sesquiterpene acids | GABA_A receptor; adenosine A1 receptor | GABAergic enhancement; possible adenosine modulation | Anxiolytic, sedative, anticonvulsant | Partially verified (genus level) | Yuan et al. 2004; Celis et al. 2007 |
| *Valeriana scandens* L. | Not characterized (Valeriana genus valepotriates) | Unknown (likely iridoids) | GABA_A receptor (hypothesized from genus) | Hypothesized GABAergic | Hypothesized sedative, anxiolytic | Hypothesized from genus-level evidence | Yuan et al. 2004 |
| *Virola calophylla* Warb (Schultes) | 5-MeO-DMT, DMT, β-carbolines (including 6-methoxy-tetrahydro-β-carboline) | Tryptamine alkaloids, β-carbolines | 5-HT2A, 5-HT1A receptors; MAO-A (β-carbolines) | Serotonin receptor agonism; MAO inhibition | Hallucinogenic, altered perception, visionary effects | Verified (compound level) | Schultes and Holmstedt, 1968; Reckweg et al., 2021 |

AChE = acetylcholinesterase; GABA = γ-aminobutyric acid; DA = dopamine; NE = norepinephrine; 5-HT = serotonin; DMT = N,N-dimethyltryptamine; MAO = monoamine oxidase; DAT = dopamine transporter; SERT = serotonin transporter; NET = norepinephrine transporter; nAChR = nicotinic acetylcholine receptor; DOPAC = 3,4-dihydroxyphenylacetic acid; HVA = homovanillic acid.

**References**

Abad, J. P. D. (2022). Estudio químico y evaluación de la actividad antimicrobiana de *Mimosa albida* vergonzosa.

Afrasiabian, F., Mirabzadeh Ardakani, M., Rahmani, K., Azadi, N. A., Alemohammad, Z. B., Bidaki, R., et al. (2019). *Aloysia citriodora* Palau (lemon verbena) for insomnia patients: a randomized, double-blind, placebo-controlled clinical trial of efficacy and safety. *Phytother Res*, 33(2):350-359. doi:10.1002/ptr.6228

Arias, H. R., De Deurwaerdere, P., Scholze, P., Sakamoto, S., Hamachi, I., Di Giovanni, G., et al. (2023). Coronaridine congeners induce sedative and anxiolytic-like activity in naive and stressed/anxious mice by allosteric mechanisms involving increased GABA(A) receptor affinity for GABA. *Eur. J. Pharmacol.*, 953:175854. doi:10.1016/j.ejphar.2023.175854

Awad, R., Ahmed, F., Bourbonnais-Spear, N., Mullally, M., Ta, C. A., Tang, A., et al. (2009). Ethnopharmacology of Q'eqchi' Maya antiepileptic and anxiolytic plants: effects on the GABAergic system. *J. Ethnopharmacol.*, 125(2):257–264. doi:10.1016/j.jep.2009.06.034

Boligon, A. A., Schwanz, T. G., Piana, M., Bandeira, R. V., Frohlich, J. K., Brum, T. F. D., et al. (2013). Chemical composition and antioxidant activity of the essential oil of *Tabernaemontana catharinensis* A. DC. leaves. *Nat. Prod. Res.*, 27(1):68–71.

Carbonaro, T. M., Gatch, M. B. (2016). Neuropharmacology of N,N-dimethyltryptamine. *Brain Res. Bull.*, 126:74–88. doi:10.1016/j.brainresbull.2016.04.016

Celis, C. T., Rincón, J., Guerrero, M. F. (2007). Actividad farmacológica sobre el sistema nervioso central del extracto etanólico y de la fracción alcaloidal de *Valeriana pavonii.* *Rev. Colomb. Cienc. Quím-Farm*., 36(1):11–22.

Chanfrau, J. E. R., Ferrada, C. R., Mendoza, A. N. (2013). Obtention of dry extract from aqueous extracts of *Justicia pectoralis* Jacq. (tilo). *Rev. Cuba. Plantas Med*, 18(4).

Conde, R., Corrêa, V. S., Carmona, F., Contini, S. H., Pereira, A. M. (2011). Chemical composition and therapeutic effects of *Lippia alba* (Mill.) NE Brown leaves hydro-alcoholic extract in patients with migraine. *Phytomedicine*, 18(14):1197–1201. doi:10.1016/j.phymed.2011.07.006

Costa, J., Ferreira, P., De Sousa, D., Jordan, J., Freitas, R. (2012). Anticonvulsant effect of phytol in a pilocarpine model in mice. *Neurosci. Lett.*, 523(2):115–118. doi:10.1016/j.neulet.2012.05.011

Dos Santos, R. G., Osorio, F. L., Crippa, J. A., Riba, J., Zuardi, A. W., Hallak, J. E. (2016). Antidepressive, anxiolytic, and antiaddictive effects of ayahuasca, psilocybin and lysergic acid diethylamide (LSD): a systematic review of clinical trials published in the last 25 years. *Ther. Adv. Psychopharmacol.*, 6(3):193–213. doi:10.1177/2045125316638008

Drieu La Rochelle, C., Acebey, L. (2015). Bioactive compounds from Drimys species. *Phytochem. Rev.*, 14(1):129–144. doi:10.1007/s11101-014-9381-2

Fuentealba, J., Guzmán, L., Manríquez-Navarro, P., Pérez, C., Silva, M., Becerra, J., Aguayo, L. G. (2007). Inhibitory effects of tutin on glycine receptors in spinal neurons. *Eur. J. Pharmacol.*, 559(1):61–64.

Glennon, R. A., Dukat, M., Grella, B., Hong, S., Costantino, L., Teitler, M., et al. (2000). Binding of beta-carbolines and related agents at serotonin (5-HT2 and 5-HT1A), dopamine (D2) and benzodiazepine receptors. *Drug Alcohol Depend.*, 60(2):121–132. doi:10.1016/s0376-8716(99)00148-9

Granados, C., Yáñez, X., Acevedo, D. (2014). Evaluación de la actividad antioxidante del aceite esencial foliar de *Myrcianthes leucoxyla* de norte de Santander (Colombia). *Información Tecnológica*, 25(3):11–16.

Guzmán Avendaño, A. J., Barrera Adame, D. A. (2011). Estudio fitoquímico de hojas y flores de *Smallanthus pyramidalis* (Triana) H. Rob. (árboloco) y su uso en la recuperación de los humedales de Bogotá. *Colombia Forestal*, 14(1):41–50.

Hannan, M. A., Dash, R., Haque, M., Choi, S. M., Moon, I. S. (2020). Neuropharmacological actions of *Withania somnifera* in Alzheimer's disease. *CNS Neurol. Disord. Drug Targets*, 19(7):541-556. doi:10.2174/1871527319999200730214807

Hennebelle, T., Sahpaz, S., Joseph, H., Bailleul, F. (2008). Ethnopharmacology of *Lippia alba*. *J. Ethnopharmacol.*, 116(2):211–222. doi:10.1016/j.jep.2007.11.044

He, X., Luan, F., Yang, Y., Wang, Z., Zhao, Z., Fang, J., et al. (2020). *Passiflora edulis*: an insight into current researches on phytochemistry and pharmacology. *Front. Pharmacol.,* 11:617. doi:10.3389/fphar.2020.00617

Hurtado-Manrique, P., Jurado Teixeira, B., Ramos Llica, E., Calixto Cotos, M. (2015). Evaluación de la actividad antioxidante del extracto hidroalcohólico estandarizado de hojas de *Juglans neotropica* Diels (nogal peruano). *Rev. Soc. Quím. Perú.*, 81(3):283–291.

Janda, K., Wojtkowska, K., Jakubczyk, K., Antoniewicz, J., Skonieczna-Żydecka, K. (2020). *Passiflora incarnata* in neuropsychiatric disorders—a systematic review. *Nutrients*, 12(12):3894. doi:10.3390/nu12123894

Jonathan, L. T., Che, C. T., Pezzuto, J. M., Fong, H. H., Farnsworth, N. R. (1989). 7-O-Methylhorminone and other cytotoxic diterpene quinones from *Lepechinia bullata*. J *Nat. Prod.*, 52(3):571–575.

Klinkenberg, I., Blokland, A. (2010). The validity of scopolamine as a pharmacological model for cognitive impairment: a review of animal behavioral studies. *Neurosci. Biobehav. Rev.*, 34(8):1307–1350. doi:10.1016/j.neubiorev.2010.04.001

Klein-Júnior, L. C., Passos, C. D. S., Salton, J., Bitencourt de, F. G., Funez, L., de Andrade, J. P., et al. (2016). Multifunctional monoamine oxidases and cholinesterases inhibitory effects, as well as UPLC-DAD-MS chemical profile of alkaloid fractions obtained from species of the Palicoureeae tribe. *Nat. Prod. Commun.*, 11(9):1343–1348.

Lorena, C., Ressaissi, A., Serralheiro, M. L. (2022). Bioactives from *Psidium guajava* leaf decoction: LC-HRMS-MS-Qtof identification, bioactivities and bioavailability evaluation. *Food Chem. Adv*,. 1:100003. doi:10.1016/j.focha.2021.100003

Martínez-Rodríguez, A., Martínez-Olcina, M., Mora, J., Navarro, P., Caturla, N., Jones, J. (2022). Anxiolytic effect and improved sleep quality in individuals taking *Lippia citriodora* extract. *Nutrients*, 14(1):218. doi:10.3390/nu14010218

McKenna, D. J., Ruiz, J. M., Hoye, T. R., Roth, B. L., Shoemaker, A. T. (2011). Receptor screening technologies in the evaluation of Amazonian ethnomedicines with potential applications to cognitive deficits. *J. Ethnopharmacol.*, 134(2):475–492. doi:10.1016/j.jep.2010.12.037

Mirjalili, M. H., Moyano, E., Bonfill, M., Cusido, R. M., Palazón, J. (2009). Steroidal lactones from *Withania somnifera*, an ancient plant for novel medicine. *Molecules*, 14(7):2373–2393. doi:10.3390/molecules14072373

Palhano-Fontes, F., Barreto, D., Onias, H., Andrade, K. C., Novaes, M. M., Pessoa, J. A., et al. (2019). Rapid antidepressant effects of the psychedelic ayahuasca in treatment-resistant depression: a randomized placebo-controlled trial. *Psychol. Med.*, 49(4):655–663. doi:10.1017/S0033291718001356

Perez-Pinero, S., Munoz-Carrillo, J. C., Echepare-Taberna, J., Munoz-Camara, M., Herrera-Fernandez, C., Garcia-Guillen, A. I., et al. (2024). Dietary supplementation with an extract of *Aloysia citrodora* (lemon verbena) improves sleep quality in healthy subjects: a randomized double-blind controlled study. *Nutrients*, 16(10):1523. doi:10.3390/nu16101523

Popat, A., Shear, N. H., Malkiewicz, I., Stewart, M. J., Steenkamp, V., Thomson, S., et al. (2001). The toxicity of *Callilepis laureola*, a South African traditional herbal medicine. *Clin. Biochem.*, 34(3):229–236. doi:10.1016/s0009-9120(01)00214-4

Puniani, E., Cayer, C., Kent, P., Mullally, M., Sánchez-Vindas, P., Álvarez, L. P., et al. (2015). Ethnopharmacology of *Souroubea sympetala* and *Souroubea gilgii* (Marcgraviaceae) and identification of betulinic acid as an anxiolytic principle. *Phytochemistry*, 113:73–78. doi:10.1016/j.phytochem.2014.02.017

Raffauf, R. F., Shemluck, M. J., Le Quesne, P. W. (1991). The withanolides of *Iochroma fuchsioides*. *J. Nat. Prod.*, 54(6):1601-1606. doi:10.1021/np50078a017

Rashidian, A., Farhang, F., Vahedi, H., Dehpour, A. R., Mehr, S. E., Mehrzadi, S., et al. (2016). Anticonvulsant effects of *Lippia citriodora* leaves ethanolic extract. *Int. J. Prev. Med.*, 7:97. doi:10.4103/2008-7802.187251

Reckweg, J., Mason, N. L., van Leeuwen, C., Toennes, S. W., Terwey, T. H., Ramaekers, J. G. (2021). A phase 1, dose-ranging study to assess safety and psychoactive effects of a vaporized 5-methoxy-N, N-dimethyltryptamine formulation (GH001) in healthy volunteers. *Front. Pharmacol.*, 12:760671.

Renner, U. D., Oertel, R., Kirch, W. (2005). Pharmacokinetics and pharmacodynamics in clinical use of scopolamine. *Ther. Drug. Monit.*, 27(5):655–665. doi:10.1097/01.ftd.0000168293.48849.b5

Restrepo, D. A., Saenz, E., Jara-Muñoz, O. A., Calixto-Botía, I. F., Rodríguez-Suárez, S., Zuleta, P., et al. (2019). *Erythroxylum* in focus: an interdisciplinary review of an overlooked genus. *Molecules*, 24(20):3788.

Ricarte, L. P., Bezerra, G. P., Romero, N. R., Silva, H. C., Lemos, T. L., Arriaga, A., et al. (2020). Chemical composition and biological activities of the essential oils from *Vitex agnus-castus*, *Ocimum campechianum* and *Ocimum carnosum*. *An Acad .Bras. Cienc.*, 92(suppl 1):e20191346.

Rodríguez, J. L., Mateos, R., Palomino, O., Fernández-Alfonso, M. S., Ramos-Cevallos, N., Inostroza-Ruiz, L., et al. (2023). Cytoprotective-antioxidant effect of *Brunfelsia grandiflora* extract on neuron-like cells. *Appl. Sci.*, 13(22):12233. doi:10.3390/app132212233

Schultes, R. E., Holmstedt, B. (1968). De plantis toxicariis e mundo novo tropicale commentationes II: The vegetal ingredients of the myristicaceous snuffs of the northwest Amazon. *Rhodora*, 70(781):113–160.

Sequeda-Castañeda, L. G., Modesti Costa, G., Celis, C., Gamboa, F., Gutiérrez, S., Luengas, P. (2016). *Ilex guayusa* Loes (Aquifoliaceae): Amazon and Andean native plant. *Pharmacolog. Online*, 3:193–202.

Shaheen, H. M., Ali, B. H., Alqarawi, A. A., Bashir, A. K. (2000). Effect of *Psidium guajava* leaves on some aspects of the central nervous system in mice. *Phytother. Res.*, 14(2):107–111.

Sora, I., Hall, F. S., Andrews, A. M., Itokawa, M., Li, X. F., Wei, H. B., et al. (2001). Molecular mechanisms of cocaine reward: combined dopamine and serotonin transporter knockouts eliminate cocaine place preference. *Proc. Natl. Acad. Sci. U S A*, 98(9):5300–5305. doi:10.1073/pnas.091039298

Tafurt-Garcia, G., Munoz-Acevedo, A., Calvo, A. M., Jimenez, L. F., Delgado, W. A. (2014). Componentes volátiles de *Eriope crassipes*, *Hyptis conferta, H. dilatata, H. brachiata, H. suaveolens* y *H. mutabilis* (Lamiaceae). *Bol. Latinoam. Caribe Plantas Med. Aromát.*, 13(3):254–269.

Torres, C. M., Repke, D. B. (2006). *Anadenanthera*: visionary plant of ancient South America. *J Psychoactive Drugs*, 38(4):381–393.

Venâncio, E. T., Rocha, N., Rios, E., Feitosa, M., Linhares, M., Melo, F., et al. (2011). Anxiolytic-like effects of standardized extract of *Justicia pectoralis* (SEJP) in mice: involvement of GABA/benzodiazepine receptor. *Phytother.. Res*, 25(3):444–450. doi:10.1002/ptr.3274

Weckerle, C. S., Stutz, M. A., Baumann, T. W. (2003). Purine alkaloids in *Paullinia*. *Phytochemistry*, 64(3):735-742.

Yuan, C. S., Mehendale, S., Xiao, Y., Aung, H. H., Xie, J. T., Ang-Lee, M. K. (2004). The gamma-aminobutyric acidergic effects of valerian and valerenic acid on rat brainstem neuronal activity. *Anesth. Analg.*, 98(2):353–358. doi:10.1213/01.ANE.0000096189.70405.A5

Zakavi, F., Hagh, L. G., Daraeighadikolaei, A., Fazelipour, S., Komeili, G., Farkhondeh, T. (2021). Juglone: a review of biological activities and health effects. *Avicenna J Phytomed.*, 11(2):116–130. doi:10.22038/AJP.2020.16501

Zhao, J., Nakamura, N., Hattori, M., Kuboyama, T., Tohda, C., Komatsu, K. (2002). Withanolide derivatives from the roots of *Withania somnifera* and their neurite outgrowth activities. *Chem. Pharm. Bull.*, 50(6):760–765. doi:10.1248/cpb.50.760
